# Internal relocation as a relevant and feasible adaptation strategy in Rangiroa Atoll, French Polynesia

**DOI:** 10.1038/s41598-022-18109-8

**Published:** 2022-08-19

**Authors:** Virginie K. E. Duvat, Alexandre K. Magnan, Lydie Goeldner-Gianella, Delphine Grancher, Stéphane Costa, Olivier Maquaire, Gonéri Le Cozannet, Lucile Stahl, Natacha Volto, Cécilia Pignon-Mussaud

**Affiliations:** 1 UMR LIENSs 7266, La Rochelle University-CNRS, 2 rue Olympe de Gouges, 17000 La Rochelle, France; 2 Institute for Sustainable Development and International Relations, 27 rue Saint-Guillaume, Sciences Po, 750005 Paris, France; 3 Laboratory of Physical Geography, University Paris 1 Panthéon-Sorbonne, 75005 Paris, France; 4 Laboratory of Physical Geography, CNRS, 75005 Paris, France; 5 Normandie Univ, Unicaen, CNRS, LETG, 14000 Caen, France; 6 DRP R3C, BRGM, 45000 Orléans, France

**Keywords:** Climate-change impacts, Climate-change adaptation, Climate-change policy

## Abstract

Atoll islands face increasing coastal risks (coastal erosion and marine flooding) due to climate change, especially sea-level rise. To face increasing coastal risks, various adaptation options are considered by atoll countries and territories, including in particular hard protection (preferred option to date), Nature-based Solutions (increasingly used) and island raising (considered a longer-term solution and a potential alternative to international migration, e.g. in the Maldives). Internal relocation within the same atoll island or atoll, which refers to long-term community movement from one threatened island area or island to a safer island area or island, has previously been disregarded by scholars as a potentially relevant climate adaptation strategy. However, in low-lying coastal areas, it offers real potential to address the dual context of increasing climate risks and the shrinking of the solution space. This paper assesses the potential of internal relocation for atolls by applying to Rangiroa Atoll, French Polynesia, Central Pacific, a two-fold assessment framework questioning its physical relevance (*are some islands high enough to host settlements in the future?)* and its societal feasibility (*are the political-institutional and socio-economic conditions in place? Are people willing to relocate*?). The findings show that internal relocation is both relevant and feasible on Rangiroa Atoll and should therefore serve as a pillar to develop robust in situ adaptation pathways in this atoll.

## Introduction

Atoll islands are highly vulnerable to climate change because they are low-lying (1.5–4 m above Mean Sea Level, MSL), small (generally < 1 km^2^) and dependent on climate-sensitive coral reefs. The combination of sea-level rise (SLR), changing storm wave height, and reef ecosystem decline under ocean warming and acidification, is projected to increase atoll island flooding and erosion over the twenty-first century^1–3^. Flooding already has adverse consequences on freshwater availability, subsistence and commercial activities, infrastructure and buildings in atoll islands^4,5^. Further increases in flood frequency, intensity, and spatial extent, as well as the decline of the reef ecosystem, will likely render some atoll islands uninhabitable from the mid-century^6,7^.

Four main adaptation responses are currently used to contain climate risk in atoll contexts. The prevalent option is island ‘fortification’ that is, protection using engineered structures. This option has failed in many locations and has detrimental side effects on coral reefs, beaches, and other ecosystems^8–12^. The second adaptation option, which is inapplicable in urban environments experiencing altered ecosystems, consists of using Nature-based Solutions (NbS), including the protection, sustainable management, restoration or creation of buffering ecosystems^3,8,13,14^. The third adaptation option, increasingly considered in atoll contexts, is island raising^15^. This option consists in creating new elevated islands that are higher than natural islands, to keep humans and infrastructures out of reach of extreme sea levels (ESLs). The artificial island of Hulhumale’, Maldives, provides a real-world example^15–17^, although it was primarily built to face the demographic explosion challenge. The fourth option commonly put forward is international migration, increasingly considered a last resort option because of the failure of past experiments, its unpopularity, and limited political support from targeted host countries^18^.

Internal relocation, that is, population relocation to a higher part of the same atoll island or to a higher nearby island within the same atoll has rarely (see e.g. Ref.^19^) been considered as an adaptation strategy by scholars. Its relevance and feasibility on a given atoll have never been assessed. This is, first, because in atoll islands ‘*the room to move back is very limited’* (^20^: 134), and second, because atoll islands are generally considered to be all equally (that is, highly) susceptible to flooding^7^. Yet, insights from geomorphic studies show that some atoll islands are less flood- and risk-prone than others^6,8,21–23^. Internal relocation refers to ‘*the long-term* [at least for several decades] *movement of a community from one threatened area* [or island] *to a less risk-prone area* [or island], *in which the societal structures, legal and political systems, cultural characteristics and worldviews of this community are retained’* (^24^: 58–59). Internal relocation is therefore different from international migration in that it refers to local-scale movement of people, assets and economic activities, inland or to neighboring islands.

**Figure 1:**
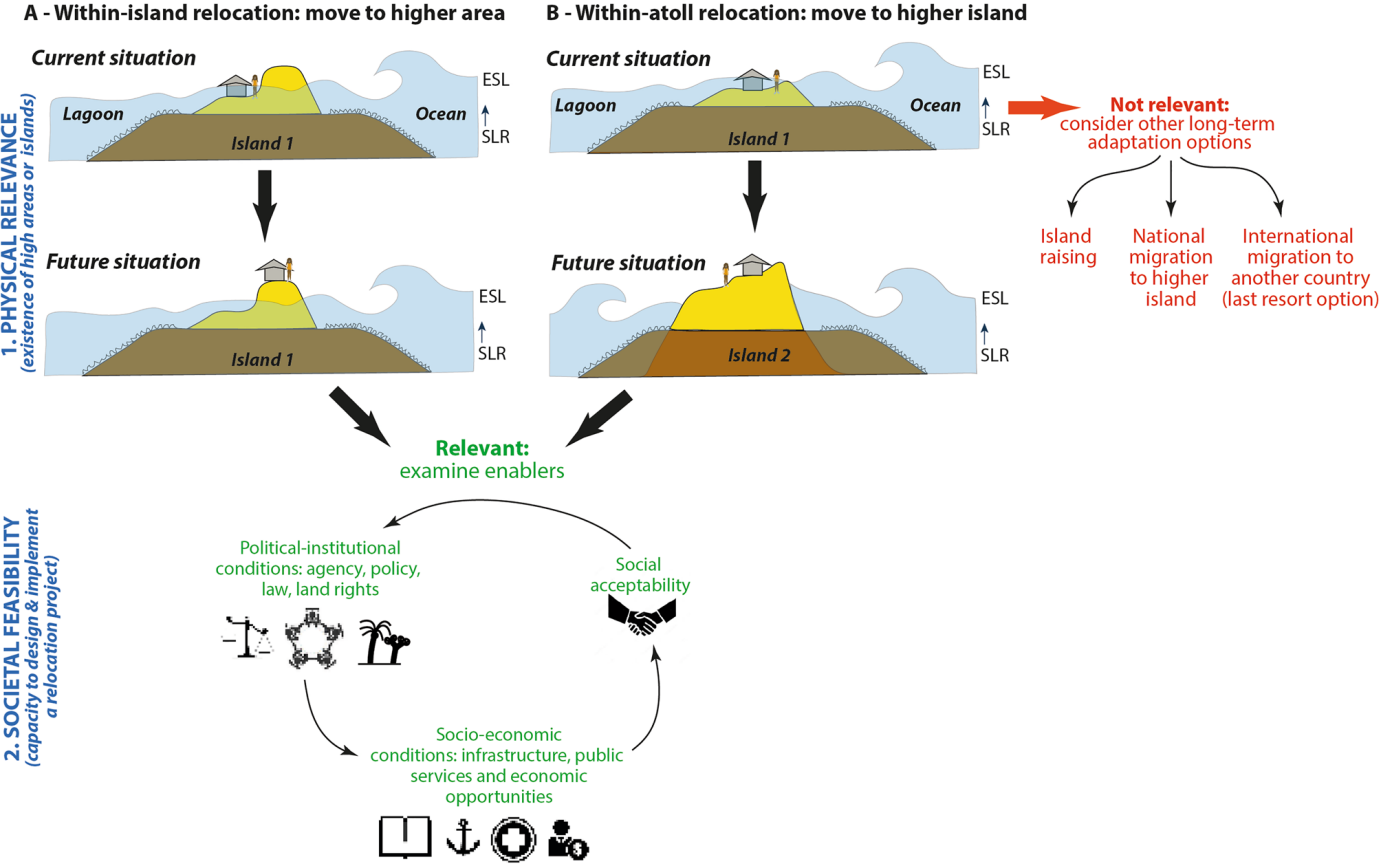
Approach of internal relocation in atoll contexts. Here, internal relocation includes moving either to a higher area on the same atoll island (**A**), or to a higher island (**B**) within the same atoll. If none of these two options are available, other long-term adaptation options should be considered, including island raising and national migration to higher distant islands or, as a last resort, abroad (international migration). If option A or B is available, internal relocation is a relevant adaptation option, and its feasibility, which relies on political-institutional and socio-economic conditions, as well as on social acceptability, should be assessed. ESL, extreme sea level. SLR, sea-level rise.

This paper assesses the potential for anticipatory and government-led internal relocation as a decisive adaptation strategy in the face of accelerating trends in climate change and SLR^25^ in atoll contexts, using the case study of Rangiroa Atoll, French Polynesia, Central Pacific. Based on a review of the scientific literature (Box 1), we identified the key challenges raised by internal relocation in atoll contexts. These challenges were pivotal to the design of our assessment framework that is twofold (Fig. 1). First, it considers the physical relevance of internal relocation (*are some islands high enough to make internal relocation a viable adaptation option?*). Second, it considers societal feasibility, including political-institutional conditions (*which policy framework, legal and planning tools, including related to land rights, are available to support the design and implementation of internal relocation?),* socio-economic conditions (*how will infrastructure, public services, and jobs be provided to the people who will relocate?*), and social acceptability (*are inhabitants willing to relocate and under which conditions?*).

We demonstrate that internal relocation is a viable adaptation option for Rangiroa Atoll and discuss the enabling conditions for successful internal relocation and the induced possible adaptation pathways for this atoll.

Box 1—internal relocation-related challenges Internal relocation first requires at-risk and non-at-risk areas to be identified through a scientific assessment establishing which communities are already and will increasingly be threatened by coastal hazards, and when^15,21,26–28^, and whether safe areas that are out of reach of future ESLs exist within the concerned atoll^2,7,23^. The results of this scientific assessment determine the physical relevance of considering internal relocation as a potentially viable adaptation option for the specific study site (here Rangiroa Atoll). In the context of our assessment, in line with previous studies (e.g. Ref.^23^), we use island flood susceptibility as the major parameter to describe physical relevance. Accordingly, if flood-free areas exist that are large enough to host threatened people and infrastructure, internal relocation is considered physically relevant (Fig. 1).

Where this prerequisite is met, three other factors must be considered that refer to political-institutional conditions, socio-economic conditions, and social acceptability, which taken together describe the societal feasibility of internal relocation (Fig. 1). The first factor is the existence of adequate policy and legal frameworks for making relocation decisions, and of political-institutional capacities to preemptively '*identify the need for and develop effective resettlement schemes’ *(^20^: 315; see also Ref.^29^). Policies and legal frameworks are needed because national and local governments are by essence responsible for the security of the population in the face of climate change^18^. Despite increasing climate risk, few countries have established guidelines for planned climate change-related internal relocation. Fiji is a rare example of an island country that has done so^18,30^. Vanuatu’s 2018 National Policy on Climate Change and Disaster-Induced Displacement also addresses planned relocation. Such policies include the capacity to raise adequate financial resources, establish the required planning tools and regulations, and implement a legal regime establishing land rights for new settlers^20,25,27,31^. Planning tools, including pre-existing plans pursuing development objectives, are required to enable relocation, through guaranteeing the funding of necessary infrastructure and services, and thereby making relocation attractive rather than an inevitable last resort option to new settlers^27^. The lack of documentation and customary titles at the original site and new location, a situation which is widespread in post-colonial Pacific Island contexts, can act as a major barrier to the transfer and security of land rights, and thereby to internal relocation^22,27^. In rural areas (including atolls) worldwide, ensuring access to land is critical in ensuring access to freshwater and food supply, and thereby to health and power^20,29,32^. To be successful, relocation must therefore be 'managed’, that is, as much as possible, anticipatory, adequately planned, and implemented using a forward-looking approach considering all concerned agencies and jurisdictions, as well as context-specificities^25,33^.

Second, socio-economic conditions need to be considered to describe the societal feasibility of internal relocation. Because relocation is a ‘*life-altering change’* (^34^: 1276), it must be thought of as a ‘*set of tools used to achieve broader* [than just risk-related] *societal goals’* (^33^: 761). Relocation must thus be ‘strategic’, that is, integrated into long-term socio-economic development goals. This can be achieved through the creation of new economic opportunities in relation with market forces, the empowerment of poor or marginalized population groups^20^, and the broader consideration of livelihoods and associated values^35,36^, demographic changes challenging quality of life and job availability in settled islands^29^, as well as equity and human right issues^37,38^. This includes providing relocated populations with the physical infrastructure and public services (healthcare, education, communication facilities) needed, seen as the conditions enabling them to rebuild their lives in destination areas^20,25,29,33,36^. See and Wilmsen^38^ use the concept of ‘*resettlement with development’ *for referring to the process of making resettlement a development opportunity. In such conditions, relocation has the potential to deliver positive outcomes for relocated communities through the improvement of their living conditions, thus avoiding labeling it as a last resort option^29^, and instead, making it a concrete climate adaptation option.

The third factor to be considered to describe the societal feasibility of internal relocation refers to the social acceptability of such an option by the relocating, hosting (if any) and remaining (if any) communities^20,39^. Because of place attachment based on historical, cultural, symbolic, and emotional tights, and existing social networks and livelihoods, it is much more complicated to move people than buildings^27^. The early consideration of livelihoods and social fabric of families, and extended families and communities related to livelihoods, is key to social acceptability^27,35,36^. Gaining the acceptance of concerned communities implies their early engagement and active participation in the design—i.e. ‘*take the risk decision participatively and collaboratively’* (Ref.^27^: 13)—and implementation of the relocation process^25,34,37^. The early involvement of communities already facing increased risk can even lead to their proactive voluntary relocation^40–42^. Trust in public authorities is also critical to the social acceptability of relocation^18^. If this condition is not fulfilled, place attachment and existing social networks may act as barriers to relocation^20,27^. Social acceptability can be determined through the consultation of concerned populations using interviews and focus groups^18,43^.

### Study area

**Figure 2:**
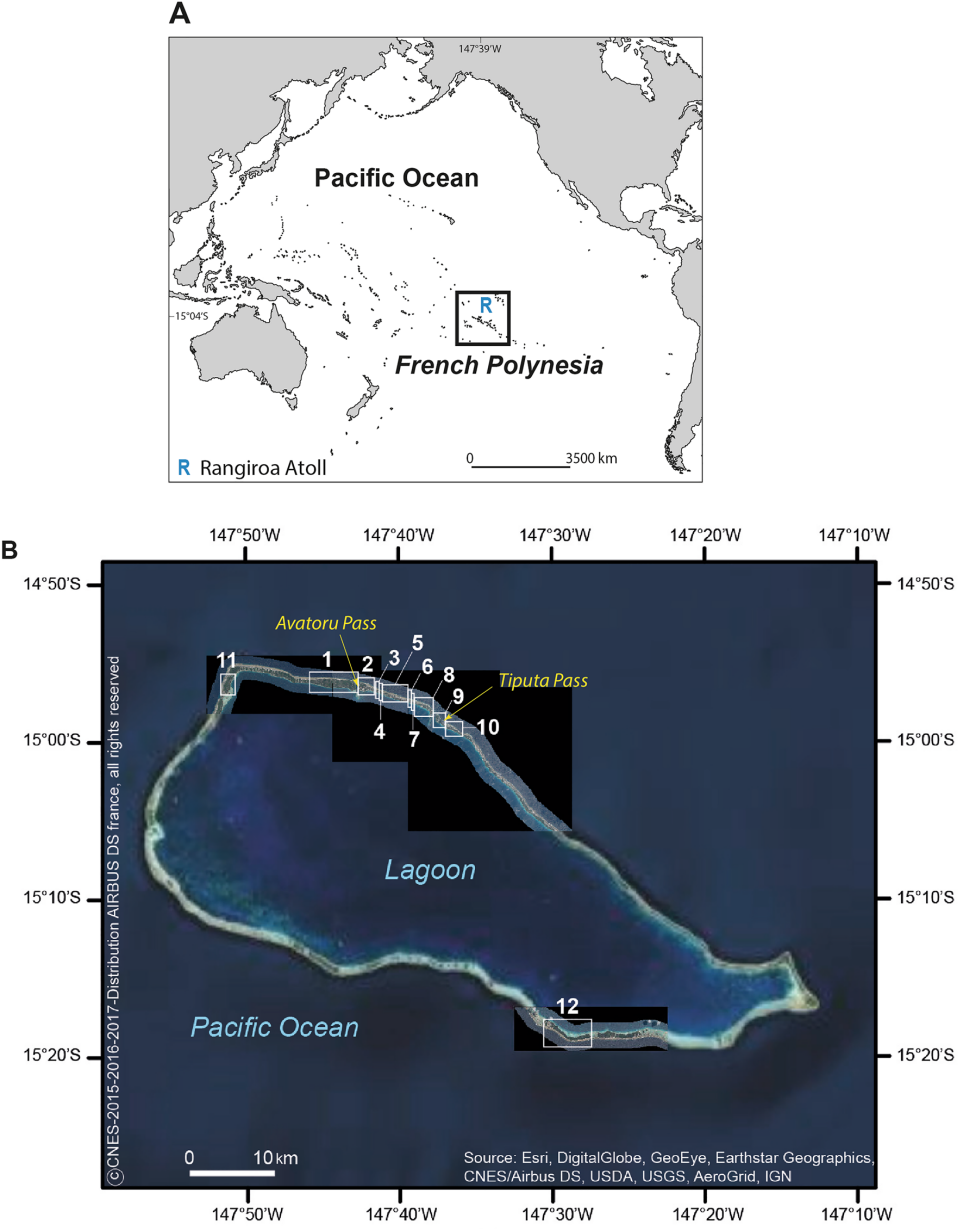
Study area. (**A**) Shows the location of Rangiroa Atoll in the Tuamotu Archipelago. On (**B**), study islands are numbered in a clockwise direction. This panel shows the location of the settled (Nos. 2–10) and unsettled (Nos. 1, 11 and 12) islands.

Rangiroa is situated in the north-western part of the Tuamotu Archipelago (Fig. 2A) and is its largest (with maximal dimensions of 79 by 32 km, 240 islands and a total landmass of 66 km^2^) and most populous (2567 inhabitants in 2012^44^) atoll. The atoll has two deep passes in the north (Avatoru and Tiputa passes; Fig. 2B), the depth of which is comprised between 31 and 34 m. Its largest islands have a land area comprised between 67 and 135 ha, and generally exhibit an ocean-facing elevated (3–5 m) beach ridge composed of coarse material (generally shingle or rubble) and a less prominent lagoon-facing sandy beach (1–3 m), which are separated by a low-lying swampy area in places.

The climate regime of the study area is controlled by the combined influence of the trade winds from the northeast to southeast directions, and of tropical and extra-tropical storms. In the Austral summer (November–March), trade winds are weak and generate moderate waves that can be disturbed by storm waves originating either from tropical cyclones (especially during El Niño phases), or from distant-source storms forming in northern latitudes. In the Austral winter (April–October), the combination of stronger trade winds and distant-source but strong southern swells generate more energetic conditions. Storm waves affecting Rangiroa are generated both by rare tropical cyclones (TCs) and depressions, and by southern distant-source storms^9,45–47^. The most devastating recent TCs were Orama and Veena (cat 3, 1983), which caused widespread flooding, coastal erosion, and ecosystem and natural resource devastation, with cascading impacts on economic activities, buildings and infrastructure, to such an extent that 10% of the population migrated to nearby atolls and Tahiti^48^. The most devastating distant-source southern swell event occurred in July 1996 and caused widespread flooding on the northern settled islands of the atoll. At Rangiroa, astronomical tides are micro-tidal and semi-diurnal, with mean and maximum tide ranges of 0.5 and 0.6 m, respectively^49,50^. Storm surges associated with TCs can have amplitudes reaching up to 1.0 m, as during TC Orama^51^. The mean and instantaneous water levels during TCs are strongly affected by the wave setup and runup. The wave setup is an increase of the mean water level due to waves breaking mainly on the reef crest, while the runup is the maximum elevation reached by waves on the island. Numerical modeling revealed that the runup under the influence of cyclonic waves (Hs: 8–12 m) respectively reach up to 4.5–5.0 m and 2.1 m above hydrographic reference on the ocean^52^ and lagoon^53^ coasts of Avatoru Island (No. 2). Under these water levels, the northern islands are affected by overtopping-induced flooding^54^. The same modelling results show that the wave setup can typically reach 1 m during a cyclonic event on the western side of Avatoru^52^. Areas located below the mean water levels corresponding to high tides, plus the storm surge, the wave setup and SLR due to climate change are exposed to overflow.

Estimated absolute SLR between 1950 and 2009 was 2.5 mm±0.5 mm/year, i.e. higher than the global mean SLR for the twentieth century, estimated to be ~ 1.8 mm/year by Church and White^55^ and 1.2 ± 0.2 mm/year by Hay et al.^56^. Median projected SLR in 2100 reaches + 0.45 m under the socio-economic pathway SSP1-2.6 and + 0.78 m under the socio-economic pathway SSP5-8.5 (Supplementary Material 1). This covers a wide range of future policies ranging from meeting the Paris Agreement and stabilizing climate warming below 2 °C (SSP1-1.9 and SSP1-2.6) to a continued increase of fossil fuels, particularly coal (SSP5-8.5), well above current intended policies. Yet, higher amounts of SLR, e.g. 1.5 m in 2100, cannot be excluded, especially if Greenland and Antarctica ice sheet melting accelerates beyond the likely projections after 2050^57^.

Rangiroa Atoll is less exposed to SLR-related risks than many other atolls worldwide for at least two reasons: (1) the coral reefs of the Central Pacific are being less quickly affected by ocean warming-induced bleaching than other atoll regions, such as the Indian Ocean^58^; (2) islands are perched on a conglomerate platform reaching up to 0.60 m in height^59^. However, although Rangiroa’s islands remain largely rural, they exhibit extensive human disturbances that have disrupted natural processes and reduced the capacity of the settled islands to naturally adapt to climate change-related pressures^9^.

This study examines twelve islands of the atoll, including the nine northern settled islands (Nos. 2 to 10; Fig. 2B) which concentrate the population, services, and infrastructure (including airport and harbors), as well as three higher^21^ unsettled rural islands (Nos. 1, 11 and 12), which were settled in the past^60^. Some of these islands (Nos. 1, 10, 12) are targeted for future development by the General Land Use Plan (*Plan Général d’Aménagement*, PGA) of Rangiroa^61^. On Rangiroa, the main economic activities are fishing (with fish exports to Tahiti), tourism (especially diving-oriented) and agriculture (subsidized copra production)^61^.

## Results

### Lessons learnt from population movements over the atoll’s history

Until the middle of the eighteenth century, the population was scattered over the atoll and all the islands included in this study were inhabited^60^. Following repeated attacks by nearby Anaa Atoll’s warriors in the second half of the eighteenth century, the scattered communities gathered around the three passes in the north (Avatoru and Tiputa passes) and north-west (where a pass existed at that time) of the atoll to better ensure their security. However, this strategy failed, and inhabitants finally took refuge in Tahiti ~ 1750. When they returned to Rangiroa ~ 1825, they spread out again across the atoll. This traditional settlement pattern was challenged again ~ 1850 when missionaries arrived and gathered the population on the northern and north-western islands in the frame of the Christianization process. However, the southern Otepipi island (no. 12; Fig. 2B) remained settled at that time. In the twentieth century, natural disasters, including the 1906 and 1983 TCs, contributed to the concentration of the population in the Avatoru-Tiputa area (islands nos. 2–9; Fig. 2B). The 1983 TC led to the permanent abandonment of Otepipi and to the out-migration of ~ 10% of Rangiroa’s population to Tahiti^48^. It is unknown whether these inhabitants returned to Rangiroa later. Moreover, official statistics reveal that since 1946 (600 inhabitants), the population of Rangiroa has increased continuously, with an acceleration since the end of the 1980s^44^, with 1300 inhabitants in 1988 and 2567 inhabitants in 2017. These findings confirm that internal and out-migration have for long been used to face climate and non-climate pressures on this (and potentially other) Tuamotu atolls.

### Is internal relocation physically relevant?

A previous study revealed that the northern settled islands generally have lower elevation indices (from 1 to 3, expect for Tiputa with 4) than the uninhabited rural islands targeted for future development which exhibit indices of 3 and 4 (see details in Ref.^21^). Most of the northern settled islands exhibit elevations < 3.74 m for all of the five variables considered (averaged island elevation, maximum ocean-side beach ridge elevation, maximum lagoon-side beach ridge elevation, lowest elevation in the inner part of the island, and highest point on the island), whereas the uninhabited rural islands targeted for future development show higher elevations (e.g. maximum ocean-side beach ridge and/or highest elevation > 6.0 m for some islands). The last century’s concentration of people and infrastructure on the northern islands has thus led to increased population and infrastructure exposure to climate pressures.

**Figure 3:**
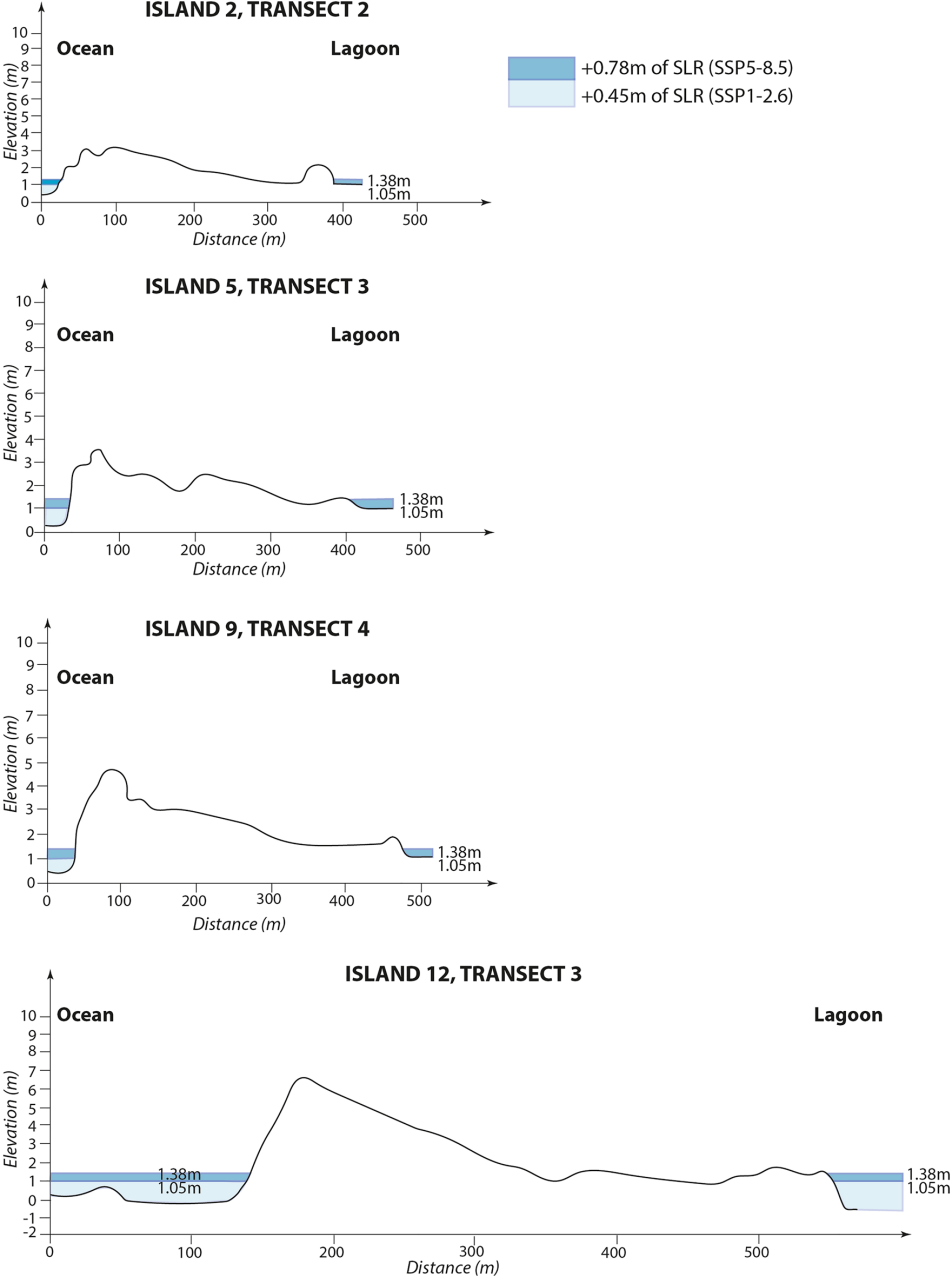
High-tide water levels under SSP1-2.6 and SSP5-8.5 climate scenarios for different island profiles around Rangiroa Atoll. This figure highlights major elevation and topography differences between islands, which result in high variations in their susceptibility to high-tide chronic flooding under SSP1-2.6 and SSP5-8.5 climate scenarios: islands Nos. 2, 5 and 9 exhibit lagoon-side land loss, whereas island No. 12’s beach ridges prevent land loss. For details on Extreme Sea Levels, see Fig. 7 in “Materials and Methods”.

Island susceptibility to high-tide chronic and cyclone-driven flooding is highly variable among study islands, and comparatively high for most of the northern settled islands (except for islands Nos. 8 and 9) compared to some uninhabited rural islands targeted for future development (Nos. 11 and 12). The extent of high-tide chronic flooding under IPCC SLR scenarios SSP1-2.6 and SSP5-8.5 in 2100 varies across study islands, depending on the height of their lagoon-side beach ridge (Fig. 3; Supplementary Material 5). Under SSP1-2.6, no island is significantly affected by high-tide chronic flooding, as lagoon- and ocean-side beach ridges prevent flooding. Under SSP5-8.5, two islands, including Avatoru (No. 2; which currently concentrates most of the population and public services, and the harbor infrastructure), and the island hosting the airport (No. 5) experience land loss along their low-lying lagoon coast. The flooded area reaches up to 100 m in width from the coastline in the central part of island No. 5. Under SSP1-2.6, the other settled islands exhibit no or very limited high-tide flooding along their lagoon shoreline. The two islands that show the lowest susceptibility to high-tide chronic flooding are the easternmost islands of Ohotu (No. 8) and Tiputa (No. 9; first island that was settled in the northern part of the atoll). The three uninhabited rural islands considered, two of which (Nos. 10 and 11) have relatively high ocean- and lagoon-side beach ridges, all remain flood-free under both SSP1-2.6 and SSP5-8.5 in 2100, with limited differences between these two scenarios.

**Figure 4:**
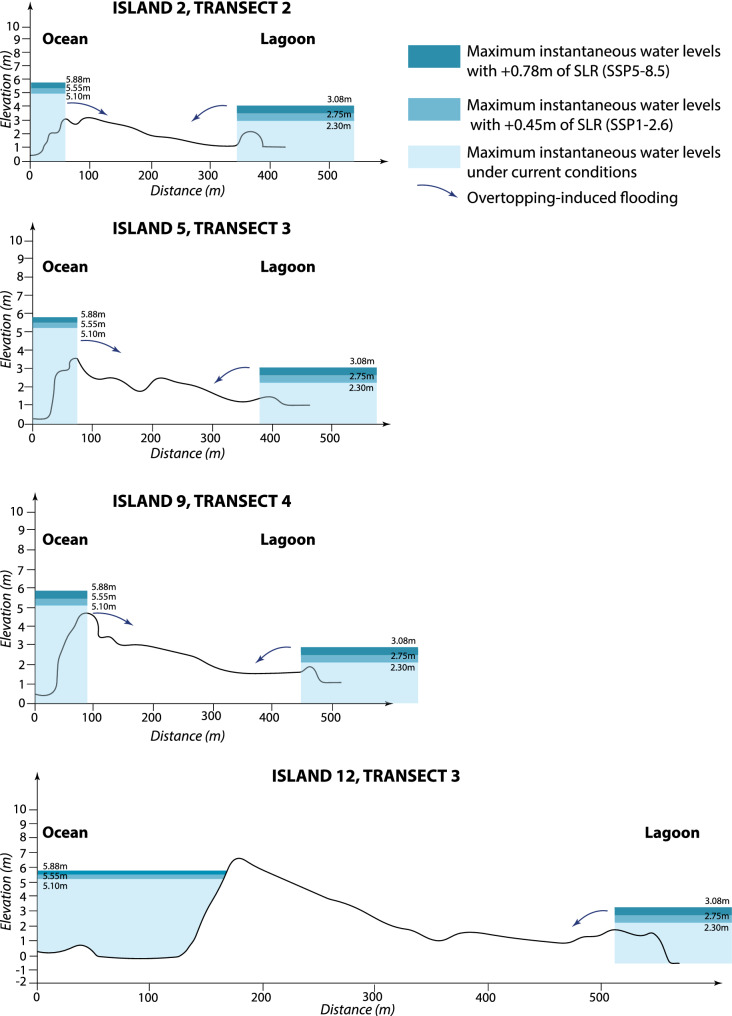
Indicative cyclone-driven maximum instantaneous water levels, as modelled on Avatoru, under SSP1-2.6 and SSP5-8.5 climate scenarios, for different island profiles around Rangiroa Atoll. As a result of major elevation and topography differences, islands Nos. 2, 5, 9 and 12 are unequally susceptible to extreme cyclone-driven overtopping under both SSP1-2.6 and SSP5-8.5: whereas islands Nos. 2, 5 and 9 are flooded over their entire surface, island No. 12 has non flooded areas along its elevated ocean side. Note that extreme water levels are based on simulations made for Avatoru (No. 2), and that the estimation of flooding on island No. 12 would require a detailed hydrodynamic study. Yet, values provided in ref. 52 are considered conservative enough to support the conclusion that island No. 12 offers the best safety potential during a TC. For details on Extreme Sea Levels, see Fig. 7 in “Materials and Methods”.

The analysis of cyclone-driven flood scenarios shows that all islands except islands Nos. 11 and 12 are flooded over their entire land area under both SSP1-2.6 and SSP5-8.5 in 2100 (Fig. 4; Supplementary Material 6). Except for island No. 9 (transect 2), the maximum elevation of these islands is lower than the maximum instantaneous water levels under intense TC conditions for present days sea levels. Given the intense wind, waves, and flow velocity conditions during a TC, flooding due to overtopping will increase risks for human life in the future. Interestingly, islands Nos. 11 and 12 have non-flooded areas under future intense cyclonic conditions. On island No. 11, these areas correspond to the highest parts of the lagoon-side sand dunes and are approximately 110 m wide. On island No. 12, the flood-free area is located along the ocean-side, where the height of the beach ridge exceeds ESLs and extends over 50–100 m in width. These findings confirm that some of the uninhabited rural islands targeted for future development are safer than the northern settled islands.

In conclusion, internal relocation from currently settled and generally very low-lying northern islands (except for islands Nos. 8 and 9 that are significantly higher than other settled islands) to higher islands, including nearby and distant uninhabited islands of the atoll, can be considered a relevant adaptation option to reduce population and human asset exposure to future marine flooding on Rangiroa Atoll.

### Is internal relocation feasible from a political-institutional perspective?

As a preliminary remark, it must be noted that French Polynesia is a French Overseas Territory with the status of a ‘Collectivité d’Outre-Mer’. This means that French Polynesia has a high degree of political autonomy from the French state since its first legal status of autonomy in 1984, which was reinforced in 1996 and 2004. French Polynesia has the competence to regulate all the fields that are not expressly assigned to the French state or to the municipalities headed by a mayor, and is especially responsible for urban and rural planning, risk prevention, and environmental management. As a result, French national relocation policies do not apply to French Polynesia.

First, official planning and legal documents and the semi-structured interviews conducted with institutional actors confirmed the absence of a dedicated policy or legal framework aimed at supporting internal (i.e. within a given island group) or external (i.e. from an atoll to Tahiti) relocation. However, interviews revealed that although it was not conceived with the aim of supporting internal relocation, the Rangiroa PGA project includes population redistribution over the atoll and could therefore contribute to climate-related internal relocation. Indeed, it targets six new islands for economic development in the uninhabited southern part of the atoll, including island No. 12, which has flood-free areas under both SSP1-2.6 and SSP5-8.5 climate scenarios under both high-tide chronic and cyclone-driven flooding (Figs. 3 and 4; Supplementary Material 5 and 6).

Second, neither human resources nor funding are currently dedicated to risk management and climate adaptation on the atoll, which prevents any relocation project to be implemented. Indeed, such projects need effective support from lead institutions, as stated by inhabitants who confessed that it should be led by the local (Rangiroa's municipality; 47% of responses) or national (i.e. the Government of French Polynesia; 24%) public authorities. It can be underlined that public contributions to the public enquiry concerning the Rangiroa PGA project have denounced the lack of financial support to adapt individual homes to climate change by raising the floor to 1.50 m. If financial support does not exist for these types of targeted measures, it is likely that it will not exist for internal relocation projects either.

Third, no legal tool exists to support land acquisition for relocation purposes. However, article D.131-1 of the urbanism Code of French Polynesia states that *General Development Plans* can delimit pre-emption zones, especially for building up land reserves. In application of this article, Rangiroa PGA project indicates that the municipality plans to establish pre-emptive rights to acquire land in future development areas. Interestingly, two testimonies were collected in the field, which revealed that under specific circumstances, land issues can be resolved. The first one refers to private land acquisition by the public authorities in the 1980s for general interest purposes, including the establishment of the main administrative area (town hall, school, and fire station) on Avatoru Island (No. 2) and the construction of social housing for coconut farmers on island no. 10. The second example relates to the relaxation of land tenure rules by the French Polynesia Land Tenure Service in Tubuai Island, Australes Islands, in the aftermath of TC Oli (2010) to allow for reconstruction in safer areas. The authorities accelerated the agreement procedures through the limitation of family authorization to only five signatures when ‘undivided land’ generally involves four generations and more than ten owners. Such past experiences could lay the ground for more long-lasting changes in administrative procedures in a way they become climate adaptation compatible, for example to revise building location regulations according to new SLR projections. Although the Rangiroa PGA project (Ref.^61^) indicates that the municipality plans to establish pre-emptive rights to acquire land in future development areas, it however remains vague in terms of the associated financial means, stating that ‘*it will be necessary to put in place a financial plan to respond to the opportunities that will arise*'. As a result, to date, no financial plan has yet been established to allow land acquisition, nor any specific financing mechanism exists to raise funds for the creation of new development centers and settlements. A rather case-by-case approach is planned that is not compatible with the basics of ‘managed and strategic retreat'^33^.

Fourth, the analysis of planning documents (Ref.^65^) revealed that internal relocation is not included in the vision of climate adaptation of institutional actors. This aligns with recent findings that institutional actors from the Government of French Polynesia did not view climate change as a danger, nor as a priority, because they face much more urgent problems, such as unemployment or waste management, daily^62,63^.

Collectively, the absence of a dedicated policy or legal framework aimed at supporting internal relocation, of dedicated human resources and funding, of legal tools aimed at supporting land acquisition for relocation purposes, and of internal relocation in institutional actors’ vision of climate adaptation indicate that today the political-institutional feasibility of internal relocation is rather limited.

### Is internal relocation feasible from a socio-economic perspective?

The Rangiroa PGA draft document recognizes the major role that tourism has played in both socio-economic development and the construction of major infrastructure (e.g. international airport in 1966): ‘*tourism has been a strong catalyst for the economic growth of the municipality’* (Presentation report of the General Land Use Plan of Rangiroa, 2017). It therefore uses tourism as a lever for socioeconomic development through job provision in the six southern targeted islands (including No. 12), where guest houses and hotels, physical infrastructure (including a harbor, causeways between concerned islands, service roads, etc.), and the provision of public services are planned. Although this PGA project is not a relocation plan, these findings show that public actors are aware of the need to equip future settled areas with all facilities, which could help to overcome socio-economic barriers related to internal relocation.

### Is internal relocation socially acceptable?

The focus groups revealed that land tenure conflicts, which are widespread on Rangiroa, might act as a social barrier to internal relocation. A participant explained: ‘*if some people need land, they should make a request to their own family* [but such requests are not always successful, in particular because] *today, elders mistrust the youth, which increases land-related tensions;* [as a result] *when you get a land plot from your family, you necessarily cling to it as if it was a treasure, because you might never get another one,* [and] *when you have a place, you won’t move, because it is too complicated, due to undivided land’*. This view was supported by another participant who mentioned the possibility to buy land, which was confirmed by our own observations that land plots were for sale in the northern settled islands. Except for a few focus group participants who freely expressed themselves, other participants refused to approach the land tenure taboo issue. Focus groups also revealed that despite participants observed significant climate (e.g. warmer sea, warmer climate, change in seasonality and rainfall) and environmental (e.g. decreased fish stocks, increased coral bleaching, increased coastal erosion) changes over the past decades, most of them considered climate change not a major problem for the moment. Some of them confessed that this would not make sense to consider internal relocation, because some environmental changes are caused by inadequate human practices and climate change impacts are perceived still limited on the atoll. Last, other participants proved fatalistic: ‘*Anyway, this is God who decides, so’.*

The social survey allowed us to dive deeper into climate change perception and relocation. Seventy-four percent of 102 interviewees agreed that SLR might become a threat for the atoll in 2050. When asked if the first line residents who would be affected would at that time accept to move to another area, 43% of interviewees responded *yes*, whereas 9% set conditions and 48% were uncertain. According to interviewees, if relocation was required and was easily doable, inhabitants would preferably move to another area on the atoll (28% of responses), to the mountainous islands of Tahiti, Moorea, or the Marquesas Islands (29%), or to the nearby elevated reef island of Makatea (16%). However, 27% of interviewees did not answer or did not know where people would prefer to move.

Between 50% (for island No. 12) and 62% (for island No. 1) of interviewees have already been to the distant unsettled rural islands targeted for future development in the PGA, with ~ 30% of them having family land there. When asked if inhabitants would accept to move to these islands if SLR would force them to move, 43%, 38% and 30% responded positively for island Nos. 1, 10 and 12, respectively, if jobs and facilities are provided. However, 21% of interviewees did not know what to respond and another 20% responded that inhabitants would refuse to move to these islands.

When asked about their personal position about relocation, only 34% of interviewees provided a response. Twenty-three out of the thirty-five respondents (66%) said they would accept to move to islands Nos. 1, 10 and 12 if it was required and easily feasible. When asked how to make relocation possible, 48% of respondents said that their family would provide them with a land plot, whereas 29% declared that they could buy or rent a piece of land and 43% admitted that they did not know.

Collectively, these findings suggest that internal relocation could be implemented in 2050, provided that the project is led by the public authorities (71%) and involves inhabitants (87%), and that the land-related barrier is overcome.

## Discussion

### Internal relocation as an adaptation option for Rangiroa Atoll

**Figure 5:**
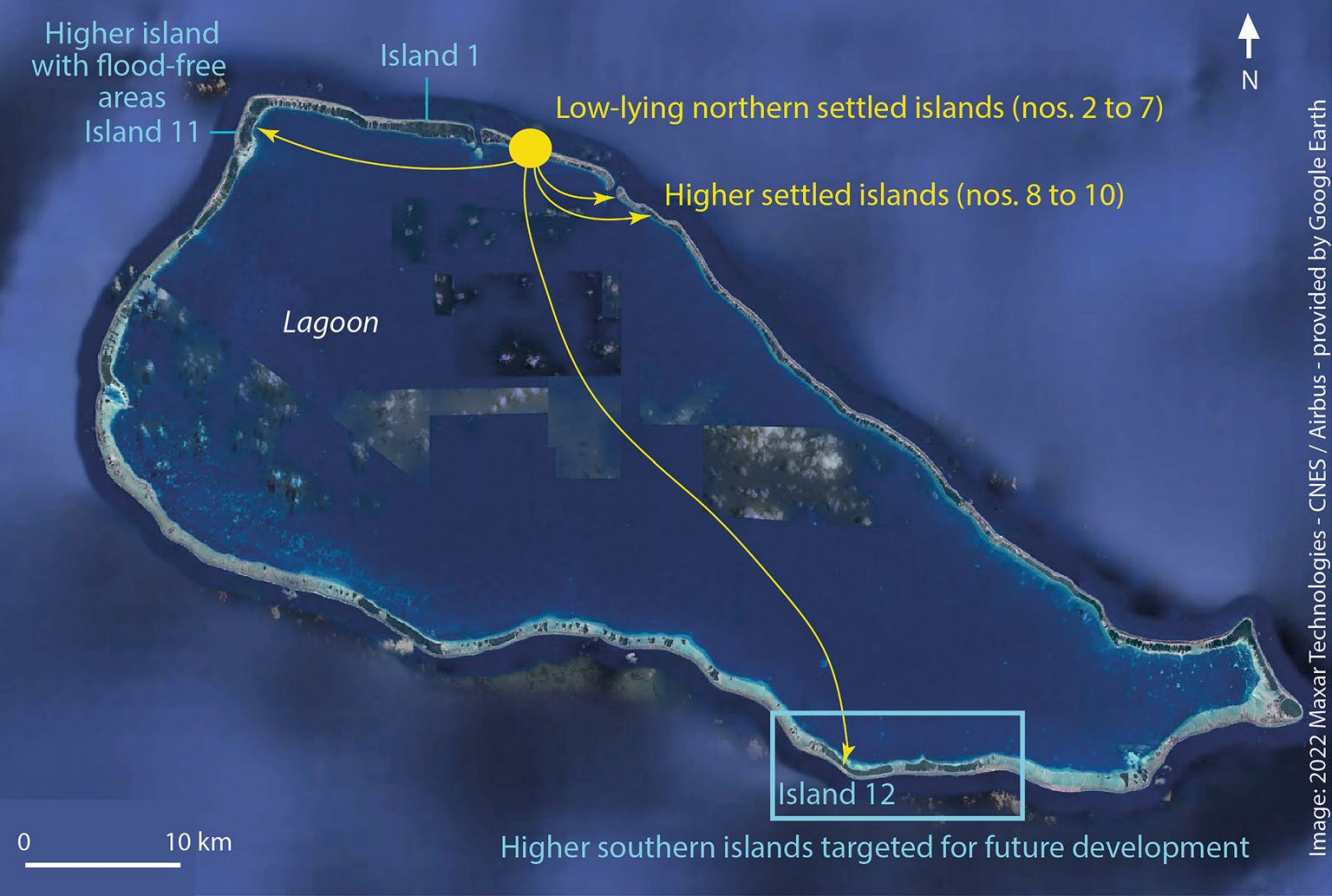
Synthesis on internal relocation opportunities on Rangiroa Atoll. This figure shows that internal relocation opportunities are located (i) in the southern unsettled islands targeted for future development by Rangiroa’s General Development Plan (see Fig. 8 in “Materials and Methods” for details), especially in island No. 12 which is much higher than the northern settled islands and has flood-free areas under extreme cyclonic conditions, (ii) in island No. 11 which has high sand dunes on its lagoon side, and (iii) in eastern settled islands (Nos. 8–10) which are significantly higher than western settled islands and remain flood-free under high-tide chronic flooding conditions.

The example of Rangiroa, French Polynesia, shows that the internal relocation of settlements, infrastructure and economic activities, from the northern settled islands exhibiting high susceptibility to flooding (principally island No. 2 and secondarily island No. 5) to nearby already settled (especially islands Nos. 8, 9 and 10) and distant uninhabited (islands Nos. 11 and 12) islands that are less flood-prone, would significantly reduce population and human assets’ exposure to climate change impacts (Fig. 5). On Rangiroa as in other atoll territories and countries (e.g. Tuvalu and Kiribati) where capitals were established based on geopolitical or economic (and not on geomorphic) considerations^64^, the capital island (Avatoru, No. 2, on Rangiroa) is highly exposed to flooding. Internal relocation to higher islands would therefore considerably reduce human exposure to flooding, including under SSP5-8.5, by prioritizing the settlement and development of some nearby (Nos. 8–10) and distant (Nos. 11 and 12) islands. Another main advantage of settling and developing the latter two islands is that they would not flood over their entire surface during intense cyclonic events in 2100, even under SSP5-8.5. In the case of Rangiroa and other atolls located in regions where TCs are rare, high-tide chronic flooding represents, from a climate perspective, the highest risk faced under climate change as it would affect land availability, which represents the central pillar for atoll island habitability^6^. Based on the lessons learnt from the most intense TCs that have already affected the atoll^47,48,54^, we assume that the disruption caused by extensive cyclone-induced flooding and wind-driven destruction would probably not lead to the permanent depopulation of the atoll in the future.

Internal relocation is not only relevant from a physical perspective, but it is also feasible from a societal perspective. The findings show that internal relocation would be acceptable for the population in 2050 under increased flooding, with 52% of interviewees responding positively and 28% of residents preferring within-atoll relocation to external relocation, provided that the project is led the local public authorities (71% of opinions), involves the population through a participatory approach (87%), and meets the socioeconomic expectations of inhabitants by providing new settlers with adequate jobs and easy access to infrastructure and public services. The municipality’s approach to the atoll’s socioeconomic development, as included in the PGA project*,* meets these conditions.

### Key challenges ahead

The design and implementation of an internal relocation plan on Rangiroa however faces four major challenges that are illustrative of the situation of atolls more broadly. The first and strongest challenge relates to the political will of both national and local public authorities to put climate change on the agenda^62,63^ and engage in transformational adaptation, here through an anticipatory resettlement strategy. In line with previous studies^65,66^, our findings show that both institutional actors and inhabitants do not consider climate change as a priority. The absence of a sense of urgency in the face of climate change impacts therefore acts as a major barrier to adaptation on Rangiroa and other Tuamotu atolls. This major barrier should be addressed by a national policy and a legal framework –including dedicated institutions, planning tools and regulations, and a legal regime establishing land rights for new settlers– aimed at facilitating relocation decisions. This ‘soft’ limit to relocation is however a serious one, given that implementing managed retreat can take decades. In the Pacific region, because it has established guidelines for planned climate change-related relocation and has already experienced this strategy^30,43,67,68^, Fiji could show the way forward.

The second challenge raised by internal relocation relates to local human capacities. In French Polynesia as in many other small island countries and territories, available human capacities assigned to environmental and climate-related matters are numerically insufficient and untrained^9^. National institutional actors acknowledged to have limited training on climate change issues and identified the need to strengthen their capacities to tackle such issues through theoretical training and the development of tailored coastal climate services^62,63^. Here we show that notwithstanding the limitations of our approach, it is possible to deliver policy relevant projections for the future exposure of atoll islands to flooding. Future climate services could consider implementing more detailed hydrodynamic flood modelling to provide more precise periods of time by which each island becomes more critically exposed to high-tide flooding and overtopping during cyclones. Yet, research on island morphodynamics in atoll islands with complex geological inherited features^59^ would be needed here.

The third challenge relates to the capacity of French Polynesia atolls (and other atoll countries and territories) to raise adequate, dedicated, and long-term financial resources to effectively implement relocation projects^3^. This is even more so as government-led planned relocation is a long process involving three main phases, namely the preparation, the implementation, and the cleanup phases, which can all together take decades^25^.

Ultimately, this study emphasizes that these three main barriers might be more difficult to overcome than the generally brought to the forefront land-related challenge. Indeed, solutions have already been found several times in the past, including on Rangiroa Atoll, to break down the land-related barrier in case of emergency (i.e. to support post-cyclone reconstruction) and when the general interest was implicated.

### Towards internal relocation-based adaptation pathways in atolls

**Figure 6:**
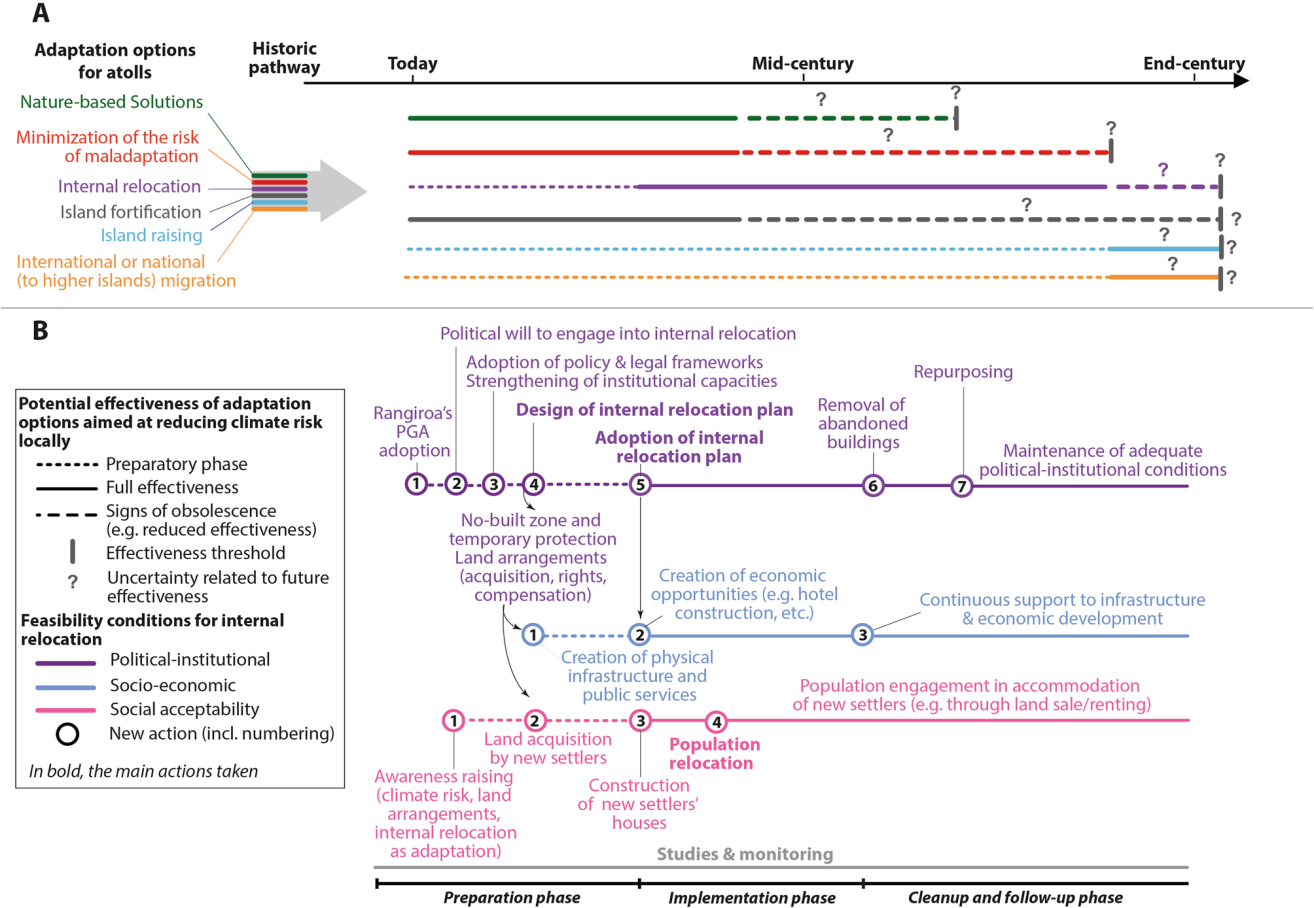
An internal relocation-oriented adaptation pathway for Rangiroa Atoll, French Polynesi*a* (inspired by Refs.^25,70,71^). (**A**) presents an adaptation pathway for atolls that considers the six available adaptation options. It proposes a sequencing of adaptation options considering short-term options that allow to buy time (including Nature-based Solutions, minimization of the risk of maladaptation, and island fortification using engineered protection structures along shorelines) until the public authorities are ready to implement a long-term internal relocation plan. Island raising (still highly uncertain from a technical perspective because atoll island substratum is highly permeable) and/or out-migration (to higher islands in French Polynesia in the case of Rangiroa) are considered last resort adaptation options. (**B**) details the sequence of actions that are needed to implement internal (i.e. within-atoll) relocation.

Based on the findings, we conclude that internal relocation is an appropriate climate adaptation option on Rangiroa Atoll that provides an alternative to other adaptation options, including Nature-based Solutions (not used on Rangiroa despite the major contribution of local human disturbances to the weakening of the Coastal Protection Service provided by marine and coastal ecosystems; Ref.^9^), island fortification (widespread but ineffective on Rangiroa; Ref.^9^), island raising (not used on Rangiroa), and national or international migration (national in the case of Rangiroa, already used in the past; see above). These latter adaptation options are increasingly acknowledged to have important trade-offs over the long term and be potential vehicles for maladaptations^69^ and the reaching of limits to adaptation (e.g. financial and cultural; Ref.^3^). In addition, there is growing agreement within the scientific community that the range of available adaptation options, that is, the “solution space”, is expected to shrink with increased global warming, henceforth highlighting the relative increasing role of internal relocation in supporting climate adaptation^25^. The scientific literature also emphasizes that planning climate adaptation over the long-term requires to move away from the illusionary perspective of identifying one single and ideal solution to sequence a set of measures to be deployed over time, according to their mutual benefits and trade-offs. This idea is captured by the concept of “adaptation pathways” that is applied here to the case of Rangiroa (Fig. 6).

Figure 6 builds on previous studies designing risk reduction and adaptation pathways for small islands^70,71^ and coasts in general^25^ to assemble the multiple insights from this study into an internal relocation-oriented adaptation pathway for Rangiroa. First, this figure shows that internal relocation needs to be contextualized, i.e. that other options targeting ecosystems, maladaptation and island fortification (i.e. use of engineered structures), could be helpful to reduce risk until effective relocation is doable (Fig. 6A). Second, this figure shows that a series of sequenced actions are needed to set the scene for effective and long-term internal relocation of settlements. These actions are highlighted in Fig. 6B and deal with the political-institutional and socio-economic conditions as well as the social acceptability of internal relocation. Panel B highlights the key components of any internal relocation strategy, including the existence of a political will for transformational adaptation, the adoption of policy and legal frameworks (especially to overcome land tenure-related barriers), the strengthening of local authorities’ institutional capacities (e.g. trained staff) to implement the relocation plan and monitor activities all over the relocation process, and the strengthening of residents’ awareness about climate risk and the role of internal relocation as a long-term adaptation strategy.

## Conclusion

Using the example of Rangiroa Atoll, French Polynesia, we assess the potential for anticipatory and government-led internal relocation as an adaptation option for atolls, using a twofold assessment framework considering, first, its physical relevance (*are some islands high enough to host settlements in the face of future extreme sea levels over the twenty-first century?)* and second, its societal feasibility (*are the political-institutional and socio-economic conditions in place? And are people willing to relocate*?). The findings demonstrate that internal relocation constitutes a physically relevant and a socially feasible adaptation strategy to reduce population and human assets’ exposure to future marine flooding on Rangiroa Atoll over the twenty-first century. This is first because some settled and unsettled islands exhibit a significantly lower susceptibility to both high-tide chronic and cyclone-driven flooding than the central settled islands. And second, because some major political-institutional (capacity of public authorities to overcome the land tenure-related barrier) and socio-economic (existence of a forward-looking vision of atoll development projecting the creation of infrastructure, public facilities and jobs in hosting areas) conditions are fulfilled, despite the lack of dedicated policy, legal framework, and financial means to support internal relocation decisions. These are considered soft limits to internal relocation, especially as inhabitants would be willing to move in case of necessity, provided that the relocation project is led by the public authorities (71% of responses) and involves them (87%). We therefore propose an internal relocation-oriented adaptation pathway for Rangiroa (and other atolls exhibiting similar conditions), which highlights that adaptation options targeting ecosystems, maladaptation, and island fortification, would help to buy time by reducing climate risk until internal relocation is effective. This adaptation pathway identifies a series of sequenced actions among which the existence of a political will for transformational adaptation, the adoption of policy and legal frameworks, the strengthening of local authorities’ institutional capacities to implement relocation and monitor activities all over the relocation process, and the promotion of residents’ awareness about climate risk and the role of internal relocation as a long-term adaptation strategy, emerge as key components.

## Materials and methods

Based on a review of the scientific literature (Box 1 in the main text), we identified the key challenges raised by internal relocation in atoll contexts. These challenges were pivotal to the design of the methodological protocol used in this study by highlighting the major areas to be covered when assessing the physical relevance and the societal feasibility of internal relocation (Fig. 1 in main text).

### Contextualization: past settlements and population movements

Archival research allowed us to document past population movements, including forced displacement, and their drivers at the atoll scale (within-atoll population movements), the regional scale (between-atoll population movements) and the national scale (population movements between Rangiroa and Tahiti). Two data sources were used, including a major ethnographic study reconstructing the whole history of Rangiroa and describing the evolution of the culture, organization, and structure of the local community, as well as the major population movements that have occurred over the past centuries and their drivers^60^, and official statistics providing information on population change since 1946^44^. The former was used, first, to understand if the unsettled rural islands targeted for future development were settled in the past, which could be favorable to internal relocation through cultural and historical heritage and collective memory, and second, to capture within-atoll population movements (*from where to where?*) and their drivers (*climate-related or not?*). Official statistics from the Institut de la Statistique de Polynésie Française (https://www.ispf.pf/) allowed us to understand recent population change on the atoll scale.

### Assessment of the physical relevance of internal relocation

A previous study (ref.^21^) allowed us to identify which islands within the atoll are higher than the northern settled islands. The assessment of future flood-prone areas was conducted using three complementary datasets, including high resolution across-island topographic transects^21^, numerical modeling studies^52,53^ and SLR projections generated by the IPCC AR6 and provided by NASA for Papeete^57^ (data available from https://sealevel.nasa.gov/ipcc-ar6-sea-level-projection-tool?psmsl_id=1397). These regional projections include regional sterodynamic changes due to thermal expansion, ocean circulation and changing pressure and the additional water masses due to melting glaciers and ice sheets and the contribution of land water masses, considering their gravitational, rotational, and deformational effects over the Earth, which causes regional SLR^57^. Vertical ground motions in Tahiti are considered neglectable in this dataset (Supplementary Material 1). This can be debated in Tahiti depending on the location^72^, but it is consistent with the current understanding of the geological context in Rangiroa^59^. Sea-level projections are provided for different socio-economic pathways covering a wide range of climate policies. The latest IPCC report considers that there is medium confidence in these projections, except for scenarios assuming rapid Antarctic and Greenland ice-sheets melting (SSP5-8.5) that involve low confidence processes. Reference^73^ suggests that the 17th percentile of these projections is a low-end for future SLR.

**Figure 7:**
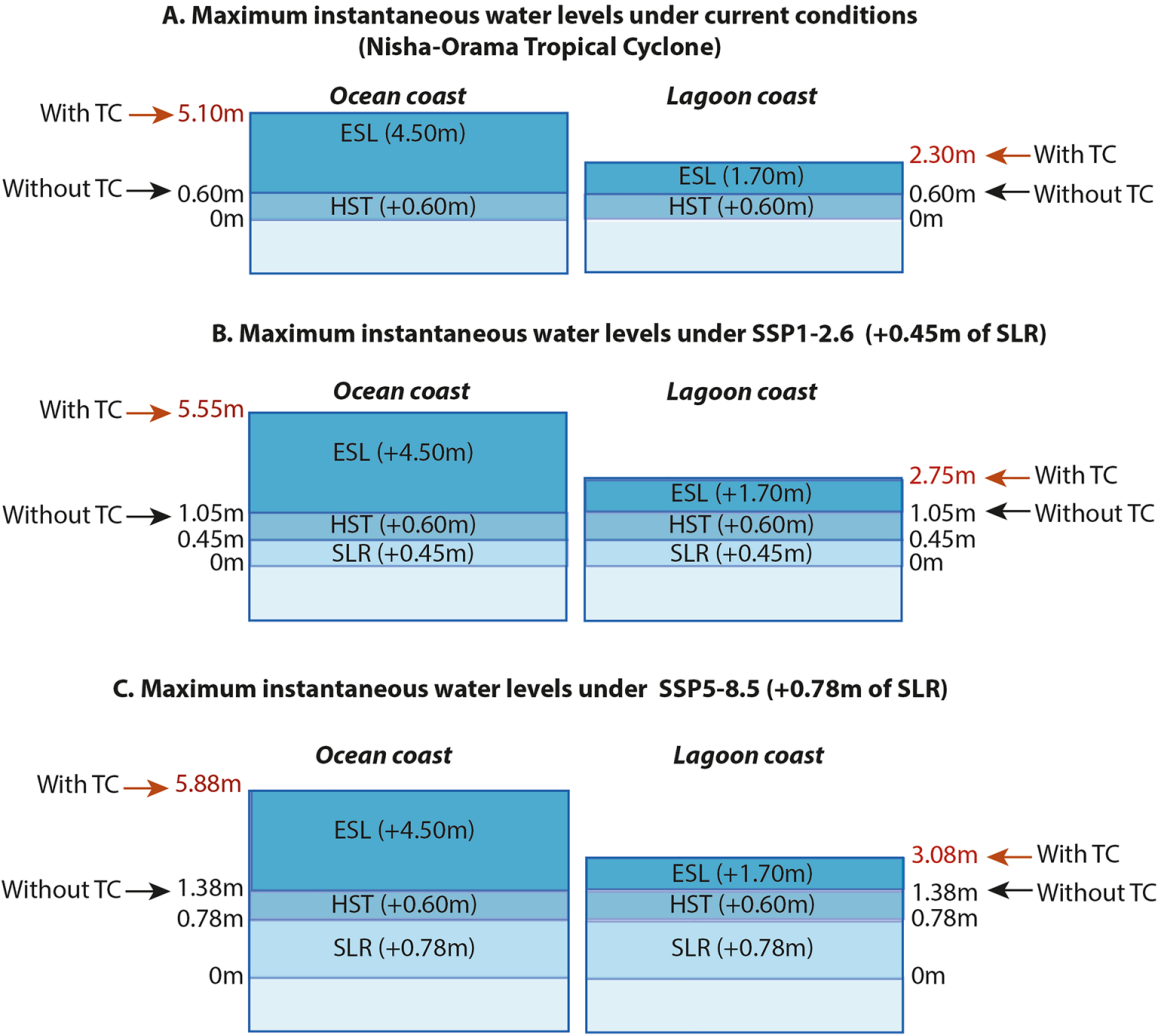
Maximum instantaneous water levels under current and future conditions on Avatoru Island, Rangiroa Atoll. Maximum instantaneous water levels are shown for a Nisha-Orama type Tropical Cyclone (the most extreme Tropical Cyclone observed on Rangiroa, in 1983). Future conditions correspond to SSP1-2.6 and SSP5-8.5 climate scenarios in 2100. HST, High Spring Tide; ESL, Extreme Sea Level associated with a Nisha-Orama-type Tropical Cyclone.

For each island, regularly spaced high-resolution across-island (i.e. perpendicular to the shoreline) topographic transects allowed to capture island topography and elevation (see details in Ref.^21^ and Supplementary Material 2 and 3). Second, numerical modeling studies^52,53^ provided instantaneous ESLs associated with the crest of the highest cyclonic swells observed on Rangiroa (i.e. during TC Nisha-Orama in 1983) under High Spring Tides (HST; + 0.60 m). These instantaneous ESLs reach respectively 5.10 m and 2.30 m above hydrographic reference (0 m) on the ocean and lagoon coasts of Avatoru Island (no. 2) (Fig. 7A). Under these water levels, the settled northern islands are flooded (see details in Ref.^54^). Third, SLR projections generated by the IPCC AR6 and provided by NASA for Papeete were used, which are of + 0.45 m and + 0.78 m in 2100 for SSP1-2.6 and SSP5-8.5, respectively, relative to 1995–2014 (Ref.^57^; Supplementary Material 1).

For each island, flood scenarios were constructed based on the superimposition of SLR, HST, and cyclone-induced ESL. Aggregated maximum instantaneous water levels in 2100 respectively reach + 5.55 m and + 5.88 m above hydrographic reference under SSP1-2.6 and SSP5-8.5 along ocean shoreline, and + 2.75 m and + 3.08 m along lagoon shoreline (Fig. 7B, C). These ESL scenarios were used to identify future flood-prone areas on topographic transects.

Because TCs are extremely rare in the study area^46,47^, we assumed that high-tide chronic flooding under SLR would likely act as the most prominent lever to internal relocation. We assessed future high-tide chronic flooding considering the HST superimposed with SLR for SSP1-2.6 (1.05 m) and SSP5-8.5 (1.38 m) (Fig. 7B, C). For these two scenarios, the superimposition of the HST and SLR generates sea levels of 1.05 m for SSP1-2.6 and 1.38 m for SSP5-8.5 above hydrographic reference in 2100 (Fig. 7B, C).

Although the approach used to assess flooding is simplistic compared to numerical modeling approaches^2,7,15^, it allows the establishment of to date missing baseline information on the flood-proneness of islands. We acknowledge that our approach neglects several processes. For example, morphological changes due to sediment transport can enhance or reduce high-tide and cyclone-induced flooding. During TCs, we sum sea level changes and values of the modelled wave setup and atmospheric contribution for similar transects from refs. 52 and 53 without considering interactions between these processes. An alternative here would be to implement a hydrodynamic model. Yet, we consider that these limitations are acceptable given the unprecedented rates of SLR since at least 6000 years in this region (Ref.^49^), which range from 6 to 10 mm/year by 2050 and could be even higher if Antarctica melts more quickly than projected.

### Assessment of the societal feasibility of internal relocation

#### Political-institutional feasibility

The analysis of risk reduction and adaptation plans and legal tools, and in-depth discussions with local and national institutional actors in charge of risk management and adaptation^62,63,65^, allowed to assess (1) whether an adequate policy and a legal framework exist for making relocation decisions and supporting the development of resettlement schemes and the implementation of internal relocation projects; whether (2) human resources, (3) funding, and (4) land tenure tools, exist to support the implementation of internal relocation; and (5) whether internal relocation is included in the vision of climate adaptation of institutional actors.

Given that in French Polynesia, no policy document or framework is specifically dedicated to climate-related internal relocation, we considered a range of documents dealing with coastal risk and climate adaptation more broadly. These documents were selected based on our own experience of the French Polynesia context—most of us have been conducting research on this territory since 2013 in the framework of various research projects: RÉOMERS (2013–2016, French overseas territories’ resilience to climate and marine related hazards in the context of climate change), funded by the French Ministry of the Environment; STORISK (2016–2021, Small islands addressing climate change: towards storylines of risk and adaptation), funded by the French National Research Agency; and INSeaPTION (2018–2021, Integrating sea-level Projections in climate services for coastal adaptation), funded by the European Union—as well as on in-depth discussions with national stakeholders^63,65^. We built on the framing used in Terorotua et al.^62^ and Magnan et al.^65^ to consider a public policy document relevant for our study when it addresses, even indirectly, coastal risks to people, infrastructure and/or economic activities, and possibly responses to these risks. We paid particular attention to two coastal hazards, i.e. coastal erosion and marine flooding. In the end, we identified four main types of documents applying either to the French Polynesia territory as a whole—i.e. the Climate and Energy Plan (*Plan Climat Énergie de la Polynésie française*) and the General Land Use Scheme (*Schéma d’Aménagement Général*)—or at a more local scale (group of islands, island, municipality, etc.)—i.e. the General Land Use Plan (*Plan Général d’Aménagement*, PGA) and the Risk Prevention Plan (*Plan de Prévention des Risques*) (see Supplementary Material 4 and 7 for details).

The material derived from the analysis of policy documents was complemented by information obtained through discussions and interviews with representatives of the national and local public authorities. During a national workshop which took place in March 2018, discussions with representatives of French Polynesia institutions involved in coastal risk management and climate adaptation and land use and urban planning provided information on (1) their views on coastal climate risks in French Polynesia, (2) the urgency to identify and implement responses, and (3) the potential role of a range of options, including internal relocation, to support adaptation in policy processes and on the ground^62,63^. The material derived from these discussions was supplemented by the results of a set of 42 semi-structured interviews conducted between mid-December 2018 and mid-February 2019 and dealing with the broader integration of climate change into coastal risk reduction policies^65^ (see Supplementary Material 7 for details). Together, these sources of information allowed us to verify the existence of a policy and of dedicated tools aimed at supporting internal relocation.

#### Socio-economic feasibility

Socio-economic feasibility was assessed through the analysis of available development plans providing information on the broader long-term (i.e. multi-decadal) development goals. In French Polynesia, this information is provided by the PGA (General Development Plan; Supplementary Material 4) for the municipalities that have adopted such a document. The PGA is adopted by the French Polynesian ministry council following municipalities’ opinion and it defines a three-to-ten years development strategy at a municipal scale (French Polynesia Planning Code, art. LP113-5). Beyond this limited period, we assumed that a PGA illustrates the vision that the local public authorities, in concert with the national public authorities, have of the longer-term future on the atoll scale. Our study therefore used this development plan to capture this vision and thereby assess the potential for future infrastructure and public services development and job provision in newly developed areas associated with internal relocation on Rangiroa Atoll.

**Figure 8:**
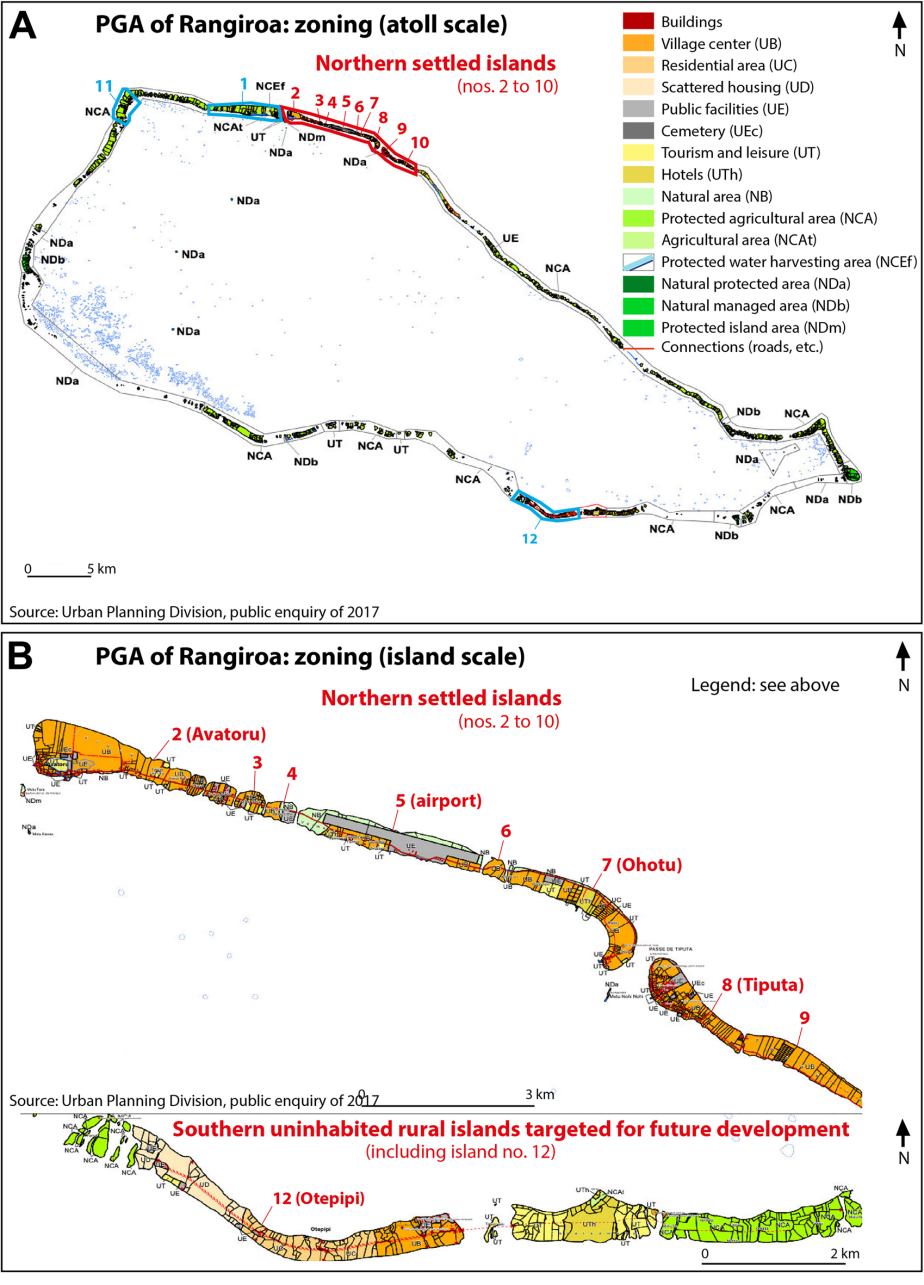
Mapping of the PGA of Rangiroa. (**A**) provides a general map on the atoll scale. (**B**) zooms in on the two critical areas considered in this study, including, on the top, the settled area, and at the bottom, the uninhabited southern rural islands (including no. 12, Otepipi) targeted for future development. (**B**) shows that these islands are planned to become a residential area (no. 12), with jobs being provided in nearby tourist (in yellow) and agricultural (in green) islands. Public facilities will be developed, including connections between islands. Source: Ref.^61^.

In practice, a PGA is designed in close collaboration between the above-mentioned public authorities, to such an extent that the plan will not be adopted if they disagree. Whilst the formal adoption competence is only held by the French Polynesian ministry council, the municipalities' opinion is followed. A PGA defines land zoning, for example for settlement (residential areas and public infrastructure), economic activities (e.g. agriculture and tourism), and cultural or environmental preservation zones where constructions are forbidden (Fig. 8). On Rangiroa, the PGA project elaborated since 2012 was submitted to public enquiry in 2017, but it has not been adopted yet.

Analyzing this development project, we paid special attention to (1) the areas/islands to be developed within the atoll; (2) the economic activities to be promoted in these areas/islands, and their potential to provide new settlers with jobs; (3) the physical infrastructure (e.g. harbor, roads, electricity and water supply) and public services (education, healthcare, etc.) anticipated in these areas/islands.

#### Social acceptability

The social acceptability of internal relocation was investigated using two complementary methodologies, including focus groups (e.g. Ref.^74^) and semi-structured interviews (e.g. Ref.^66^) with residents.

Three focus groups were co-organized with the local public authorities and some inhabitants in January 2018. Inhabitants who participated in these focus groups did so on a voluntary basis. Two focus groups took place at the city hall of the two most populous islands of the atoll, Tiputa (island no. 9; 10 participants; duration: 6’; participants’ profile: French Polynesia people) and Avatoru (island no. 2; 9 participants, duration: 50’, participants’ profile: mainly from mainland France, including secondary school teachers). The third one was organized in the catholic church of Tiputa (20 participants; duration: 45’; participants’ profile: French Polynesian people). These focus groups were prepared by the research team, which included a Polynesian PhD student who also conducted the focus groups. The operator first shared her knowledge about future SLR projections with inhabitants before introducing the general topic of climate change impacts and potential population relocation. During the focus groups, five main questions were discussed with inhabitants: *(1) If SLR became a major constraint on the atoll, do you think that affected inhabitants would accept to move to other areas within the atoll? (2) Should moving become inevitable for some inhabitants of the atoll, do you think that they would benefit from community solidarity, especially with regards to access to land? (3) In your opinion, which factors could act as barriers to within-atoll relocation? (4) Who should lead a relocation project should it become inevitable? (5) How could inhabitants be involved in such a project?* Difficulties were faced in organizing and holding these focus groups, because of the numerous family conflicts and tensions about land tenure, with many families involved in costly administrative procedures to assert their land rights. In this context, approaching land-related subjects was extremely difficult and even taboo with some parts of the population who even suspected us of approaching them on behalf of the government. This explains the low participation rate to the focus groups.

Based on the lessons learnt from the focus groups, we readjusted our investigation strategy by (1) conducting semi-structured interviews with inhabitants who were willing to discuss internal relocation; (2) avoiding placing interviewees in a situation where they would be personally concerned from the start by asking them about their general feeling about relocation before collecting their opinion. The interview guide comprised ten questions (Table 1).

**Table 1:** Interview guide used for the assessment of the social acceptability of internal relocation.

1. Do you think that the atoll will be threatened by sea-level rise in 2050?
2. Should the first line residents be affected by sea-level rise in 2050, do you think that they would accept to move?
3. If moving was required and easily doable, where would people prefer to go?
4. Do you know islands nos. 1, 10, 12?
5. How did you come to know island nos. 1, 10, 12?
6. Should sea-level rise force first line residents to move, do you think that they would accept to move to island no. 1, to island no. 10, to island no. 12, and live there?
7. In your opinion, who should lead such a relocation project on Rangiroa?
8. Should the population be involved in such a project?
9. If relocation was inevitable and easily feasible, would you personally accept to move within the atoll of Rangiroa?
10. Where would you move on the atoll?
11. Concretely, how could you manage to move there?

The semi-structured interview format had already been successful in a previous study conducted on this atoll^66^. Despite above-mentioned constraints, we ensured that the sample (1) was representative of the structure of the population in terms of gender, age distribution, and occupation (sea- and non-sea related), (2) included both first line and non-first line residents, and (3) covered the whole settled area from West to East, as well as all coastline types (lagoon, ocean, pass and hoa). Respondents were met by one of the three interviewers, either at home or where they carried out a professional or leisure activity, and interviews lasted around 30 min. One hundred and two people aged 18 and over, including 56 males (55%) and 46 females (45%), were interviewed. Middle aged individuals (between 18 and 39 years old) made the greatest contribution (51%), followed by individuals between 40 and 59 years of age (36%), and individuals > 60 years of age (13%). Eighty-seven percent of interviewees were Polynesians whereas 13% were from mainland France. Thirty-two percent carried out a sea-related professional activity whereas 68% did not. And thirty-seven percent of interviewees were first line residents whereas the remaining 63% lived inland.

## Supplementary Information

Supplementary Information.

## Data Availability

All data generated or analyzed during this study are included in this published article [and its supplementary information files].
