# Supplementary material for: Internal relocation as a relevant and feasible adaptation strategy in Rangiroa Atoll, French Polynesia
Source: Sci Rep. 2022 Aug 19;12:14183. doi: 10.1038/s41598-022-18109-8 (PMC9391457; doi:10.1038/s41598-022-18109-8)
Supplement: Supplementary file 1 — Supplementary Information. [file 41598_2022_18109_MOESM1_ESM.pdf]

Supplementary Material

Internal relocation as a relevant and feasible adaptation strategy in Rangiroa Atoll, French Polynesia

Supplementary Material 1. Projected sea-level rise

Sea level projections for 5 SSP scenarios, relative to a baseline of 1995-2014, in meters. Individual contributions are shown for the year 2100. Median values (*likely ranges*) are shown. Average rates for total sea-level change are shown in mm yr^{-1}. The SSP5-8.5 *low confidence* column incorporates a representation of the potential effect of low-likelihood, high-impact ice sheet processes that cannot be ruled out. This column shows the 17th-83rd percentile range factoring into account information from structured expert judgement and from a model incorporating Marine Ice Cliff Instability.
Percentile: Median (17th, 83rd)

	**SSP1-1.9**	**SSP1-2.6**	**SSP2-4.5**	**SSP3-7.0**	**SSP5-8.5**	**SSP5-8.5** ***Low Confidence***
Sterodynamic Sea Level	0.08 (0.03, 0.13)	0.11 (0.07, 0.16)	0.15 (0.10, 0.21)	0.18 (0.11, 0.26)	0.23 (0.16, 0.31)	0.23 (0.16, 0.31)
Glaciers	0.09 (0.07, 0.12)	0.11 (0.08, 0.13)	0.15 (0.12, 0.17)	0.19 (0.16, 0.21)	0.21 (0.18, 0.24)	0.20 (0.14, 0.26)
Greenland	0.05 (0.00, 0.11)	0.07 (0.01, 0.12)	0.10 (0.05, 0.15)	0.14 (0.08, 0.19)	0.15 (0.10, 0.21)	0.21 (0.10, 0.70)
Antarctica	0.12 (0.03, 0.29)	0.13 (0.04, 0.31)	0.13 (0.03, 0.34)	0.13 (0.03, 0.36)	0.13 (0.04, 0.40)	0.22 (0.02, 0.63)
Land Water Storage	0.03 (0.02, 0.05)	0.03 (0.02, 0.05)	0.03 (0.02, 0.05)	0.04 (0.02, 0.06)	0.03 (0.02, 0.05)	0.03 (0.02, 0.05)
Vertical Land Motion	-0.00 (-0.06, 0.06)	-0.00 (-0.06, 0.06)	-0.00 (-0.06, 0.06)	-0.00 (-0.06, 0.06)	-0.00 (-0.06, 0.06)	-0.00 (-0.06, 0.06)
Total (2030)	0.08 (0.05, 0.12)	0.09 (0.06, 0.13)	0.09 (0.06, 0.13)	0.09 (0.06, 0.13)	0.10 (0.07, 0.14)	0.10 (0.06, 0.17)
Total (2050)	0.17 (0.12, 0.24)	0.19 (0.13, 0.26)	0.20 (0.15, 0.28)	0.21 (0.16, 0.29)	0.23 (0.17, 0.31)	0.24 (0.17, 0.42)
Total (2090)	0.36 (0.24, 0.53)	0.40 (0.28, 0.59)	0.49 (0.36, 0.69)	0.57 (0.43, 0.78)	0.64 (0.49, 0.88)	0.73 (0.49, 1.42)
Total (2100)	0.39 (0.23, 0.59)	**0.45 (0.30, 0.67)**	0.57 (0.41, 0.82)	0.68 (0.51, 0.95)	**0.78 (0.59, 1.08)**	0.91 (0.59, 1.75)
Total (2150)	0.58 (0.32, 0.93)	0.72 (0.45, 1.11)	0.96 (0.64, 1.44)	1.22 (0.83, 1.73)	1.37 (0.95, 2.01)	2.12 (0.95, 5.38)
Rate (2040-2060)	4.4 (2.6, 7.0)	5.1 (3.4, 7.6)	6.0 (4.2, 8.6)	6.4 (4.6, 8.8)	7.2 (5.4, 10.0)	8.0 (5.4, 17.5)
Rate (2080-2100)	4.1 (1.4, 7.2)	5.6 (3.0, 9.0)	8.3 (5.0, 12.8)	10.6 (6.7, 15.6)	12.6 (8.6, 19.0)	16.9 (8.6, 33.8)

References:

Fox-Kemper, B., H. T. Hewitt, C. Xiao, G. Aðalgeirsdóttir, S. S. Drijfhout, T. L. Edwards, N. R. Golledge, M. Hemer, R. E. Kopp, G. Krinner, A. Mix, D. Notz, S. Nowicki, I. S. Nurhati, L. Ruiz, J-B. Sallée, A. B. A. Slangen, Y. Yu, 2021, Ocean, Cryosphere and Sea Level Change. In: *Climate Change 2021: The Physical Science Basis. Contribution of Working Group I to the Sixth Assessment Report of the Intergovernmental Panel on Climate Change* [Masson-Delmotte, V., P. Zhai, A. Pirani, S. L. Connors, C. Péan, S. Berger, N. Caud, Y. Chen, L. Goldfarb, M. I. Gomis, M. Huang, K. Leitzell, E. Lonnoy, J. B. R. Matthews, T. K. Maycock, T. Waterfield, O. Yelekçi, R. Yu and B. Zhou (eds.)]. Cambridge University Press. In press.

Garner, G. G., R. E. Kopp, T. Hermans, A. B. A. Slangen, G. Koubbe, M. Turilli, S. Jha, T. L. Edwards, A. Levermann, S. Nowikci, M. D. Palmer, C. Smith, in prep. Framework for Assessing Changes To Sea-level (FACTS). *Geoscientific Model Development*.

Garner, G. G., T. Hermans, R. E. Kopp, A. B. A. Slangen, T. L. Edwards, A. Levermann, S. Nowikci, M. D. Palmer, C. Smith, B. Fox-Kemper, H. T. Hewitt, C. Xiao, G. Aðalgeirsdóttir, S. S. Drijfhout, T. L. Edwards, N. R. Golledge, M. Hemer, R. E. Kopp, G. Krinner, A. Mix, D. Notz, S. Nowicki, I. S. Nurhati, L. Ruiz, J-B. Sallée, Y. Yu, L. Hua, T. Palmer, B. Pearson, 2021. IPCC AR6 Sea-Level Rise Projections. Version 20210809. PO.DAAC, CA, USA. Dataset accessed [YYYY-MM-DD] at https://podaac.jpl.nasa.gov/announcements/2021-08-09-Sea-level-projections-from-the-IPCC-6th-Assessment-Report.

Supplementary Material 2. Location of cross-island topographic transects.

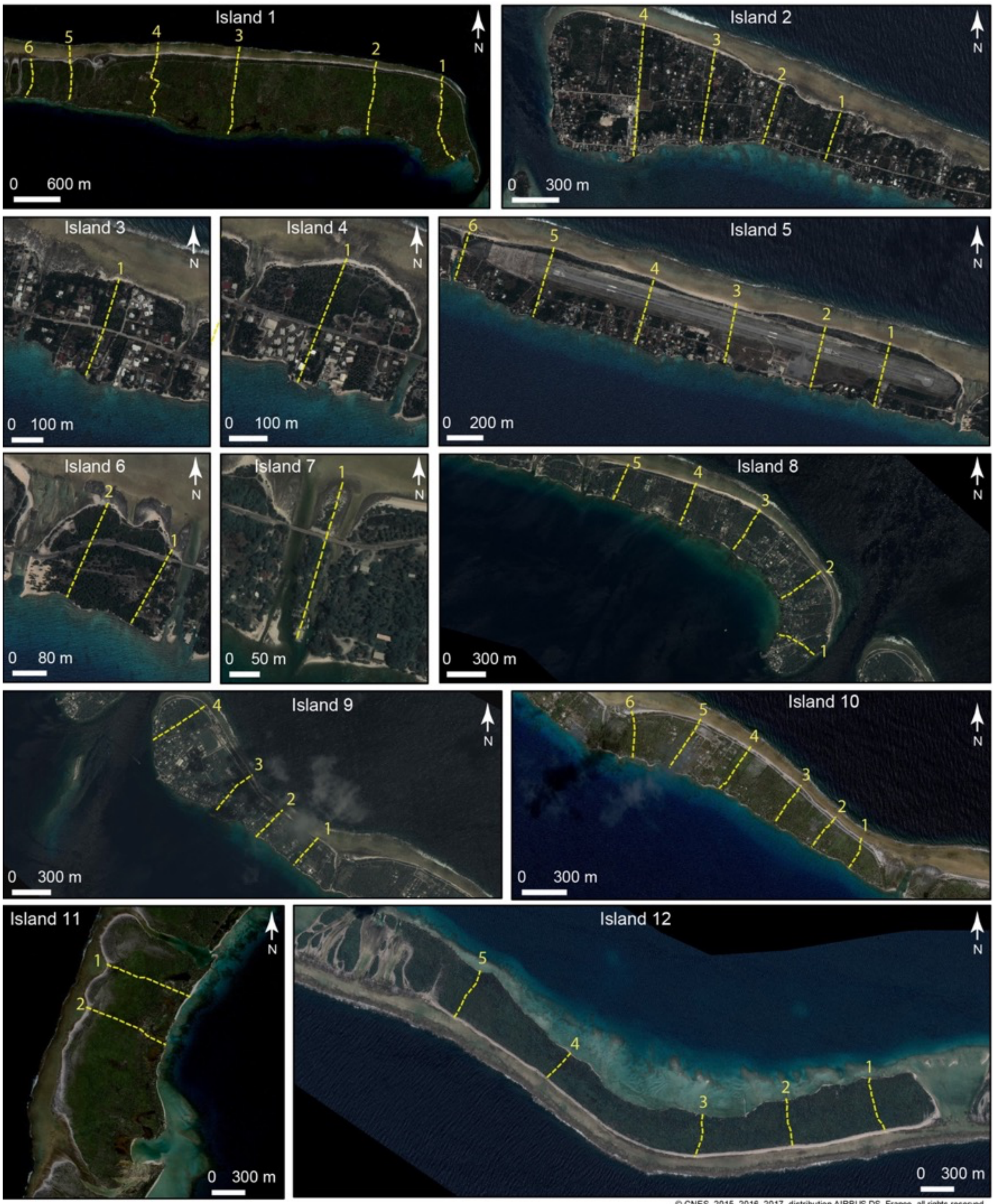

© CNES_2015_2016_2017, distribution AIRBUS DS, France, all rights reserved

Supplementary Material 3. Description of the techniques used to conduct the topographic surveys.

These topographic transects used in this study were collected between 2011 (transects collected on islands nos. 2 to 9 by the South Pacific Applied Geoscience Commission) and 2018 (transects collected on islands nos.1 and 10-12 by the authors) using GPS in RTK (Real Time Kinematic) mode.

The topographic surveys conducted on islands Nos. 1 and 10 to 12 were undertaken in October 2016 and May 2018, using standard GNSS techniques (Trimble with a base station 5700 GNSS and a mobile receiver 5800 GNSS and R8s GNSS) in RTK mode with radio link. The geodetic observations were linked to the global ellipsoid GRS80, without a geoid model, with definition of the height above the ellipsoid (HAE). We have used 3 benchmarks located on study islands nos. 1 and 10 to 12, which are well georeferenced by the French Polynesia Geodesic Network (RGPF - Réseau Géodésique de Polynésie Française). We carried out tracking from the RGPF benchmarks to other measured points with the establishment of several intermediate benchmarks materialized on the ground. These ground control points have been measured several times to define the precision of the data. Based on the numerous iterations, the precision of the coordinates of the points (X, Y, and HAE) is less than ± 0.04m, varying depending on the distance between the base station and the mobile receiver and vegetation density. The hydrographic zero has been defined using the Mean Lowest Water Level (MLWL) recorded at the SHOM (Service Hydrographique et Océanographique de la Marine) tide gauge installed at Avatoru (island No.2) in February 2009 (Lannuzel, 2010; SHOM, 2015): it is 0.39 m below the MLWL. We then subtracted 3.026 m from the HAE values to obtain the elevation referred to Z hydrographic of all points of the transect lines.

Supplementary Material 4. Short description of the four main policy documents aimed at managing coastal risks in French Polynesia.

Policy documents applicable at the whole French Polynesia scale

- The **Climate and Energy Plan** (*Plan Climat Énergie de la Polynésie française*) is a policy plan established for the 2015-2020 period. It frames the general climate strategy of the country in terms of both mitigation and adaptation. It establishes a diagnosis at the French Polynesia level primarily of energy consumption and greenhouse gas emissions, and more secondarily of climate vulnerabilities. In June 2020, the Monitoring Committee estimated that over the last five years, only one third of the implementation of the Climate and Energy Plan as a whole has been achieved, compared to two third as initially planned, and with a strong focus on mitigation activities (Alter-echo & PTPU 2020). Preliminary works will start on May/June 2022 to design a new Climate Plan that will run until 2030.
- The **General Land Use Scheme** (*Schéma d'Aménagement Général*) was approved on 24 August 2020 (Polynesian Law n° 2020-20) and defines the overall development strategy in terms of demography and economy at a 20-year timescale. It is also supposed to consider global warming issues (French Polynesia Planning Code, article LP 111-3). The General Land Use Scheme is composed of different parts, including an Overview report explaining the choices which led to the Fenua Sustainable Development Plan (*Projet d'Aménagement et de Développement Durable*, Fenua PADD). This latter establishes the settlement pattern of infrastructures in the fields of education, culture, information, transportation of people and goods, energy and sports, as well as the preferential location of port facilities, urban, industrial, agricultural, handcraft, forest and tourism activities. It contains specific rules pertaining to natural areas and landscapes (e.g. protection). The General Land Use Scheme also identifies key development challenges and general roadmaps for each of the five French Polynesia archipelagos.

Policy documents with a more local-scale focus

- The **General Land Use Plans** (*Plan Général d'Aménagement*) were initiated in the early 1960s by the French Polynesia Deliberation n° 61-44 (8 April 1961), but the first versions have only been adopted in the early 1980s (e.g. approved in 1981 in the municipality of Papara, Tahiti). To date, only a third of the 48 municipalities of French Polynesia has adopted one. The General Land Use Plans are context-specific and apply at the municipality level. While the final General Land Use Plans are approved at the Collectivity level, the municipalities are involved in the design of their respective General Land Use Plan, and the local population is consulted. A given General Land Use Plan describes a 3-to-10 year development strategy through the identification of specific functions for the different areas within the municipality, i.e. for settlement (residential buildings and public infrastructures), economic activities, or natural areas (e.g. protected areas and cultural sites). General Land Use Plans can also highlight areas where constructions are not allowed in case natural risks have been identified. For example, the General Land Use Plan of the Fakarava Atoll (Tuamotu Archipelago) adopted by the Ministerial Order n° 1422, the 26th of September 2016, prohibits constructions in the coastal area exposed to strong swells and flooding (articles UB6, UE6, UT6, NC6, ND6; Stahl 2018), and requires buildings to be raised by 1.50 m above ground level (articles UB10, UE10, UT10, NC10, ND10; JOPF 2016).
- The **Risk Prevention Plans** (*Plan de Prévention des Risques*, PPRs) are actually the most prominent documents, once adopted, to address risk prevention and reduction at the local scale. PPRs were transferred from the mainland France regulation (Deboudt 2010) into the Planning Code of French Polynesia since the Deliberation n° 2001-10 adopted on February

2001. PPRs aim at characterizing natural hazards at a sub-municipality scale in order to inform land use planning. In particular, PPRs identify areas where future constructions or activities are subject to specific rules or are prohibited, and where existing constructions must adhere to specific measures. The stringency of the requirements depends on the actual hazard level. Once approved by the French Polynesia Ministry Council, PPRs take precedence over General Land Use Plans to which they are annexed as a public utility easement. Despite their importance for risk prevention, only three PPRs have been adopted forin the Punaauia municipality in Tahiti (JOPF 2010), Rurutu (JOPF 2018) and Rimatara (JOPF 2019) in the Austral Islands archipelago. Our analysis however also considers the PRR documents that have not yet been officially approved but exist as draft documents.

References

Alter-echo & PTPU, 2020. Comité de Suivi 7, compte rendu du 15 juin 2020. Plan Climat Energie de la Polynésie française. URL: http://www.plan-climat-pf.org/library/userfiles/workgroups/1/PCE_ComiteSuivi7Synthese_20200615.pdf.

Deboudt, P., 2010. Towards coastal risk management in France. Ocean & coastal management, 53(7), pp.366-378. doi: 10.1016/j.ocecoaman.2010.04.013

JOPF (2010). Journal Officiel de Polynésie Française. Ministerial Order n°392 CM, 25 March 2010, Punaauia.

JOPF (2018). Journal Officiel de Polynésie Française. Ministerial Order n°559 CM, 5 April 2018, Rurutu.

JOPF (2019). Journal Officiel de Polynésie Française. Ministerial Order n°2901 CM, 17 December 2019, Rimatara.

Stahl L., 2018. Les défis présents et à venir des plans de prévention des risques naturels polynésiens. Études caribéennes 41. https://doi.org/10.4000/etudescaribeennes.13106.

Supplementary Material 5. High-tide chronic flooding in 2100 under SSP1-2.6 and SSP5-8.5.

High-tide chronic flooding (under High Spring Tide of +0.60m) superimposed to +0.45m (SSP1-2.6; blue line) and +0.78m of SLR (SSP5-8.5; orange line) in 2100 relative to a baseline of 1995-2014 (Fox-Kemper et al., 2021) is shown for all study islands in the following figures.

Island 1

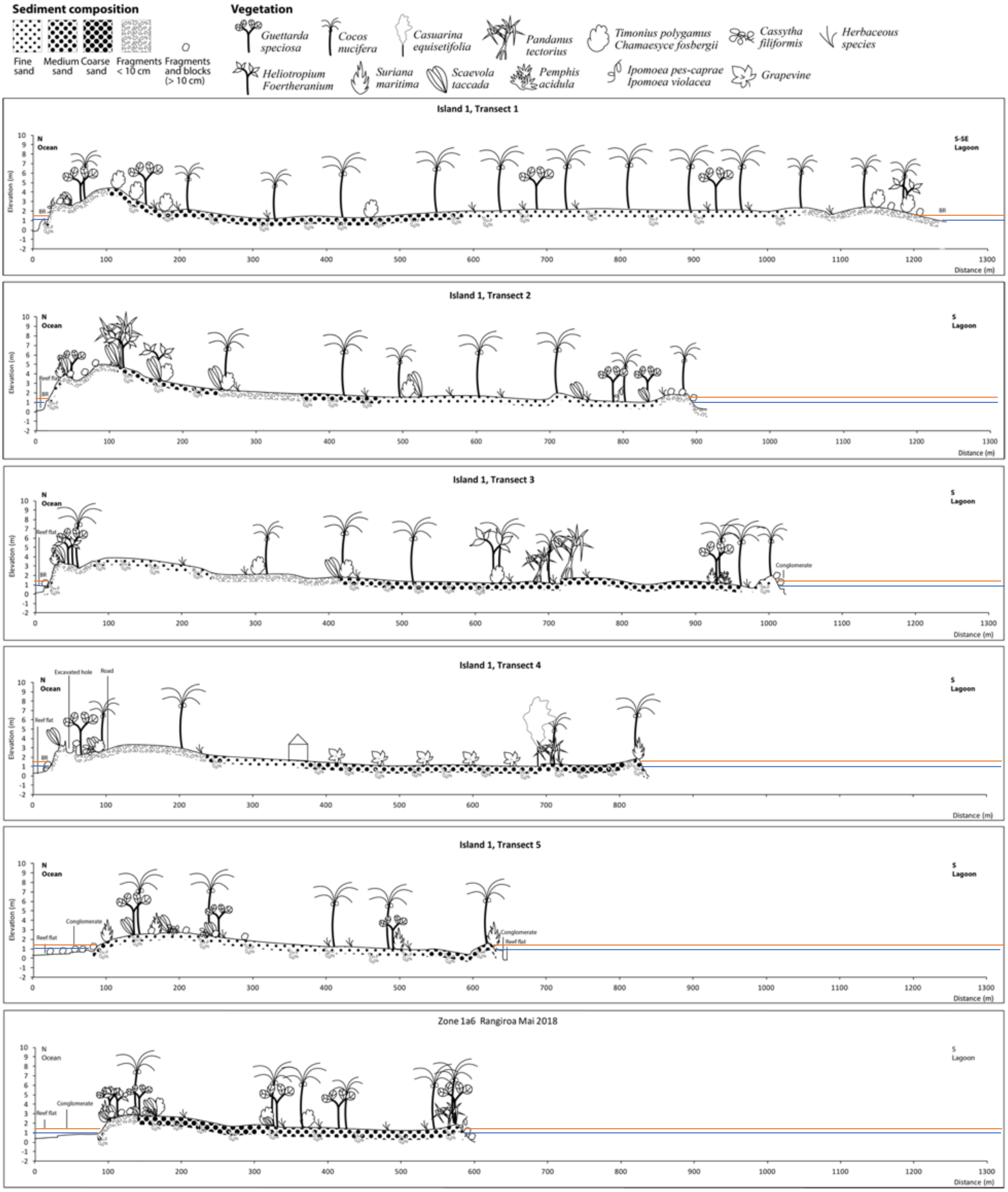

Island 2

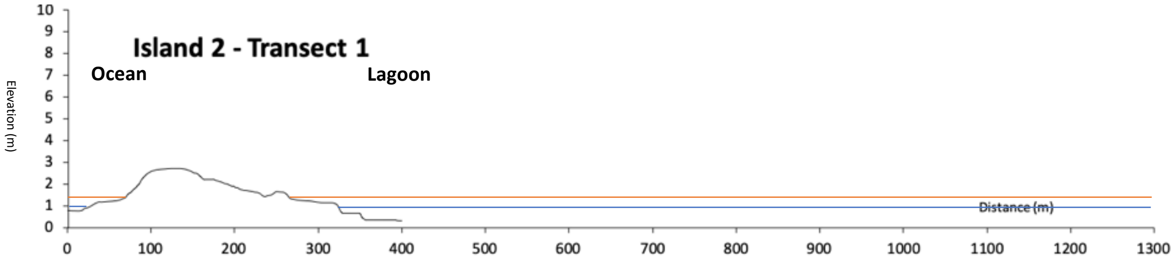

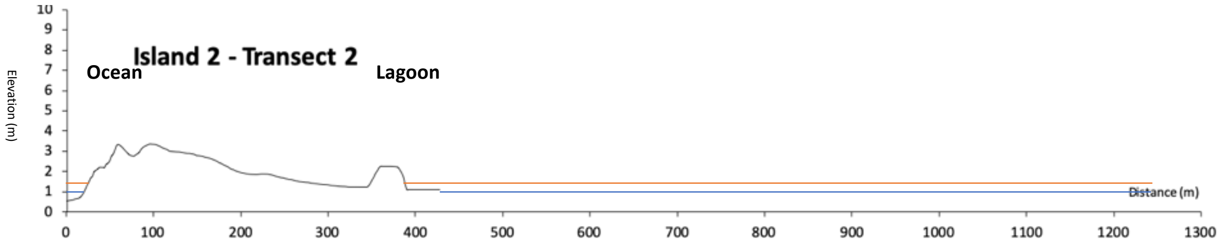

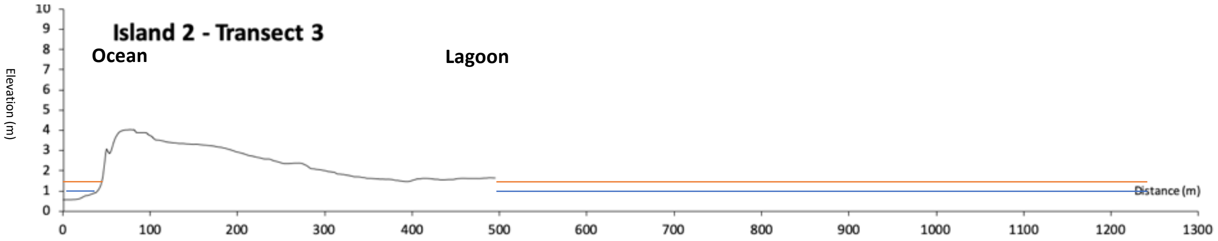

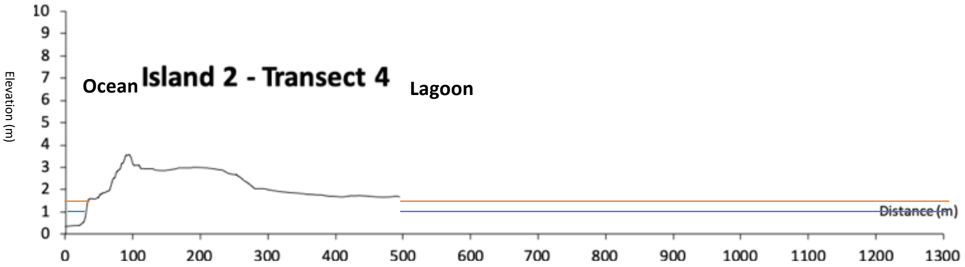

Island 3

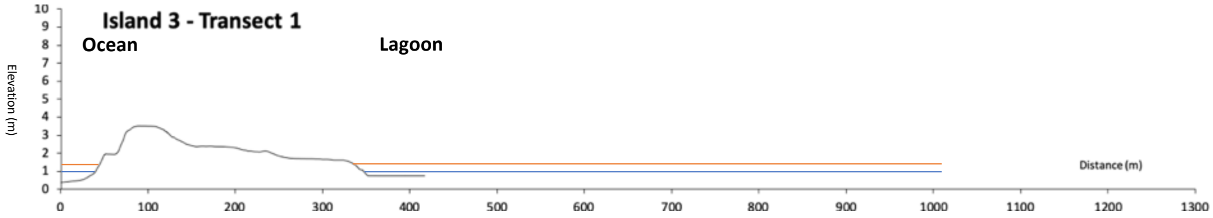

Island 4

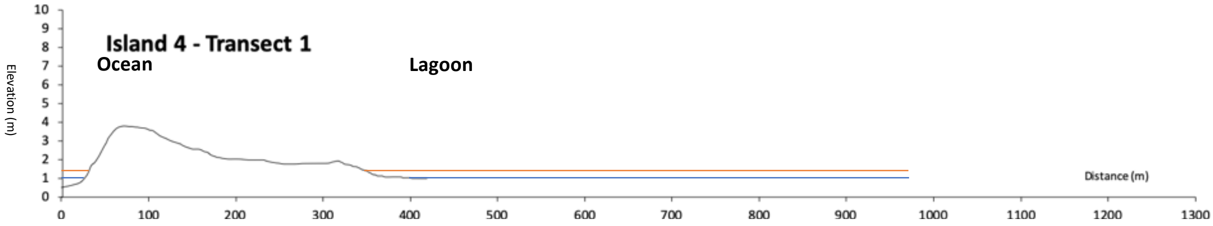

Island 5

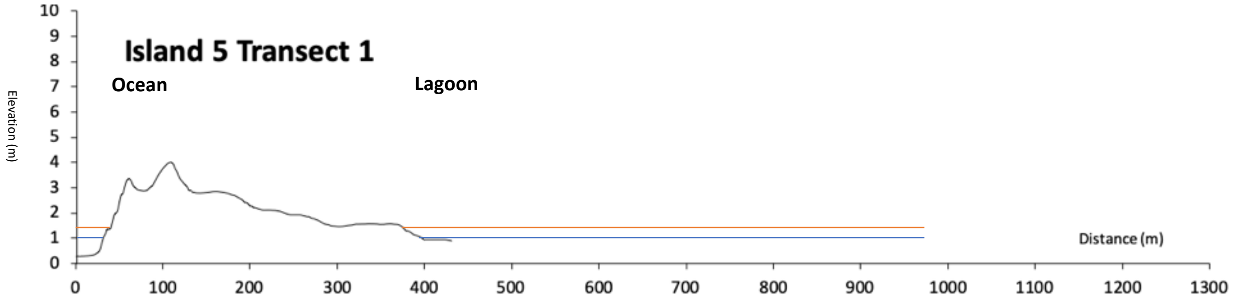
Island 5 Transect 1
Ocean
Lagoon
Elevation (m)
Distance (m)
0 1 2 3 4 5 6 7 8 9 10
0 100 200 300 400 500 600 700 800 900 1000 1100 1200 1300

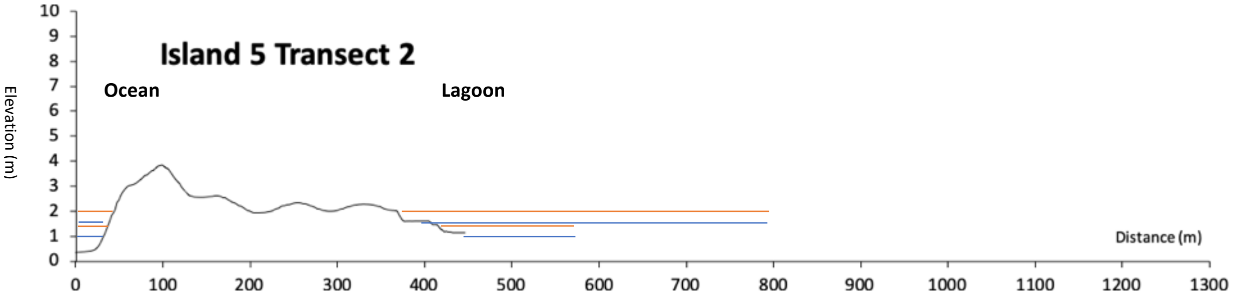
Island 5 Transect 2
Ocean
Lagoon
Elevation (m)
Distance (m)
0 1 2 3 4 5 6 7 8 9 10
0 100 200 300 400 500 600 700 800 900 1000 1100 1200 1300

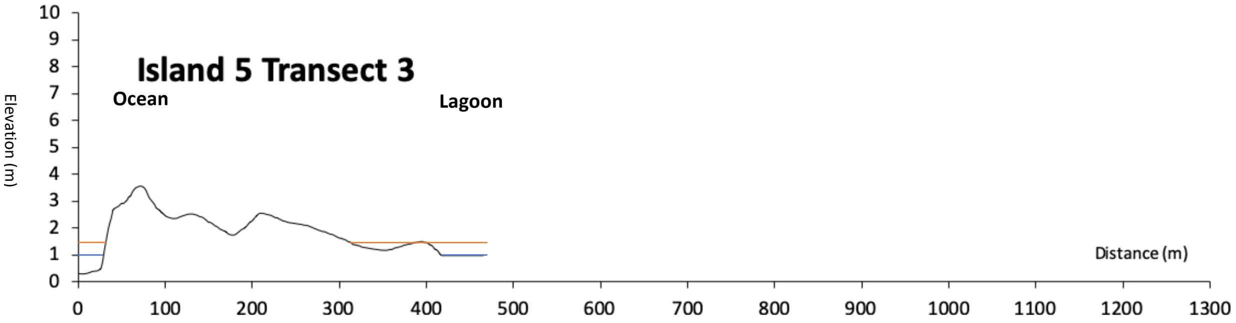
Island 5 Transect 3
Ocean
Lagoon
Elevation (m)
Distance (m)
0 1 2 3 4 5 6 7 8 9 10
0 100 200 300 400 500 600 700 800 900 1000 1100 1200 1300

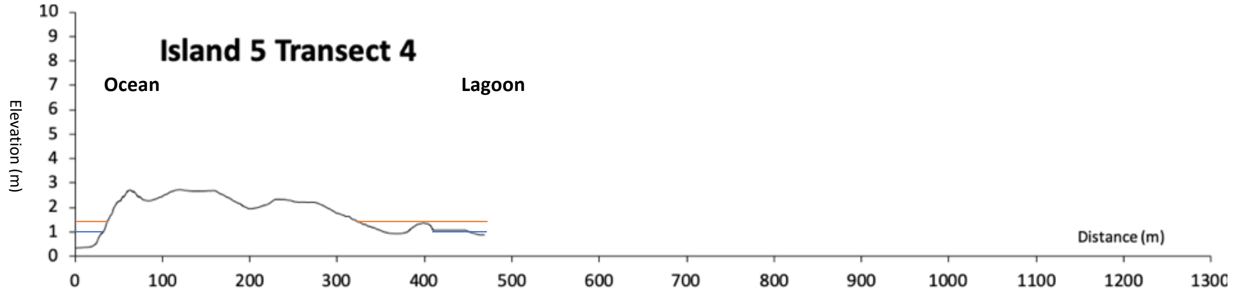
Island 5 Transect 4
Ocean
Lagoon
Elevation (m)
Distance (m)
0 1 2 3 4 5 6 7 8 9 10
0 100 200 300 400 500 600 700 800 900 1000 1100 1200 1300

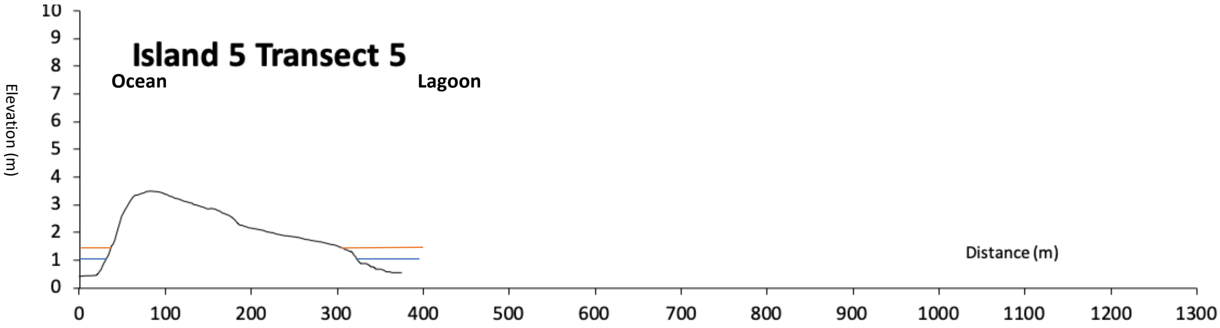
Island 5 Transect 5
Ocean
Lagoon
Elevation (m)
Distance (m)
0 1 2 3 4 5 6 7 8 9 10
0 100 200 300 400 500 600 700 800 900 1000 1100 1200 1300

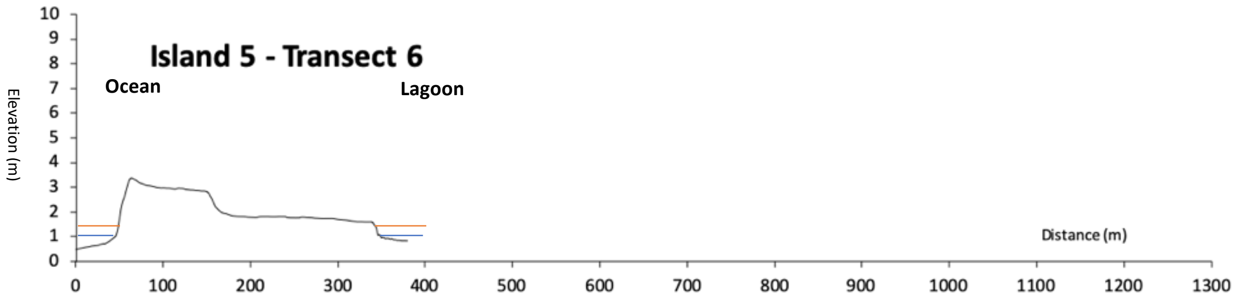

Island 6

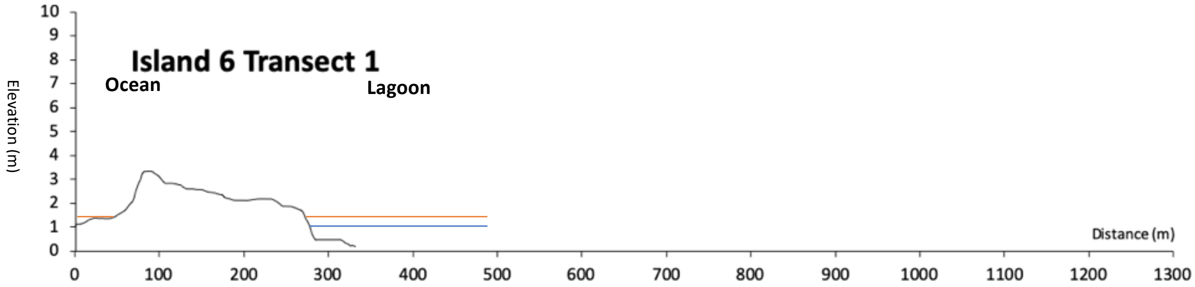

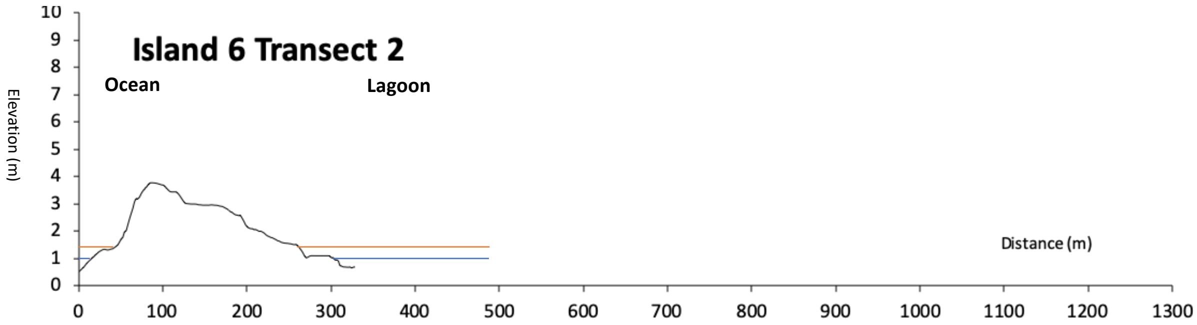

Island 7

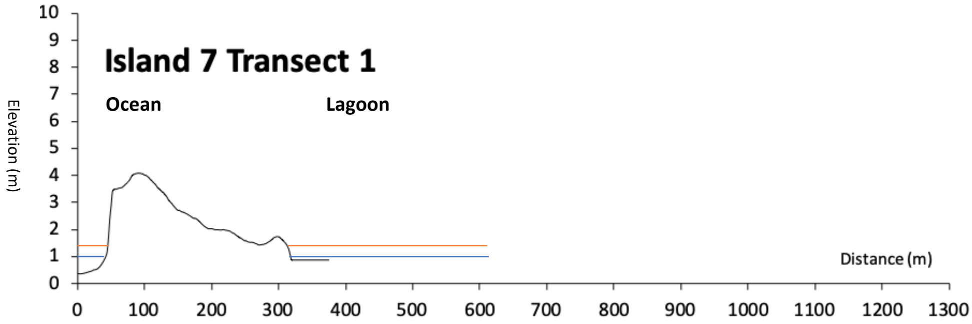

Island 8

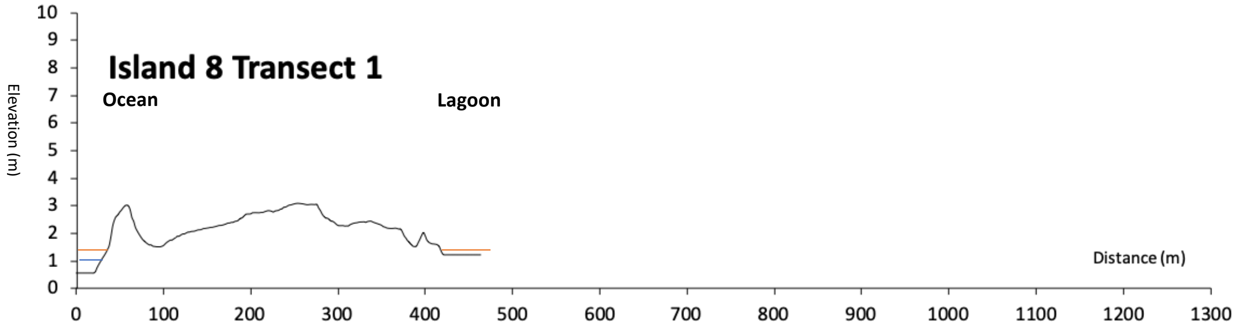
Island 8 Transect 1
Ocean
Lagoon
Elevation (m)
Distance (m)

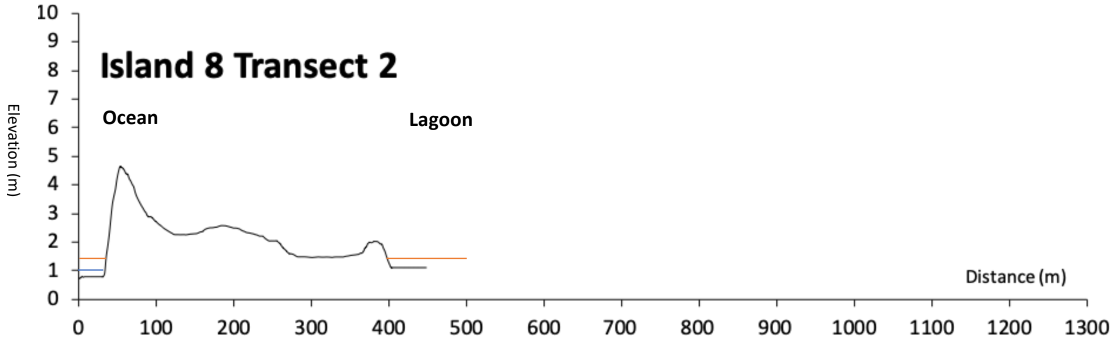
Island 8 Transect 2
Ocean
Lagoon
Elevation (m)
Distance (m)

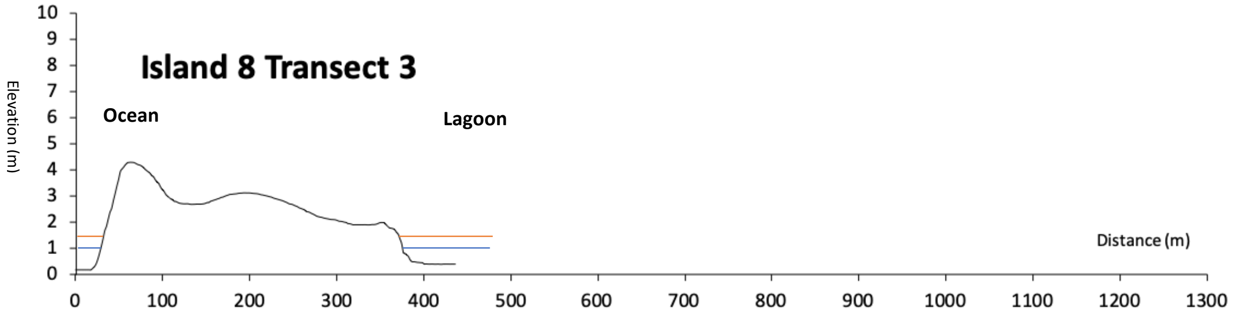
Island 8 Transect 3
Ocean
Lagoon
Elevation (m)
Distance (m)

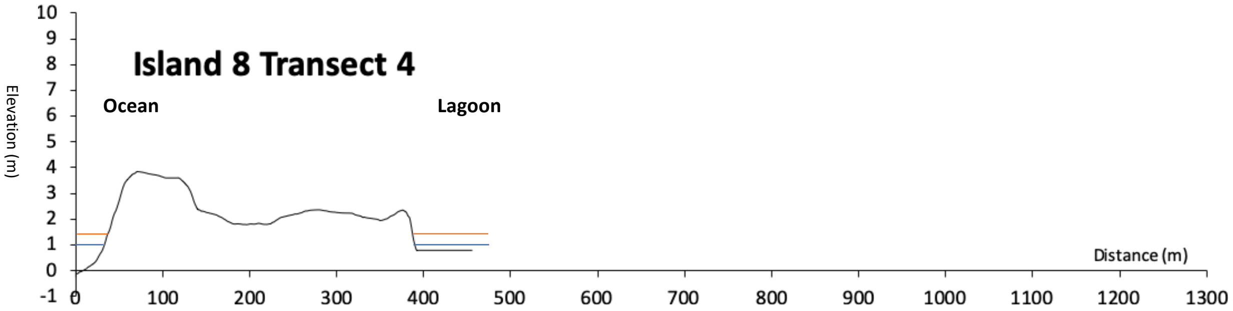
Island 8 Transect 4
Ocean
Lagoon
Elevation (m)
Distance (m)

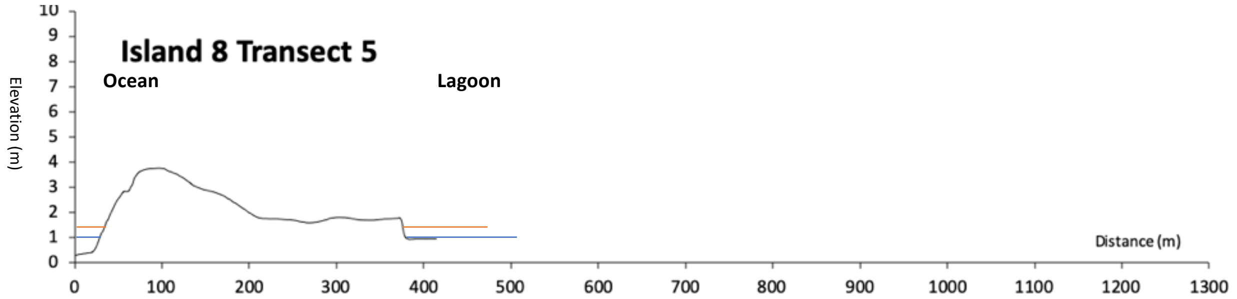
Island 8 Transect 5
Ocean
Lagoon
Elevation (m)
Distance (m)

Island 9

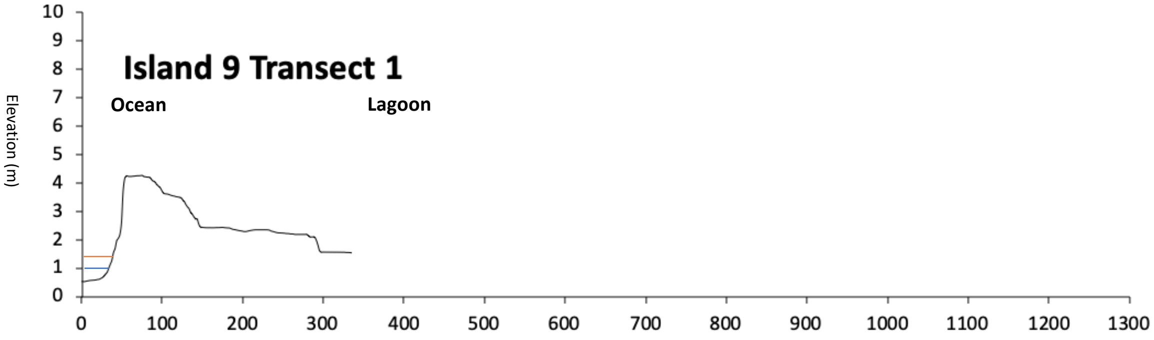
Island 9 Transect 1
Ocean
Lagoon
Elevation (m)
0 1 2 3 4 5 6 7 8 9 10
0 100 200 300 400 500 600 700 800 900 1000 1100 1200 1300

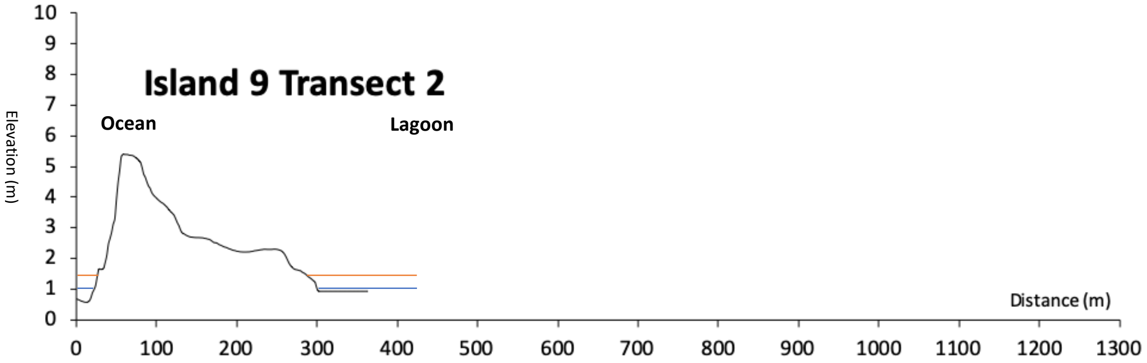
Island 9 Transect 2
Ocean
Lagoon
Elevation (m)
Distance (m)
0 1 2 3 4 5 6 7 8 9 10
0 100 200 300 400 500 600 700 800 900 1000 1100 1200 1300

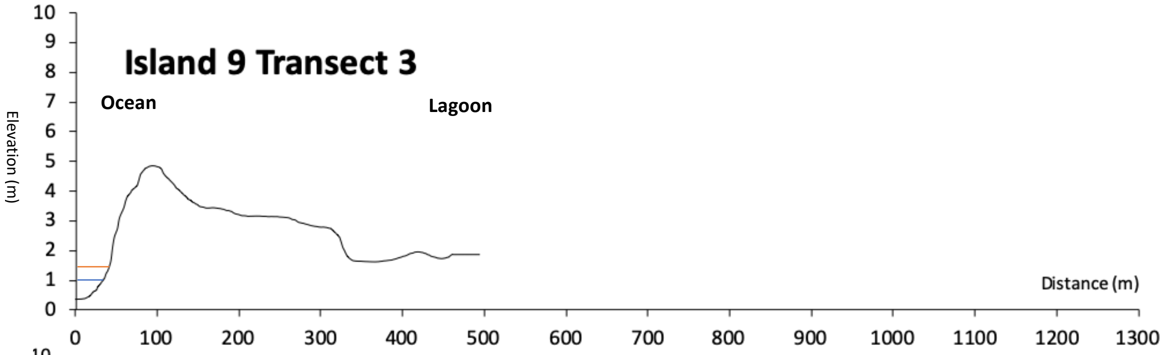
Island 9 Transect 3
Ocean
Lagoon
Elevation (m)
Distance (m)
0 1 2 3 4 5 6 7 8 9 10
0 100 200 300 400 500 600 700 800 900 1000 1100 1200 1300

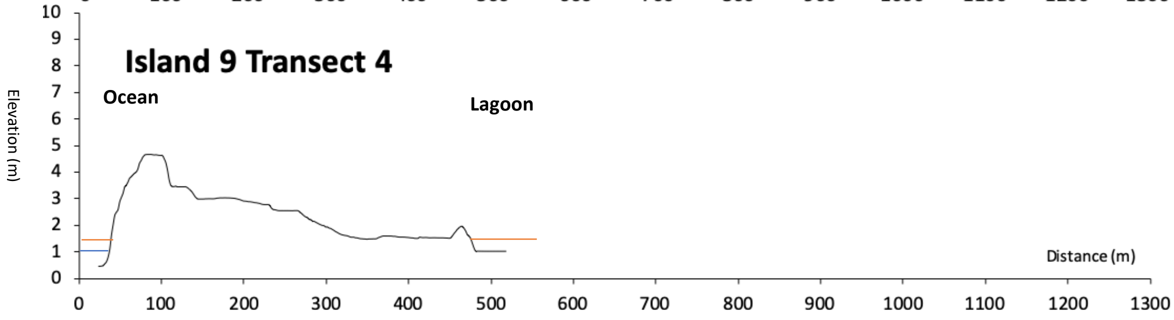
Island 9 Transect 4
Ocean
Lagoon
Elevation (m)
Distance (m)
0 1 2 3 4 5 6 7 8 9 10
0 100 200 300 400 500 600 700 800 900 1000 1100 1200 1300

Island 10

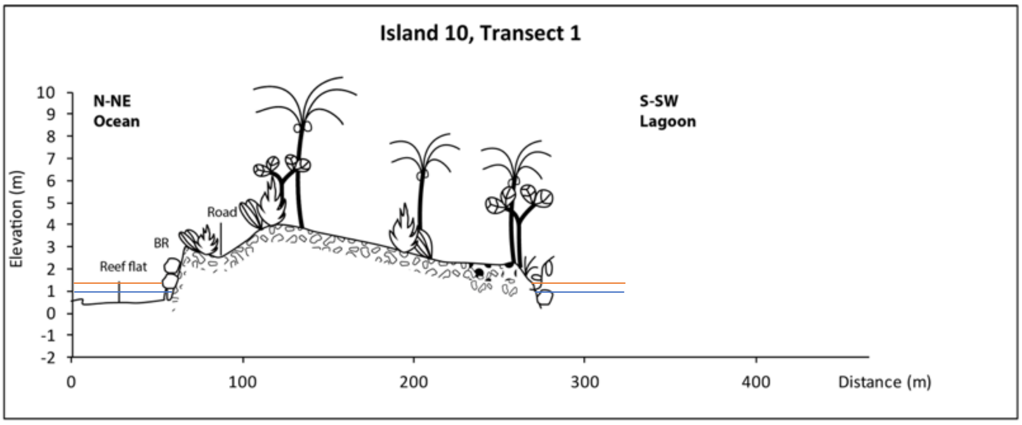
Island 10, Transect 1
N-NE
Ocean
S-SW
Lagoon
Elevation (m)
Distance (m)
Reef flat
BR
Road
10
9
8
7
6
5
4
3
2
1
0
-1
-2
0
100
200
300
400

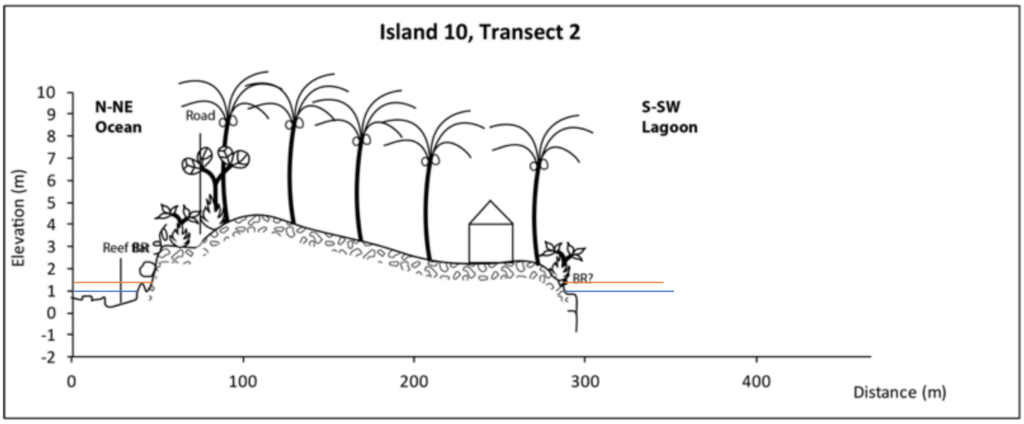
Island 10, Transect 2
N-NE
Ocean
S-SW
Lagoon
Elevation (m)
Distance (m)
Road
Reef flat
BR
BR?
10
9
8
7
6
5
4
3
2
1
0
-1
-2
0
100
200
300
400

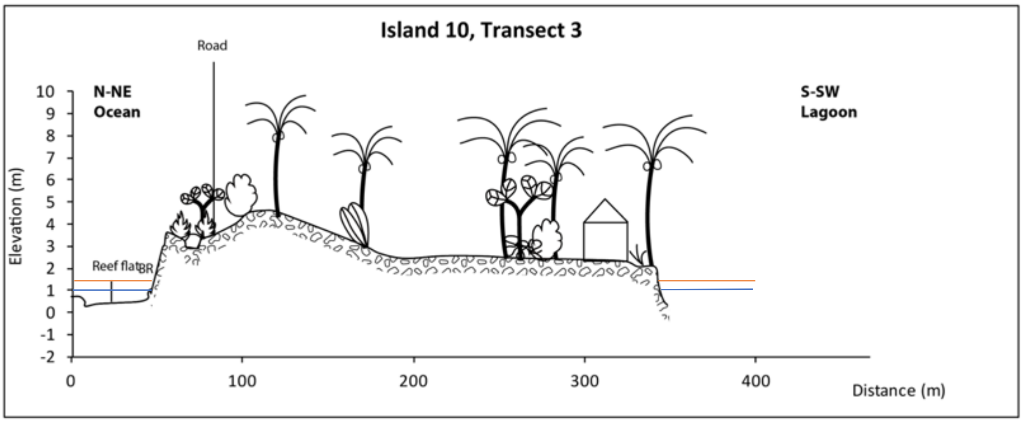
Island 10, Transect 3
Road
N-NE
Ocean
S-SW
Lagoon
Elevation (m)
Distance (m)
Reef flat
BR
10
9
8
7
6
5
4
3
2
1
0
-1
-2
0
100
200
300
400

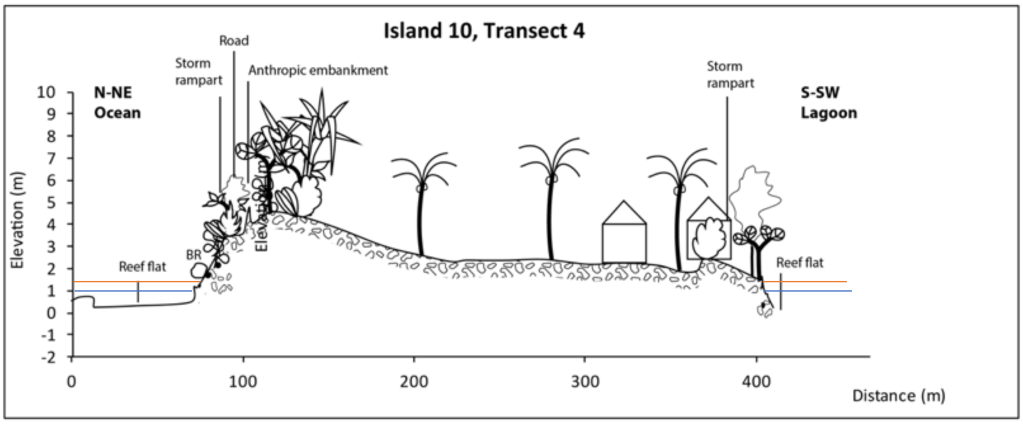
Island 10, Transect 4
Road
Storm
rampart
Anthropic embankment
Storm
rampart
N-NE
Ocean
S-SW
Lagoon
Elevation (m)
Distance (m)
BR
Reef flat
Reef flat
10
9
8
7
6
5
4
3
2
1
0
-1
-2
0
100
200
300
400

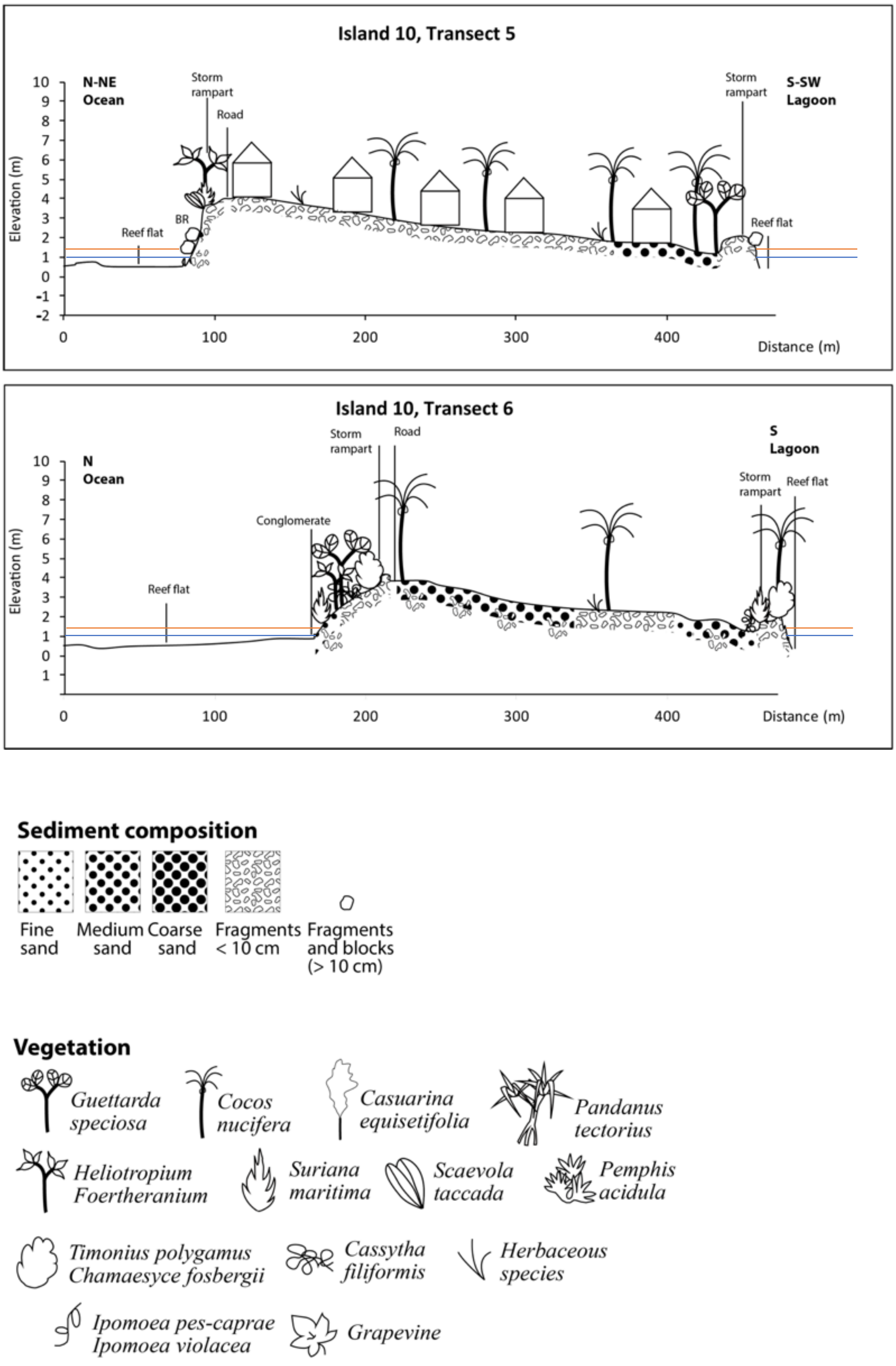
Island 10, Transect 5
N-NE
Ocean
Storm rampart
Road
BR
Reef flat
Storm rampart
S-SW
Lagoon
Reef flat
Elevation (m)
Distance (m)
Island 10, Transect 6
N
Ocean
Reef flat
Conglomerate
Storm rampart
Road
S
Lagoon
Storm rampart
Reef flat
Elevation (m)
Distance (m)
Sediment composition
Fine sand
Medium sand
Coarse sand
Fragments < 10 cm
Fragments and blocks (> 10 cm)
Vegetation
Guettarda speciosa
Cocos nucifera
Casuarina equisetifolia
Pandanus tectorius
Heliotropium Foertheranium
Suriana maritima
Scaevola taccada
Pemphis acidula
Timonius polygamus
Chamaesyce fosbergii
Cassytha filiformis
Herbaceous species
Ipomoea pes-caprae
Ipomoea violacea
Grapevine

Island 11

Island 11, Transect 1

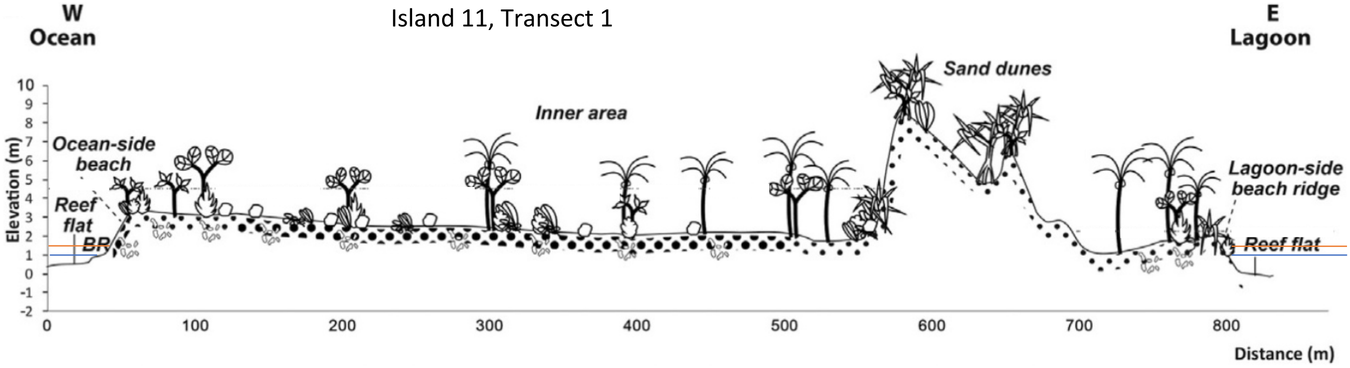

Island 11, Transect 2

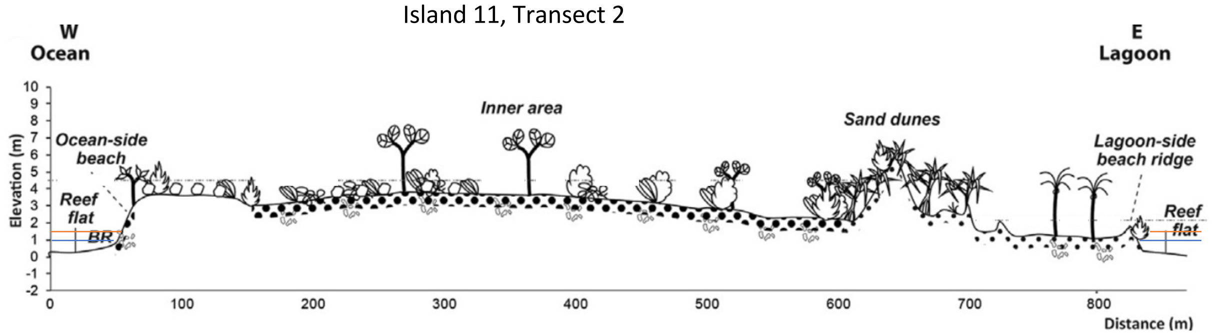

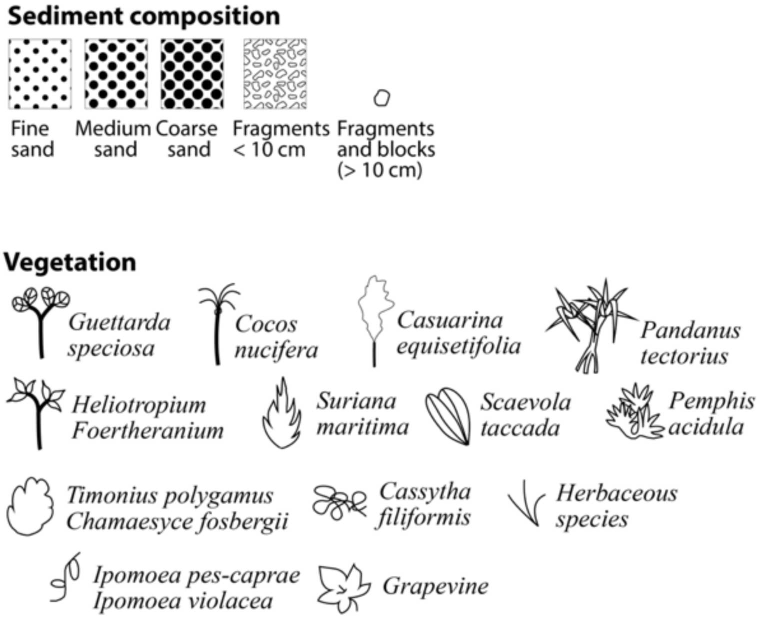

Island 12

Island 12, Transect 1

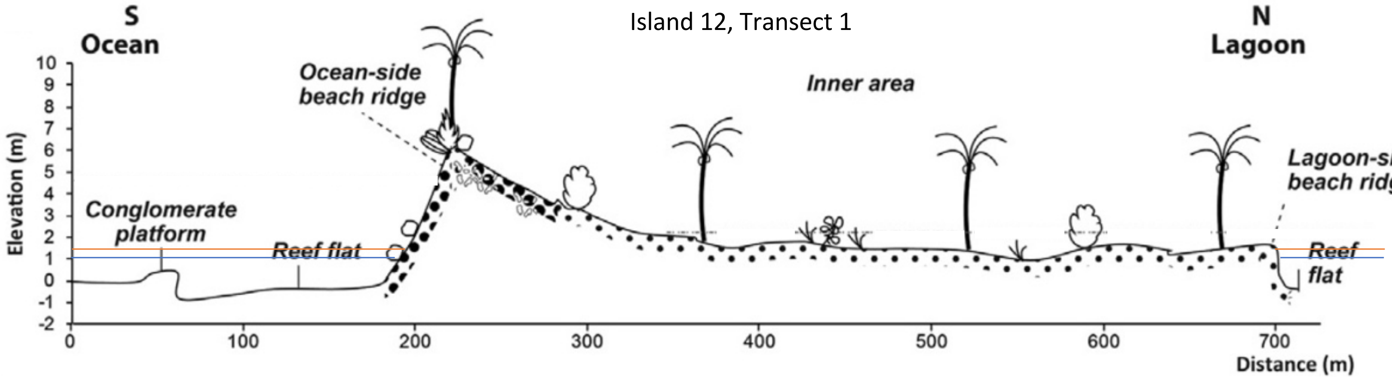

Island 12, Transect 2

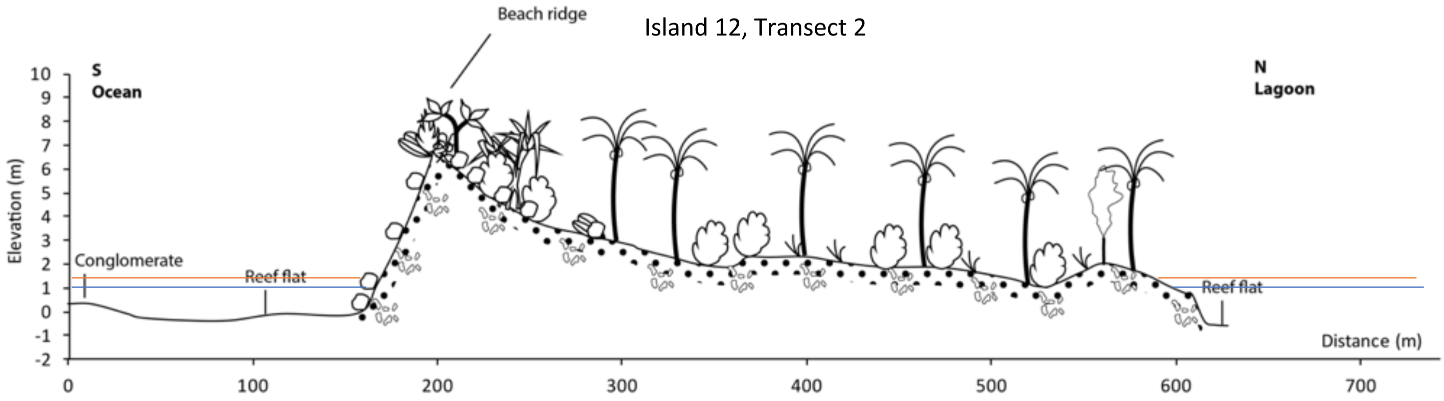

Island 12, Transect 3

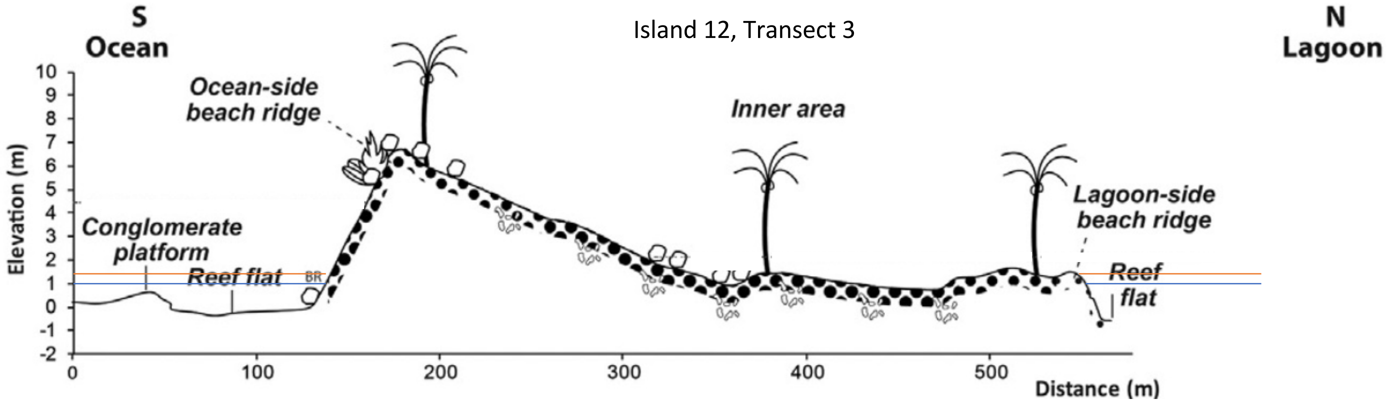

Island 12, Transect 4

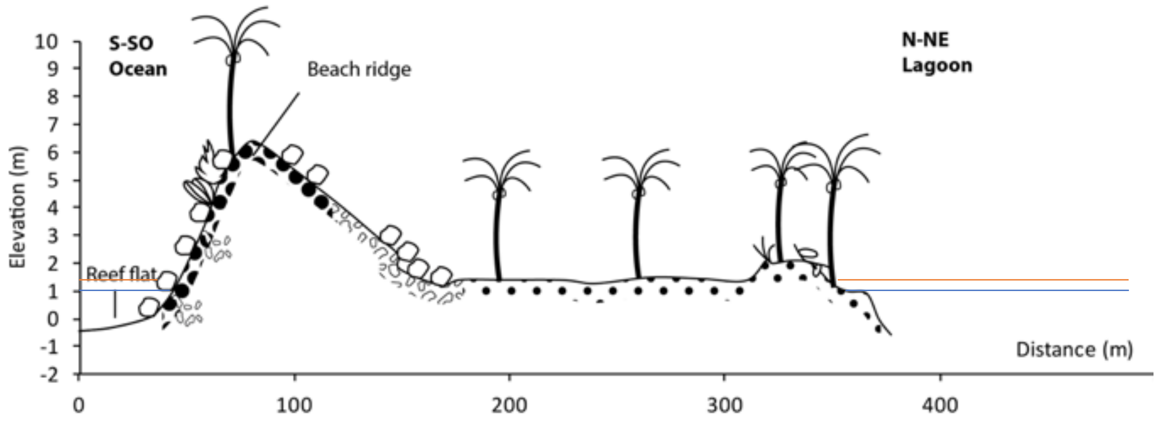

Island 12, Transect 5

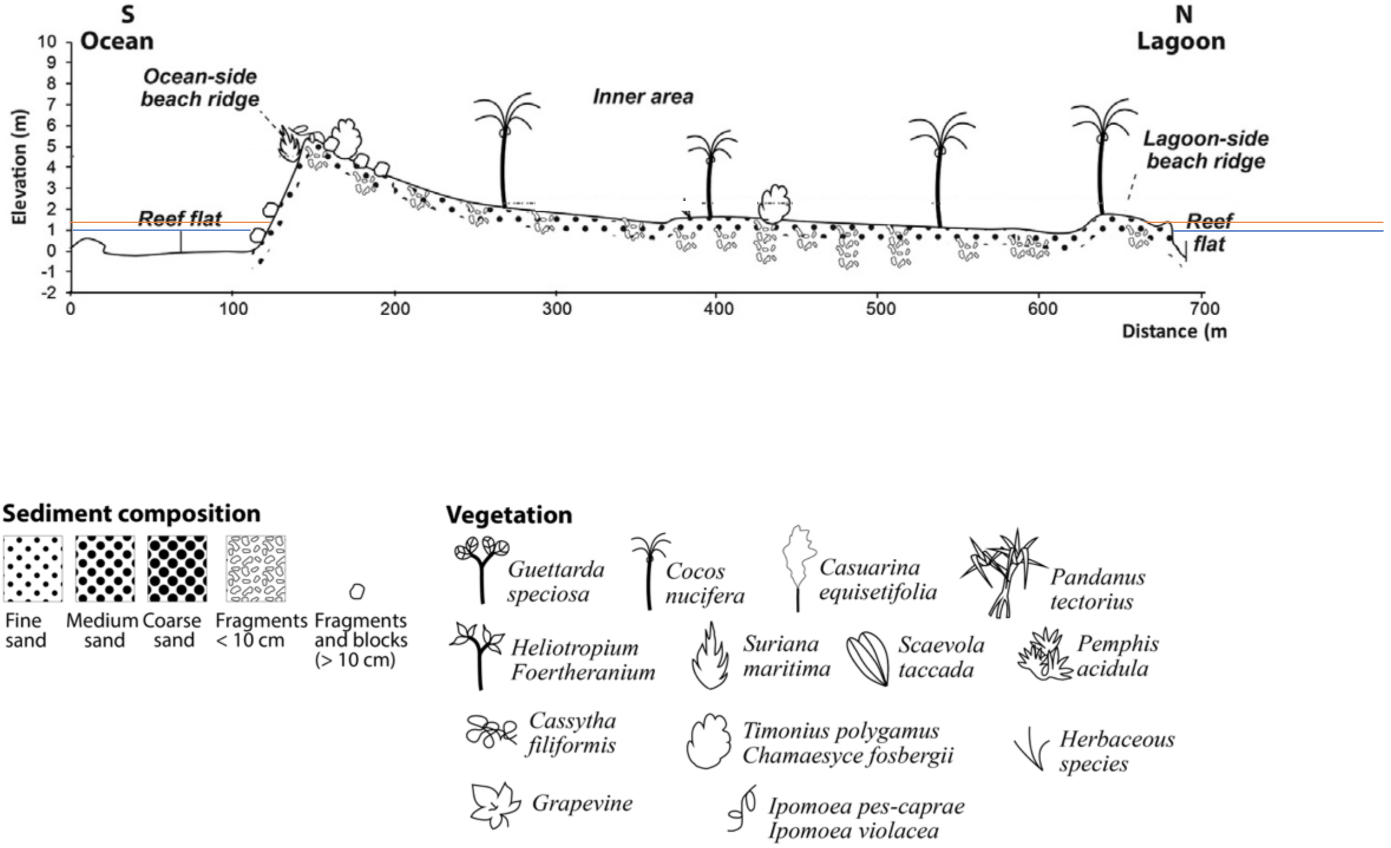

Supplementary Material 6. Cyclone-driven extreme flood scenarios under SSP1-2.6 and SSP5-8.5 in 2100.

Maximum instantaneous water levels were estimated using the data provided by two numerical modeling studies revealing ESLs of respectively 4.50 m and 1.70 m above hydrographic zero on the ocean (Pedreros et al., 2010) and lagoon (Damlamian and Kruger, 2013) coasts of Avatoru Island. These ESLs are superimposed on highest spring tide (HST; +0.60m) and 0.45m of SLR for SSP1-2.6 and 0.78m of SLR for SSP5-8.5. Blue dotted line: current instantaneous water level along lagoon coast; Blue line: current instantaneous water level along ocean coast; Thin orange line: instantaneous water level along ocean (5.55 m) and lagoon (2.75 m) coasts under SSP1-2.6 (+0.45 m); Thick orange line: instantaneous water level along ocean (5.88 m) and lagoon (3.08 m) coasts under SSP5-8.5 (+0.78 m).

The lines shown in the following figures indicate **maximum instantaneous water levels at the coast**. These water levels decrease landwards, which is not shown in the figures, because we did not run hydrodynamic flood modelling and are therefore not able to determine water levels in the inner part of islands. Therefore, inland water levels are over-estimated in these figures. The latter however exhibit marked differences between islands, which our approach aimed at highlighting to assess island potential for internal relocation.

Island 1

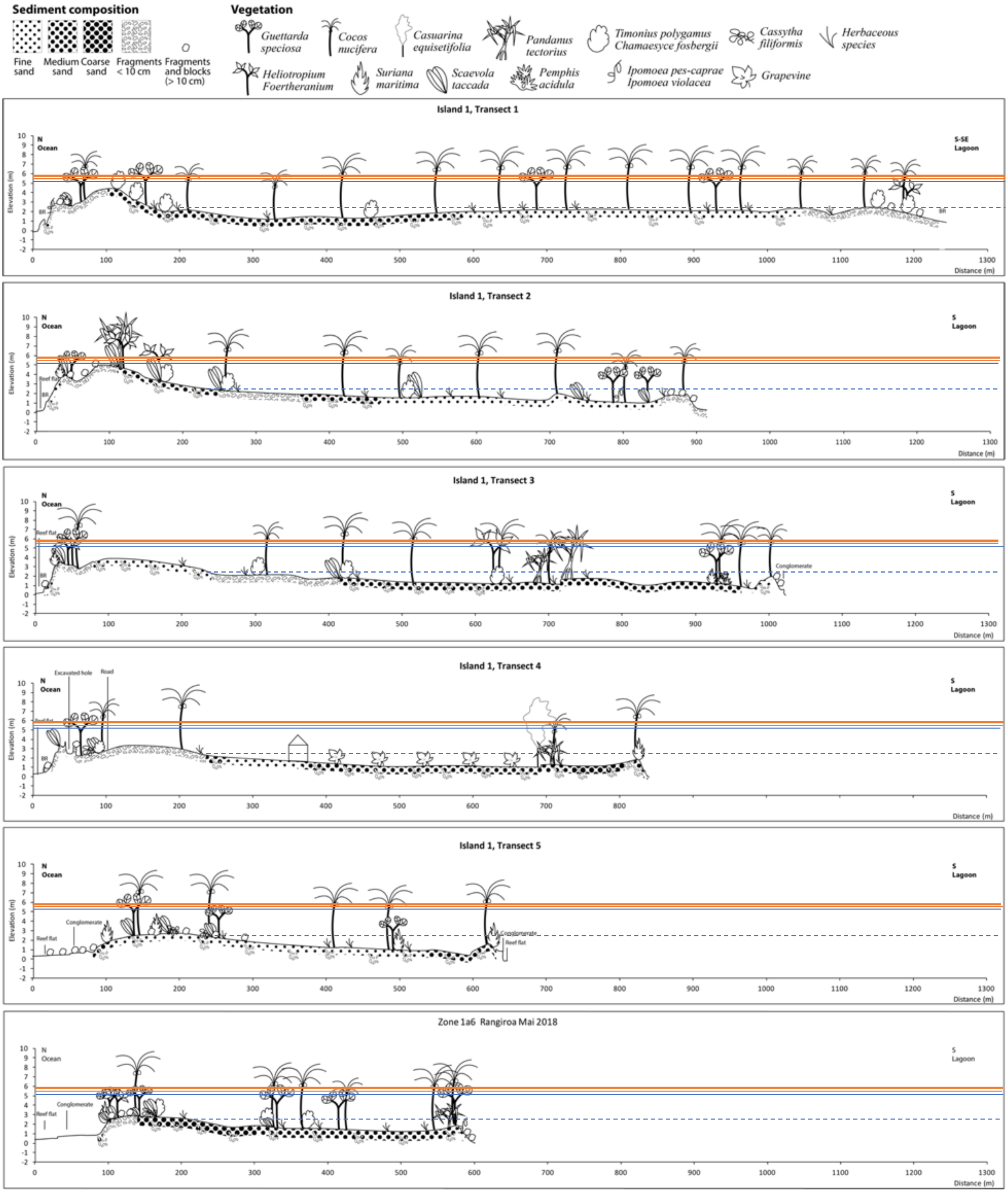

Island 2

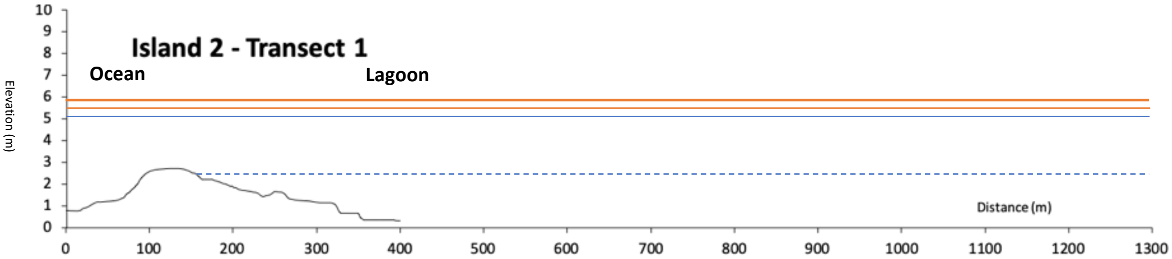

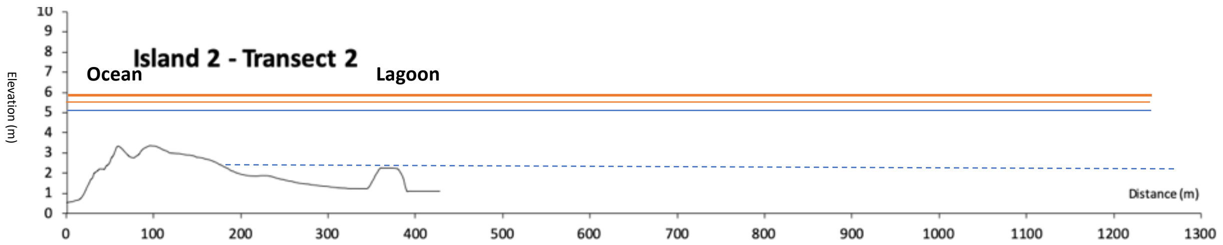

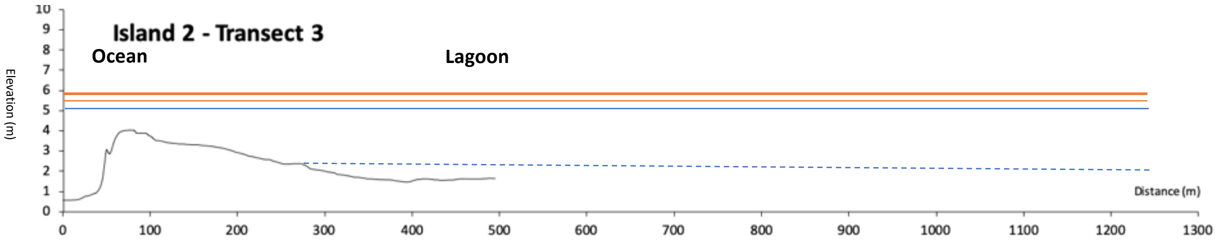

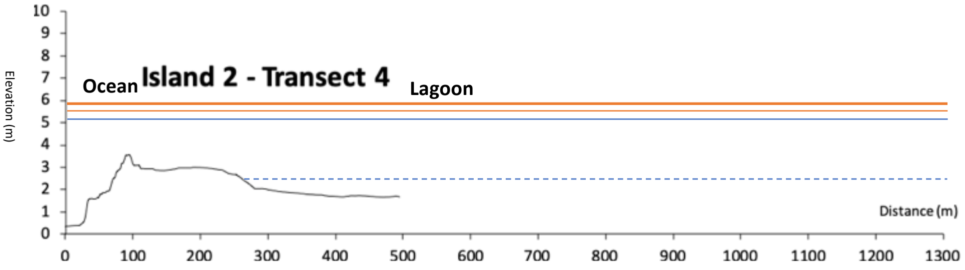

Island 3

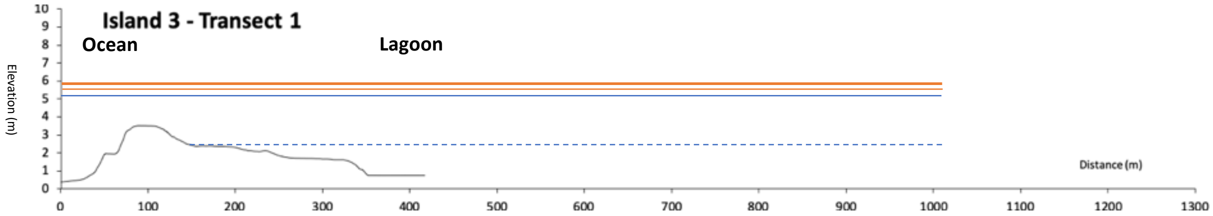

Island 4

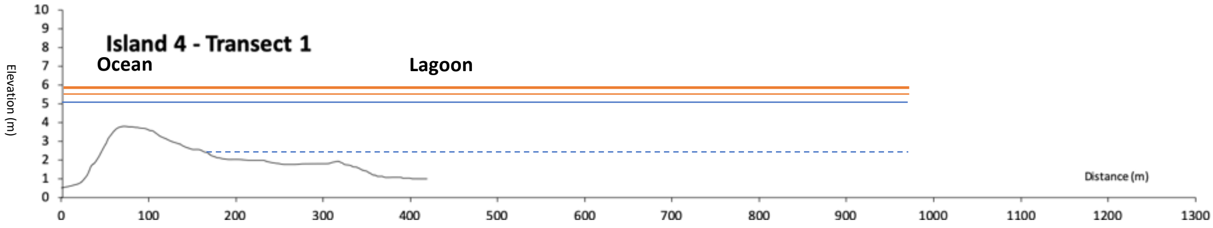

Island 5

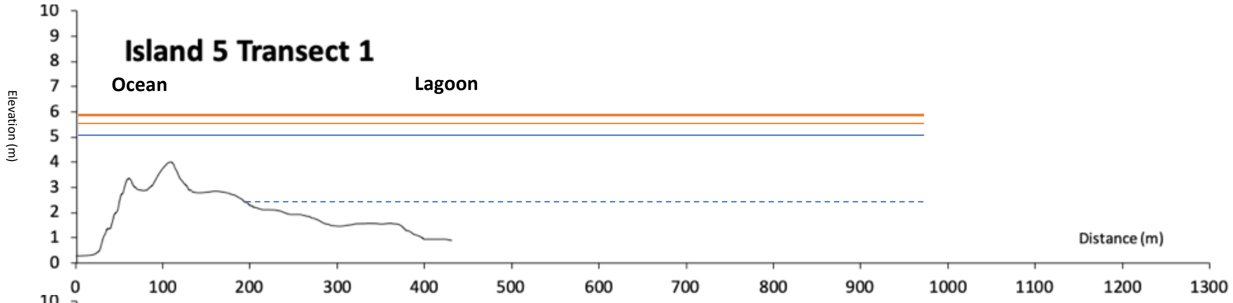
Island 5 Transect 1
Ocean
Lagoon
Elevation (m)
Distance (m)

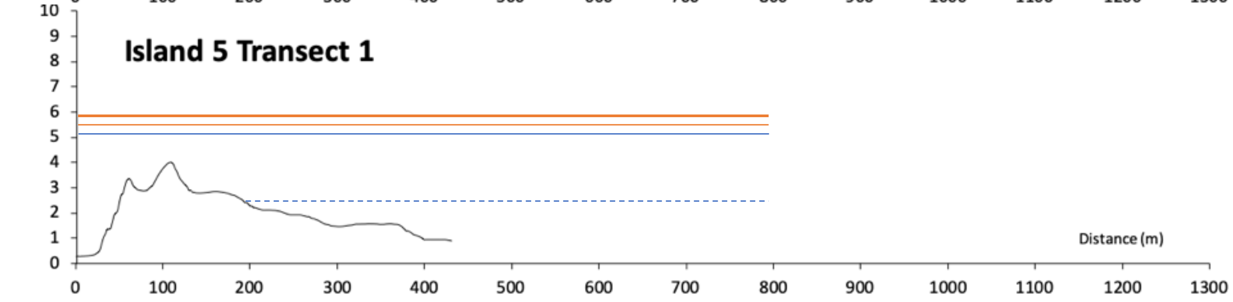
Island 5 Transect 1
Distance (m)

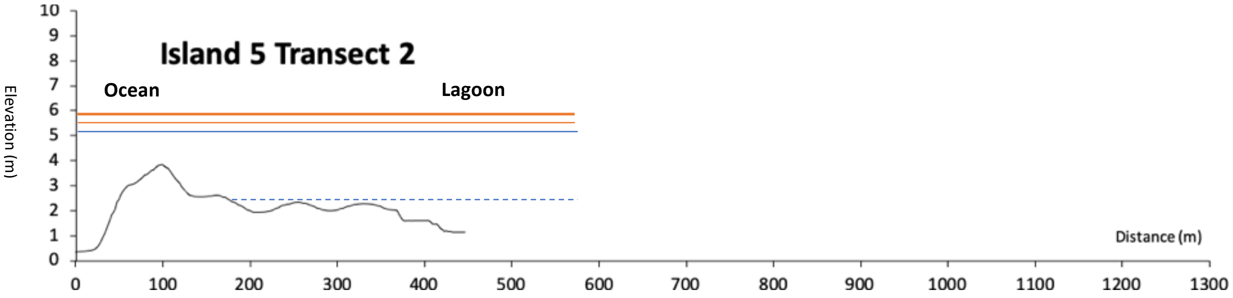
Island 5 Transect 2
Ocean
Lagoon
Elevation (m)
Distance (m)

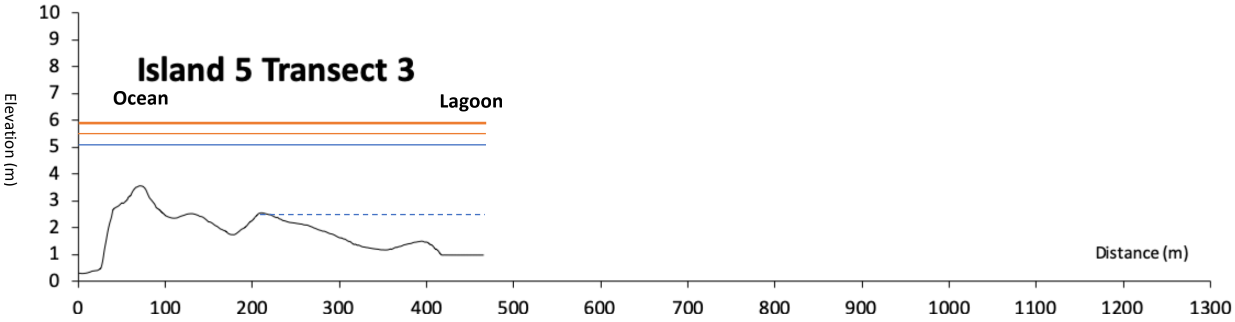
Island 5 Transect 3
Ocean
Lagoon
Elevation (m)
Distance (m)

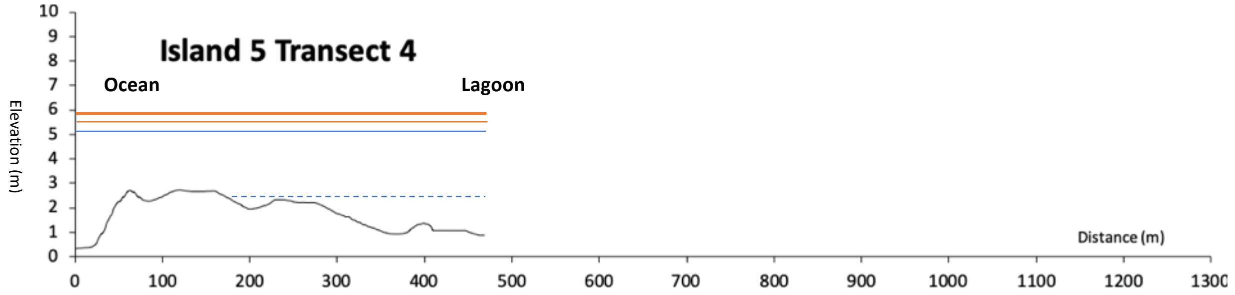
Island 5 Transect 4
Ocean
Lagoon
Elevation (m)
Distance (m)

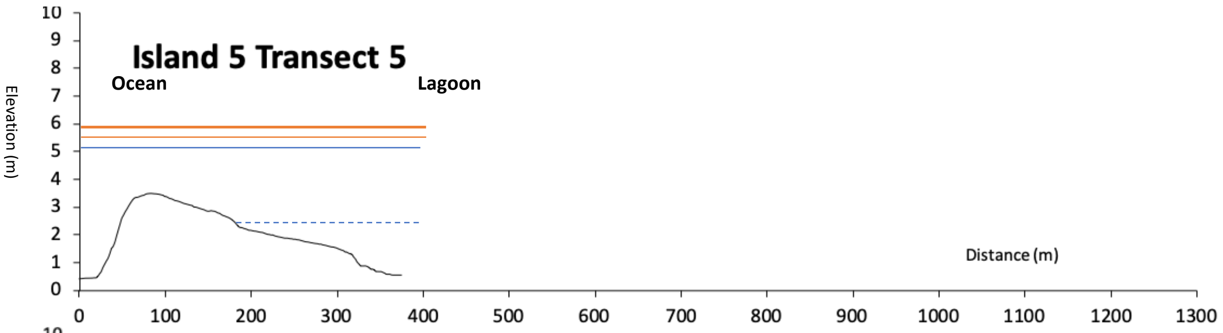

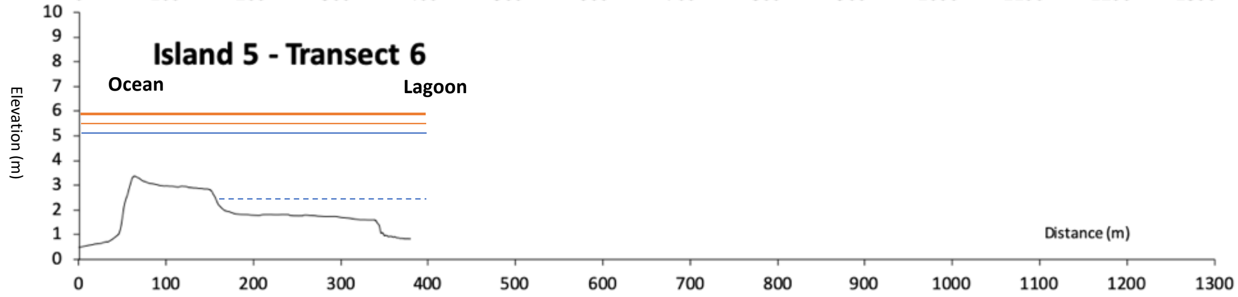

Island 6

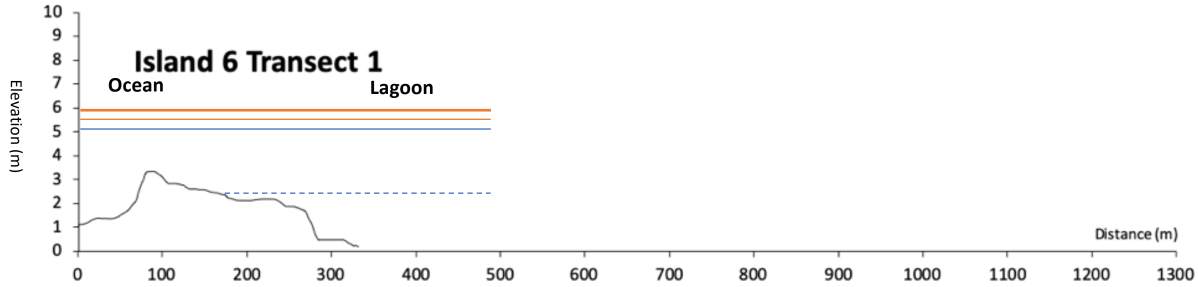

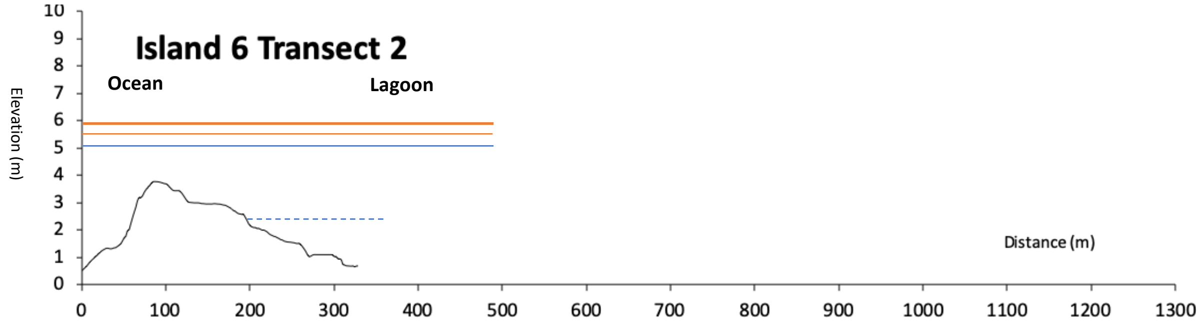

Island 7

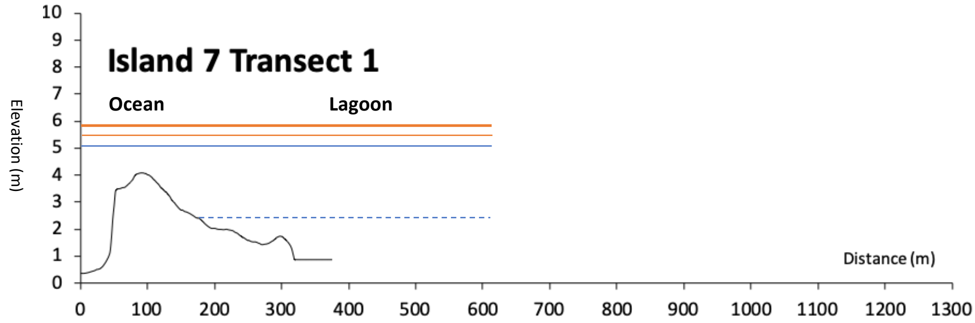

Island 8

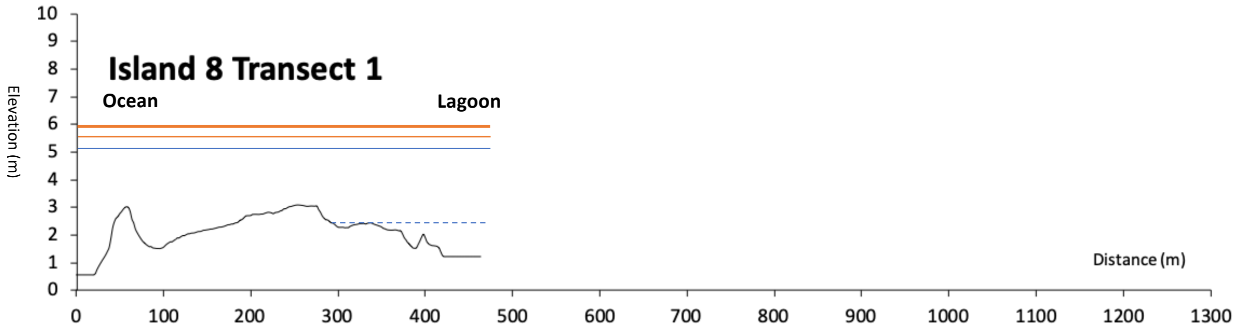
Island 8 Transect 1
Ocean
Lagoon
Elevation (m)
Distance (m)

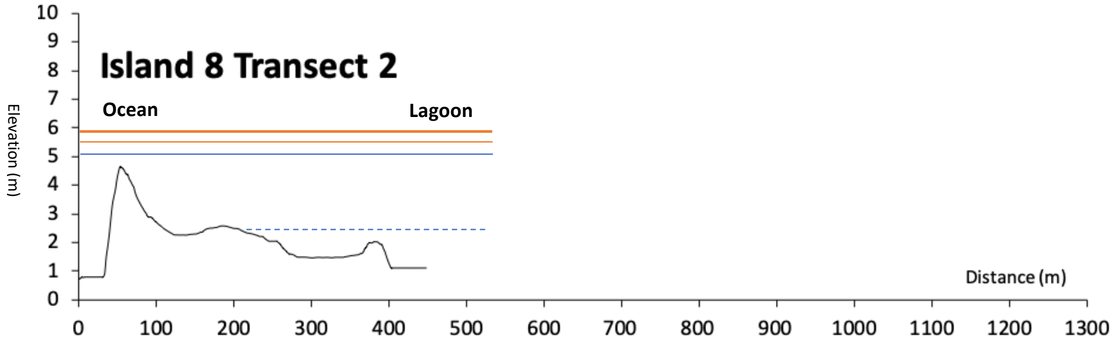
Island 8 Transect 2
Ocean
Lagoon
Elevation (m)
Distance (m)

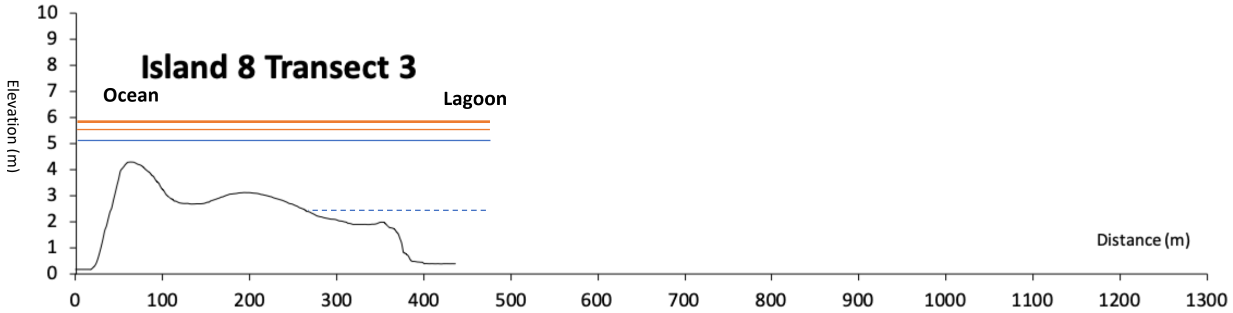
Island 8 Transect 3
Ocean
Lagoon
Elevation (m)
Distance (m)

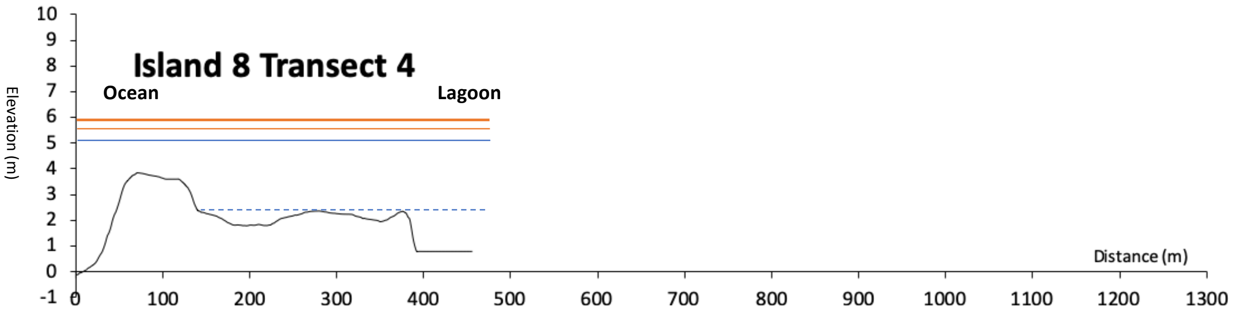
Island 8 Transect 4
Ocean
Lagoon
Elevation (m)
Distance (m)

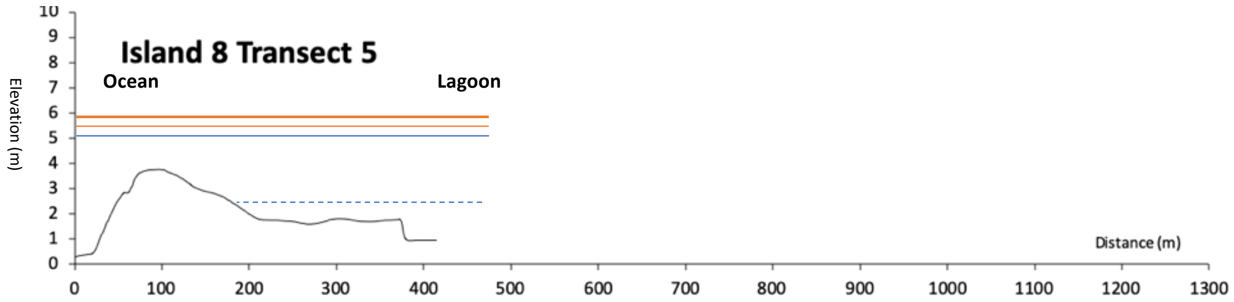
Island 8 Transect 5
Ocean
Lagoon
Elevation (m)
Distance (m)

Island 9

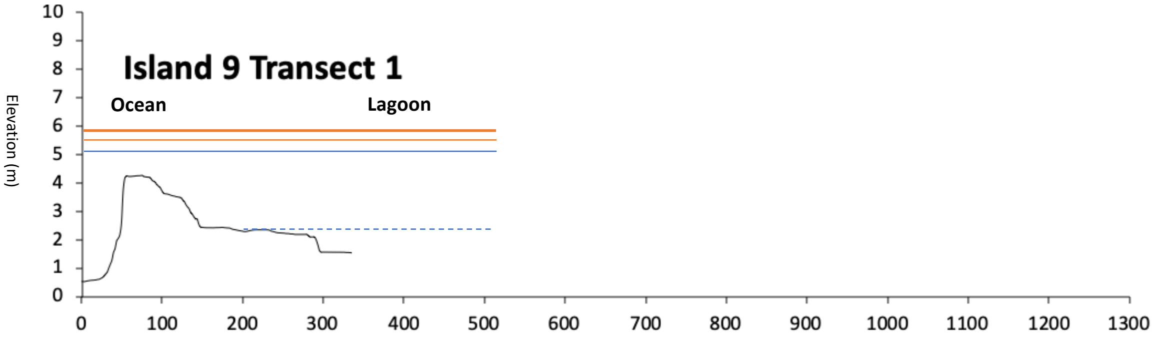
Island 9 Transect 1
Ocean
Lagoon
Elevation (m)

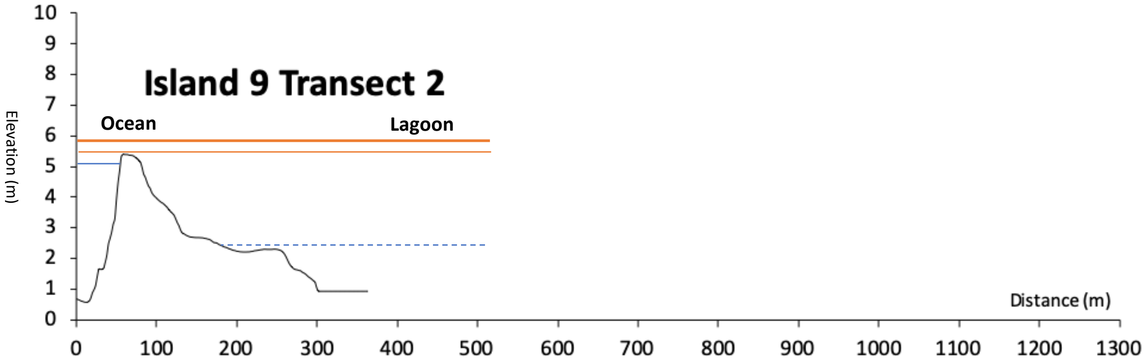
Island 9 Transect 2
Ocean
Lagoon
Elevation (m)
Distance (m)

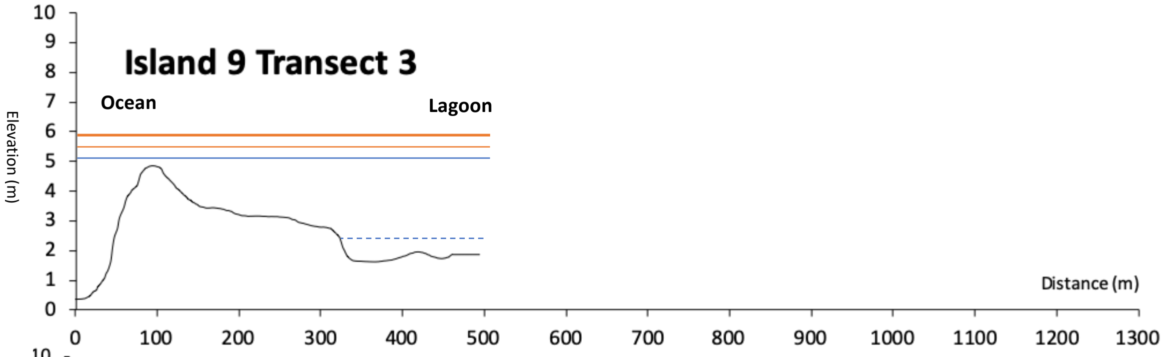
Island 9 Transect 3
Ocean
Lagoon
Elevation (m)
Distance (m)

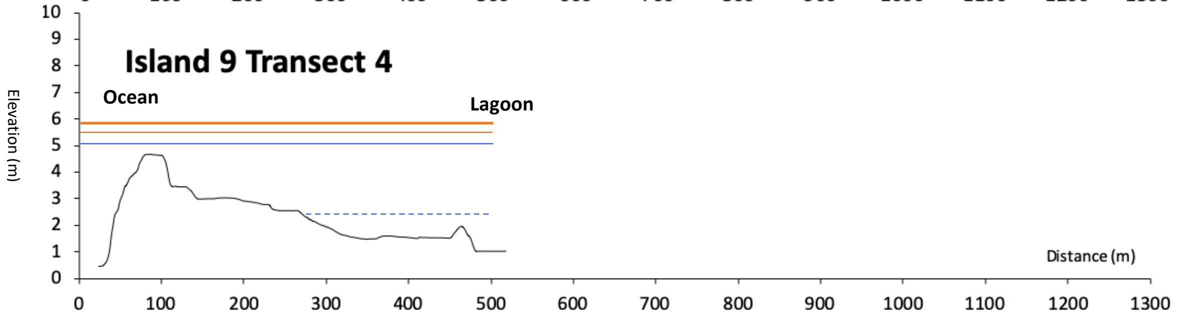
Island 9 Transect 4
Ocean
Lagoon
Elevation (m)
Distance (m)

Island 10

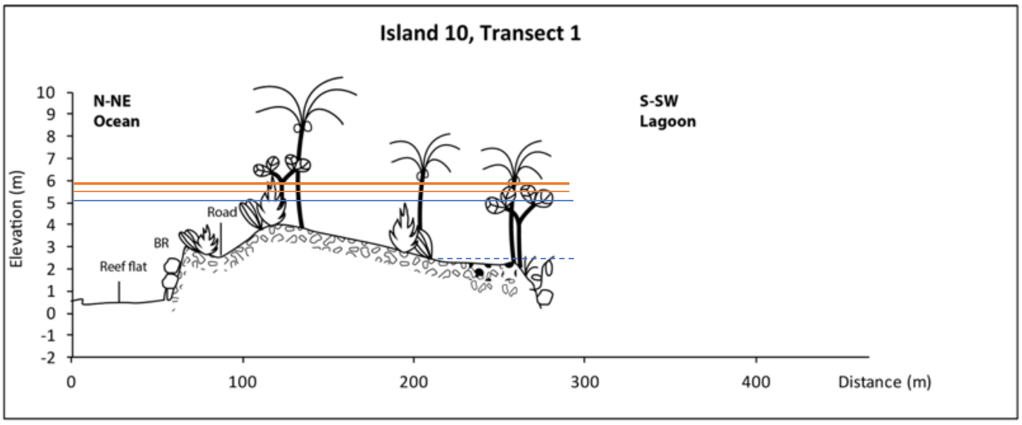

Island 10, Transect 1
N-NE
Ocean
S-SW
Lagoon
Road
BR
Reef flat
Elevation (m)
Distance (m)

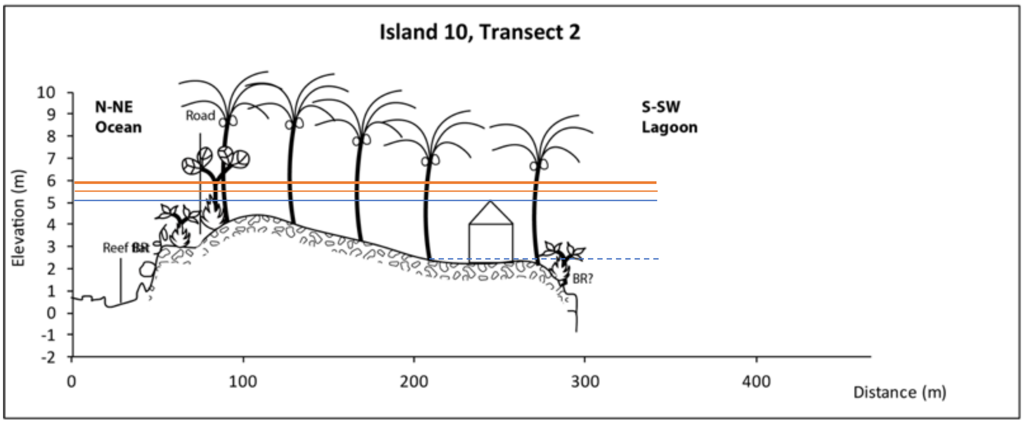

Island 10, Transect 2
N-NE
Ocean
S-SW
Lagoon
Road
Reef flat
BR
BR?
Elevation (m)
Distance (m)

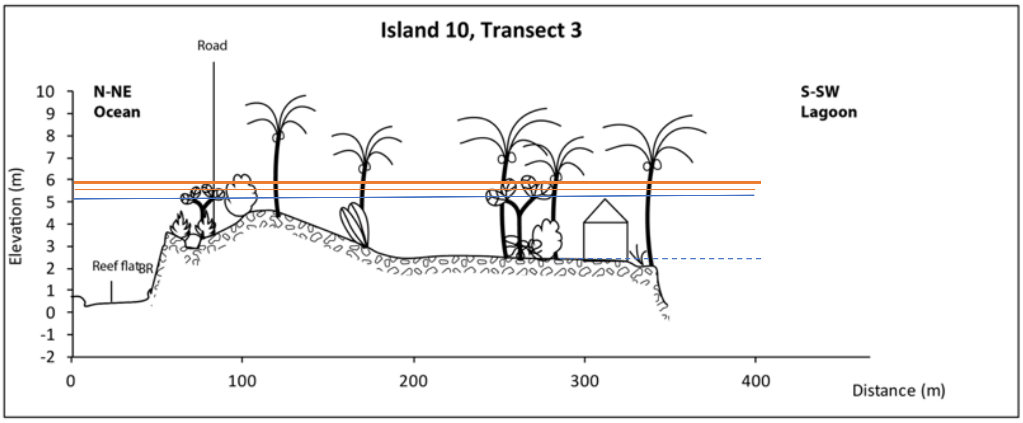

Island 10, Transect 3
Road
N-NE
Ocean
S-SW
Lagoon
Reef flat
BR
Elevation (m)
Distance (m)

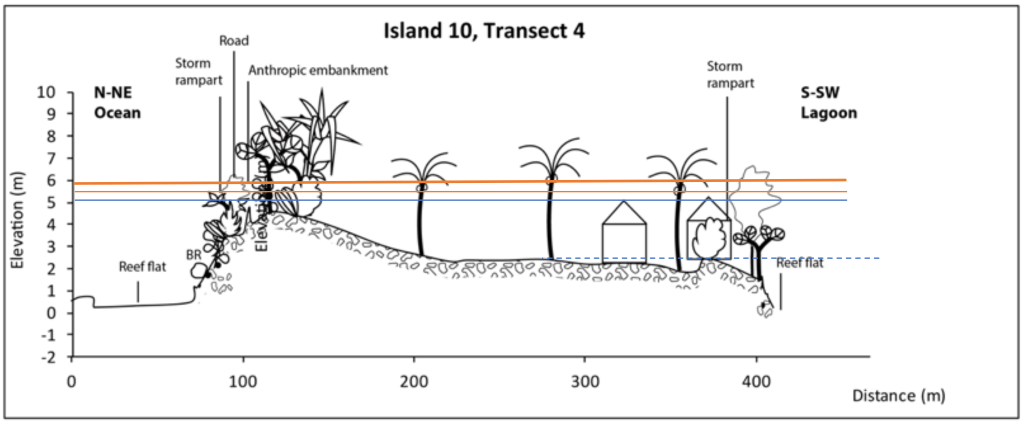

Island 10, Transect 4
Road
Storm rampart
Anthropic embankment
Storm rampart
N-NE
Ocean
S-SW
Lagoon
BR
Reef flat
Reef flat
Elevation (m)
Distance (m)

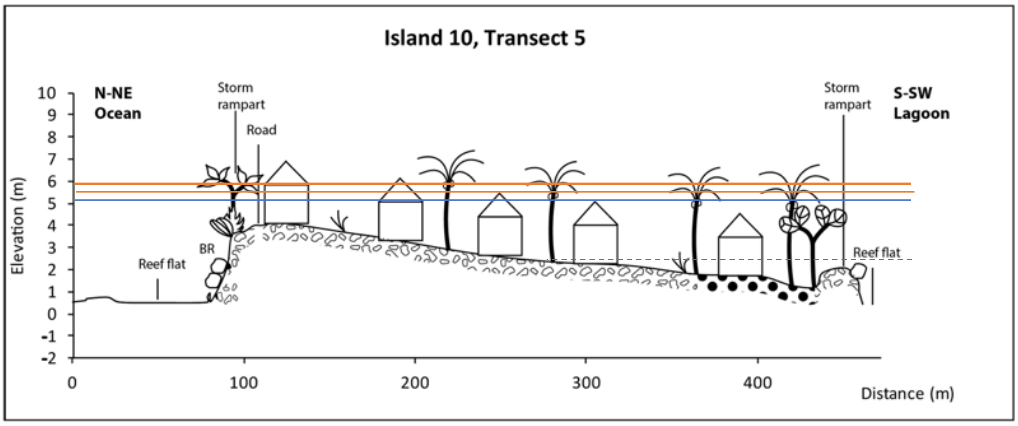
Island 10, Transect 5
N-NE
Ocean
Storm rampart
Road
BR
Reef flat
Storm rampart
S-SW
Lagoon
Reef flat
Elevation (m)
Distance (m)

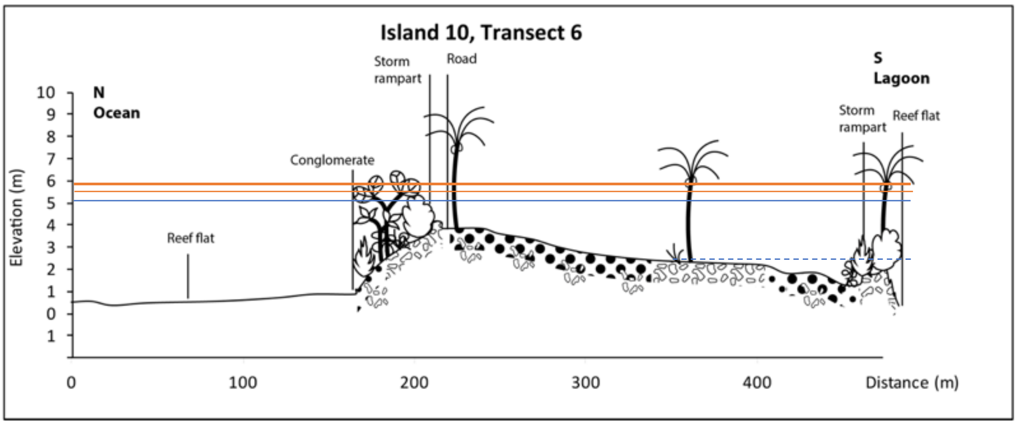
Island 10, Transect 6
N
Ocean
Storm rampart
Road
Conglomerate
Reef flat
S
Lagoon
Storm rampart
Reef flat
Elevation (m)
Distance (m)

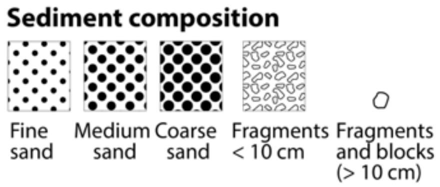
Sediment composition
Fine sand
Medium sand
Coarse sand
Fragments < 10 cm
Fragments and blocks (> 10 cm)

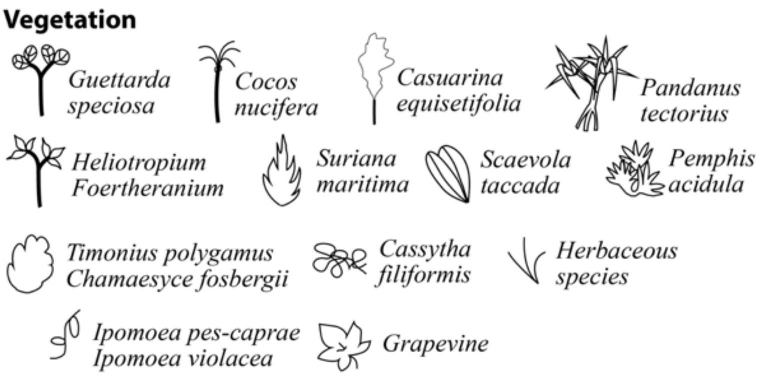
Vegetation
Guettarda speciosa
Cocos nucifera
Casuarina equisetifolia
Pandanus tectorius
Heliotropium Foertheranium
Suriana maritima
Scaevola taccada
Pemphis acidula
Timonius polygamus
Chamaesyce fosbergii
Cassytha filiformis
Herbaceous species
Ipomoea pes-caprae
Ipomoea violacea
Grapevine

Island 11

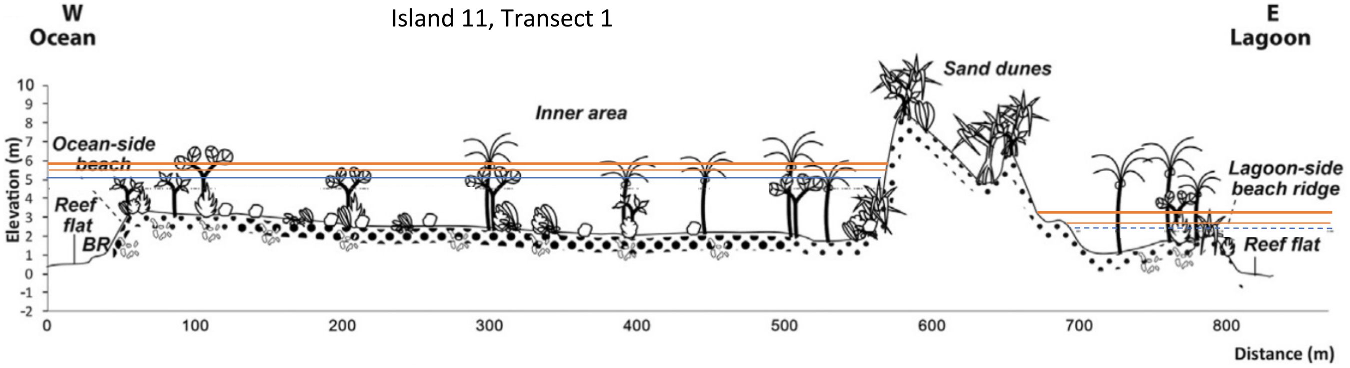
Island 11, Transect 1
W
Ocean
E
Lagoon
Sand dunes
Inner area
Ocean-side beach
Reef flat
BR
Lagoon-side beach ridge
Reef flat
Elevation (m)
Distance (m)

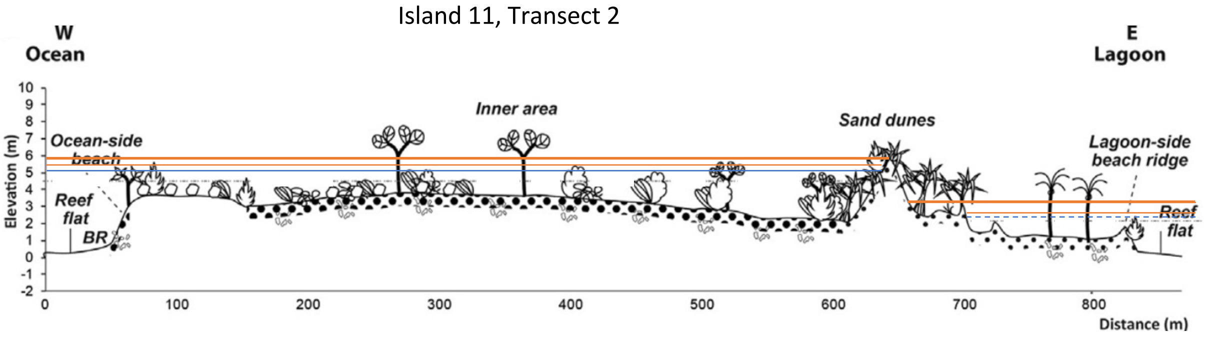
Island 11, Transect 2
W
Ocean
E
Lagoon
Inner area
Sand dunes
Ocean-side beach
Reef flat
BR
Lagoon-side beach ridge
Reef flat
Elevation (m)
Distance (m)

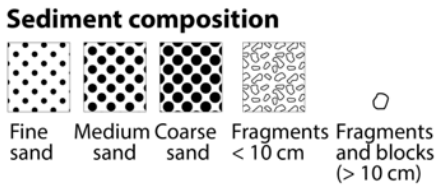
Sediment composition
Fine sand
Medium sand
Coarse sand
Fragments < 10 cm
Fragments and blocks (> 10 cm)

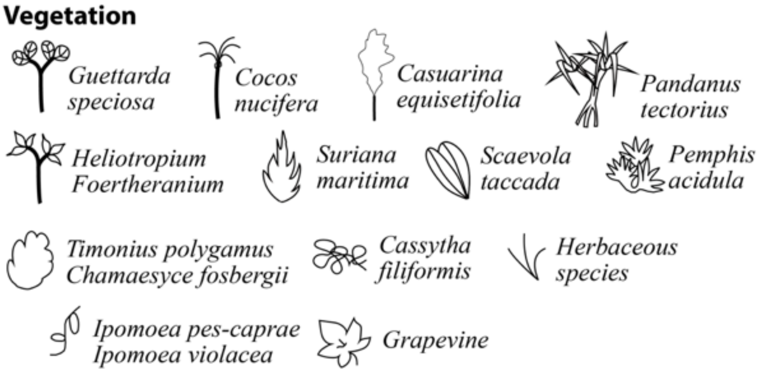
Vegetation
Guettarda speciosa
Cocos nucifera
Casuarina equisetifolia
Pandanus tectorius
Heliotropium Foertheranium
Suriana maritima
Scaevola taccada
Pemphis acidula
Timonius polygamus
Chamaesyce fosbergii
Cassytha filiformis
Herbaceous species
Ipomoea pes-caprae
Ipomoea violacea
Grapevine

Island 12

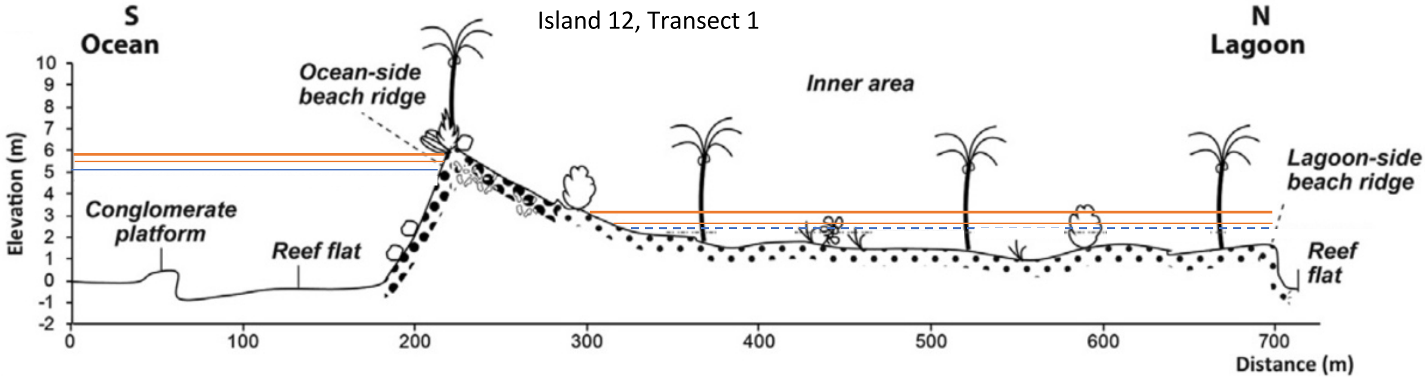

S
Ocean
Island 12, Transect 1
N
Lagoon
Ocean-side beach ridge
Inner area
Lagoon-side beach ridge
Conglomerate platform
Reef flat
Reef flat
Elevation (m)
Distance (m)

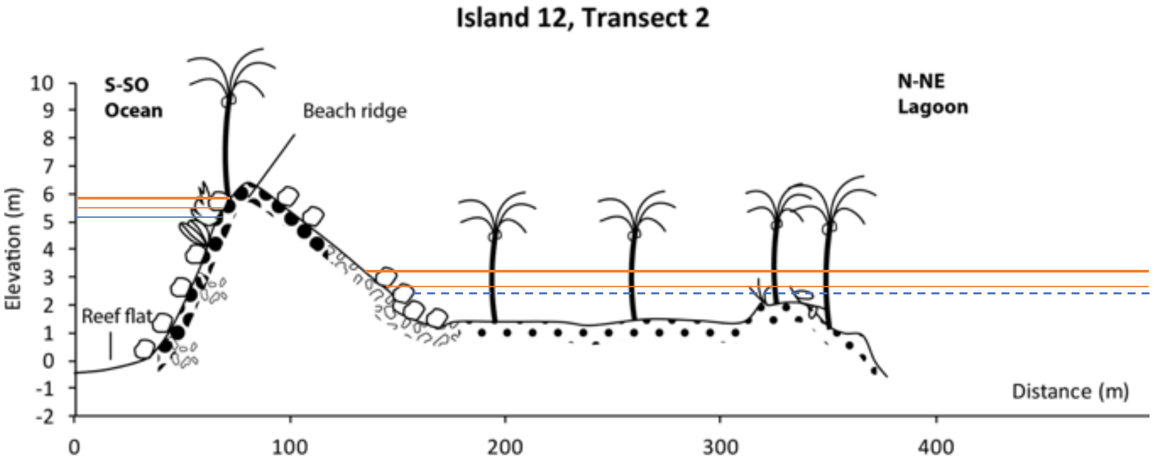

Island 12, Transect 2
S-SO
Ocean
Beach ridge
N-NE
Lagoon
Reef flat
Elevation (m)
Distance (m)

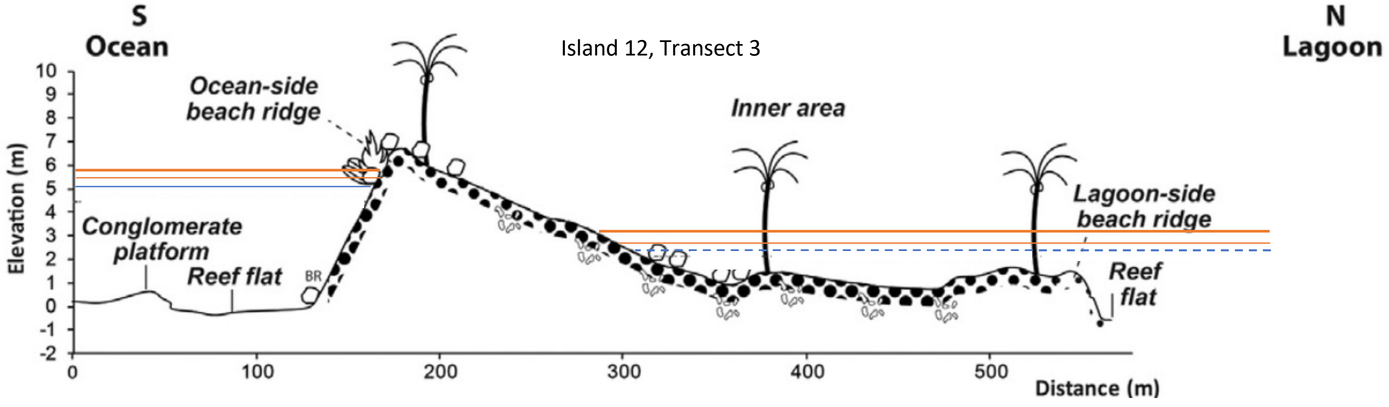

S
Ocean
Island 12, Transect 3
N
Lagoon
Ocean-side beach ridge
Inner area
Lagoon-side beach ridge
Conglomerate platform
Reef flat
BR
Reef flat
Elevation (m)
Distance (m)

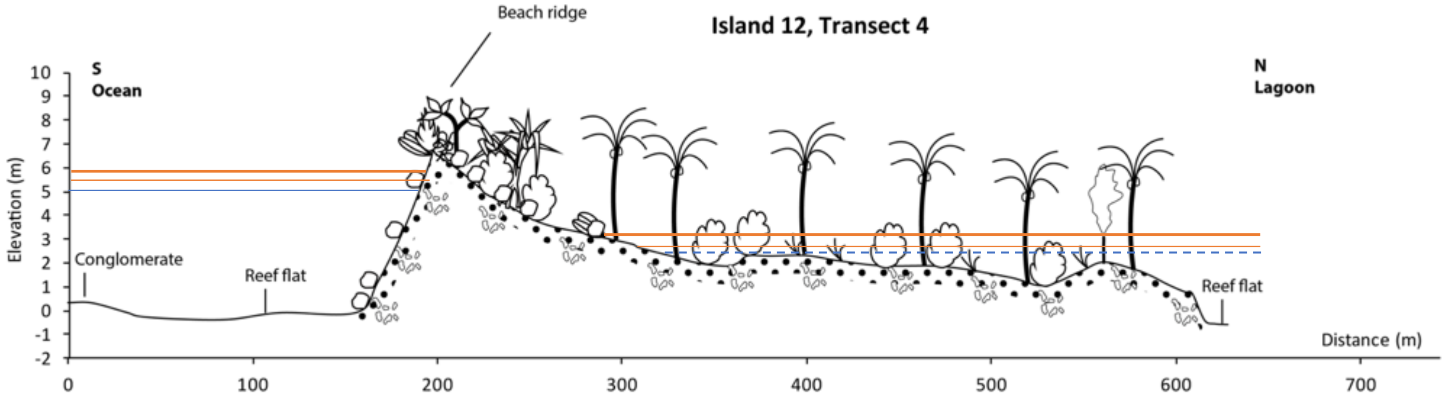

Beach ridge
Island 12, Transect 4
S
Ocean
N
Lagoon
Conglomerate
Reef flat
Reef flat
Elevation (m)
Distance (m)

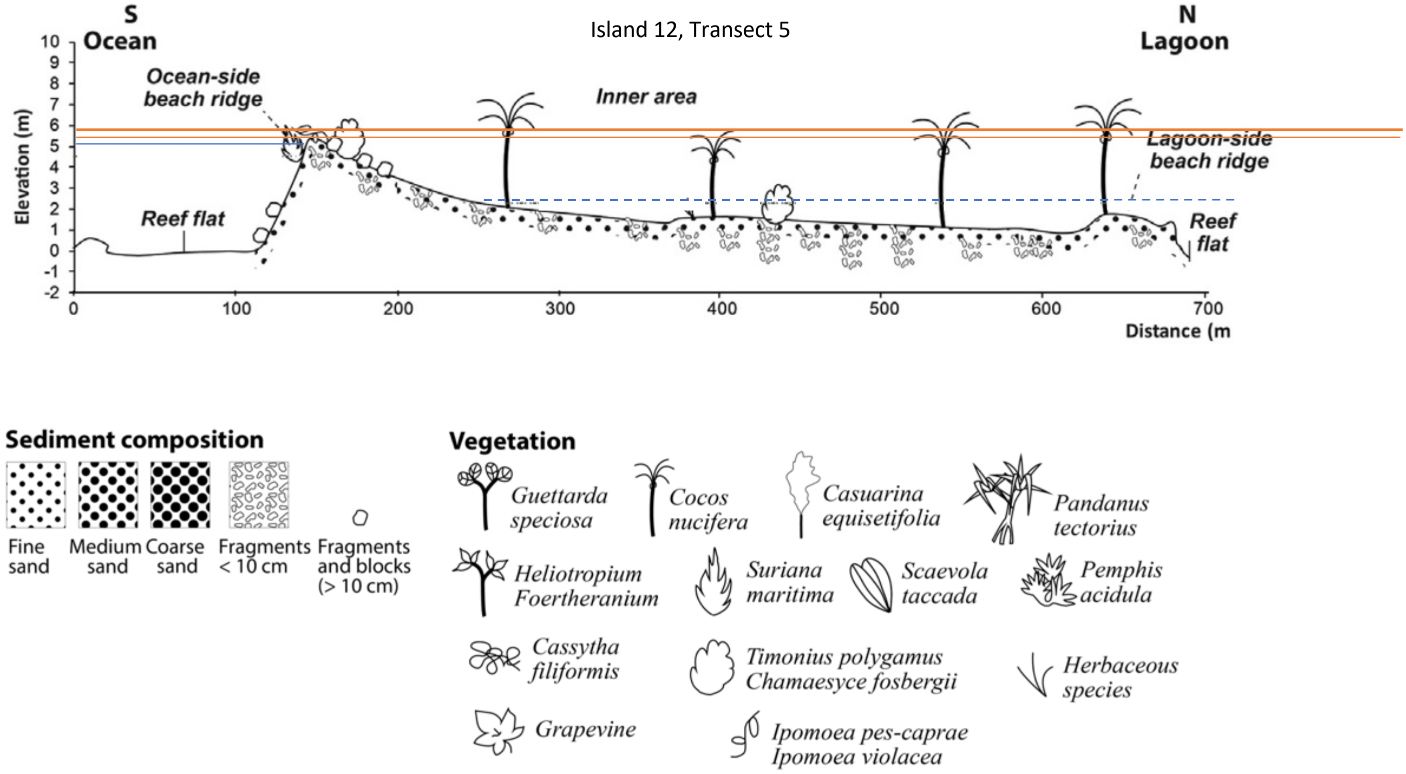

Island 12, Transect 5
S
Ocean
N
Lagoon
Ocean-side beach ridge
Inner area
Lagoon-side beach ridge
Reef flat
Reef flat
Elevation (m)
Distance (m
Sediment composition
Fine sand
Medium sand
Coarse sand
Fragments < 10 cm
Fragments and blocks (> 10 cm)
Vegetation
Guettarda speciosa
Cocos nucifera
Casuarina equisetifolia
Pandanus tectorius
Heliotropium Foertheranium
Suriana maritima
Scaevola taccada
Pemphis acidula
Cassytha filiformis
Timonius polygamus
Chamaesyce fosbergii
Herbaceous species
Grapevine
Ipomoea pes-caprae
Ipomoea violacea

Supplementary Material 7. Coastal risk policy analysis

This Supplementary Material summarizes the methodology developed for the analysis of the climate change integration challenge in French Polynesia (see ref. 65 in the main reference list).

Semi-structured interviews

42 semi-structured interviews were carried out between mid-December 2018 and mid-February 2019 with the main French Polynesian institutions involved in the design and implementation of the above-described policy documents as well as key informants concerned with coastal risk management and/or land use and urban planning. These semi-structured interviews relied on a series of open questions to allow interviewees to express personal views —though fed by their professional experience— on current and future coastal risk management and practices:

(i) Understanding the level of detail considered in the studied policy documents on coastal erosion and marine flooding. Related questions were: *Are these hazards specifically mentioned? If so, are their major natural and anthropogenic drivers considered? Does the document(s) set clear objectives for erosion and/or flooding risk reduction; that is, are objectives formulated, and possibly quantified and time-bounded?* And *have indicators for monitoring results been established?*

(ii) Asking about the explicit consideration of extreme events generating erosion and flooding (especially tropical cyclones) as well as of sea-level rise projections (global or relative, scenarios considered).

(iii) The consideration of uncertainty on local impacts. One central question was: *Regarding marine flooding, does the policy document refer to a single scenario (e.g. low- or high-end) or to a range of scenarios (for example to account for the variability of potential sea-level rises or even flooding limits/areas)?*).

(iv) The potential to readjust existing erosion/flooding risk reduction strategies and include a longer-term perspective. The underlying questions (asked for both erosion and marine flooding) were: *With respect to the short term, does the political/institutional framework allow for readjustment of erosion/flooding control strategies in case an extreme event occurs (e.g. responding rapidly to an erosion peak/flooding event), and according to local specificities?* And *on a longer-term perspective, does the political/institutional framework allow for readjustment of erosion/flooding control strategies in light of new knowledge (e.g. observed or projected se-level extremes)?*

(v) Institutional capacities to have a cross-institution dialogue and ability to drive policy adjustments. This touches on multiple and complex dimensions, so here we only focused on proxy information through the following questions: *Is a cross-cutting approach involving at least key ministries in charge of coastal risk management related issues (erosion and flood risk in particular) in place? What about the frequency of such meetings?* And *are precise objectives settled for these meetings in terms of the elaboration or implementation or revision of the document, including specifically with respect to coastal erosion and marine flooding.*

Coastal risk reduction policy document analysis

The study relies on the analysis of the most updated public policy documents dealing with planning coastal risk reduction, including those that deal with the coastal zone indirectly. The policy documents have been selected based on the framing used in Terorotua et al. (2020; ref. 64 in the main reference list) to identify key local public stakeholders *a priori* concerned with climate change. We considered a public policy document relevant when it addresses, even indirectly, coastal risks to people, infrastructures and/or economic activities, possibly including risk from climate change beyond only sea-level rise. We paid particular attention to the treatment of two coastal hazards, i.e. coastal erosion and marine flooding, and distinguished between public policy documents that apply to the French Polynesia territory as a whole and those that are designed for more local scale contexts (group of islands, island, municipality, etc.).

Two documents designed by the Collectivity and encompassing the whole French Polynesia territory are considered: *(i)* the Climate and Energy Plan (*Plan Climat Énergie de la Polynésie française*) that frames the general climate strategy of the country in terms of both mitigation and adaptation; *(ii)* and the General Land Use Scheme (*Schéma d'Aménagement Général*) that defines the overall development strategy in terms of demography and economy at a 20-year timescale. Two types of documents with a more local-scale focus are also considered: *(iii)* the General Land Use Plans (*Plan Général d'Aménagement*) that describes, for a given municipality, a 3-to-10 year development strategy through the identification of specific functions for the different areas within the municipality, i.e. for settlement (residential buildings and public infrastructures), economic activities, or natural areas (e.g. protected areas and cultural sites); *(iv)* the second Risk Prevention Plans (*Plan de Prévention des Risques*), which are actually the most prominent documents, once adopted, to address risk prevention and reduction at the local scale, especially through the characterization of areas where future constructions or activities are subject to specific rules or are prohibited, and where existing constructions must adhere to specific measures.

In terms of the analysis of these policy documents, desktop research essentially consisted in manually searching information related to two important dimensions to analyse the climate change policy integration challenge: *Coastal risk integration* and *Adjustability*.
Coastal risk integration refers to the extent to which policy documents consider the natural and anthropogenic drivers of climate-related coastal risks, as this is here hypothesized to reflect whether the understanding of current hazards and impacts, including their drivers of change, is a core element or a secondary concern in decision-making and planning. The main assessment variable (see Table 1) relates to the level of consideration of coastal erosion and marine flooding, including non-hazard risk drivers relating to exposure and vulnerability. The sub-variables listed in the right column of Table 1 have inspired by recent works highlighting the need to integrate scientific knowledge of flood risk (Pasquier et al. 2020) and to refer to precise objectives in order to be in position of informing policy processes (Runhaar et al. 2018).

Adjustability refers to the capacity of the French Polynesia State- and Collectivity-driven policy documents to consider local specificities (e.g. differences in coastal configurations and dynamics in high and low-lying islands), and refine progressively coastal erosion and marine flooding control targets and strategies either after an extreme event (short-term perspective)

or according to new scientific knowledge raised on future climate change-induced risks (anticipation for the longer-term). Some information on cross-institutional dynamics at work is also considered as enabling conditions for adjustability. Finally, P2 includes the extent to which the public policy documents consider uncertainty of future changes in hazard patterns (trend, rate, geographical distribution), especially through the consideration of a range of coastal climate risk scenarios.

Practically, the desktop analysis consisted of mapping, for each of the policy document considered, the extent to which the assessment variables above were met. Two of the authors worked on this mapping, independently from each other, and used a three level framing (estimated low, medium, high match) associated with detailed justification based on their reading of the policy documents and feedbacks from the interviews (see Fig. 3 in ref. 65). A group discussion then took place to allow to create a cross check between the two independent assessments, which actually aligned with each other on all the assessment variables.

References cited (and listed in the main reference list)

62. Terorotua, H., Duvat, V.K.E., Maspataud, A., Ouriqua, J. Assessing Perception of Climate Change by Representatives of Public Authorities and Designing Coastal Climate Services: Lessons Learnt From French Polynesia. *Front. Mar. Sci.* **7**, 160 (2020). https://doi.org/10.3389/fmars.2020.00160.

65. Magnan, A.K., Viriamu, T., Moatty, A., Duvat, V.K.E., Le Cozannet, G., Stahl, L., Anisimov, A. The climate change policy integration challenge in French Polynesia, Central Pacific Ocean. *Reg. Environ. Change* (forthcoming). *Reg. Environ. Change* **22**, 76 (2022). https://doi.org/10.1007/s10113-022-01933-z
